# HLA-II peptide-binding diversity shapes humoral immune responses and susceptibility to infections

**DOI:** 10.1038/s41467-026-76184-1

**Published:** 2026-08-05

**Authors:** Bettina Magyari, Anna Tácia Fülöp, Dávid Kókai, Franciska Tóth, Gergő Mihály Balogh, Sergio Andreu-Sánchez, Gabriel Innocenti, Rinse K. Weersma, Jingyuan Fu, Csaba Pál, Alexandra Zhernakova, Thomas Vogl, Máté Manczinger

**Affiliations:** 1 Synthetic and Systems Biology Unit, Institute of Biochemistry, HUN-REN Biological Research Centre, Szeged, Hungary; 2 Doctoral School of Biology, University of Szeged, Szeged, Hungary; 3 HCEMM-BRC Systems Immunology Research Group, Szeged, Hungary; 4 Center for Cancer Research, Medical University of Vienna, Vienna, Austria; 5 Department of Genetics, University of Groningen, University Medical Center Groningen, Groningen, the Netherlands; 6 Department of Pediatrics, University of Groningen, University Medical Center Groningen, Groningen, the Netherlands; 7 Department of Dermatology and Allergology, University of Szeged, Szeged, Hungary

**Keywords:** Infectious diseases, Immunogenetics, Infection, Humoral immunity

## Abstract

Infectious diseases represent a leading cause of mortality worldwide and have shaped the evolution of the human immune system. Human leukocyte antigen (HLA) class II molecules are key to the humoral immune response by presenting peptides to helper T cells. The peptide-binding range varies greatly among HLA-II variants, but the role of this variation in humoral immunity and infection susceptibility has remained unexplored. Here, we integrate data on HLA-II immunopeptidomics and antibody responses in ~1500 individuals and demonstrate that a broad peptide-binding repertoire of HLA-II is linked to antibody production against specific pathogen-associated proteins and more favorable outcomes in several common infections. In the UK Biobank, individuals carrying such HLA-II molecules had a reduced risk of severe infections and up to a 45% lower incidence of diseases caused by common pathogens. These findings suggest that the peptide diversity presented by HLA-II variants shapes humoral immunity and can serve as a risk factor for infection susceptibility.

## Abstract (web summary)

Human leukocyte antigen class II (HLA-II) are involved in the presentation of peptides to helper T cells which guide in the B cell response and antibody production. Here Magyari et al, show that HLA-II peptide binding diversity impacts the humoral immune response and susceptibility to infection.

## Introduction

**Fig. 1:**
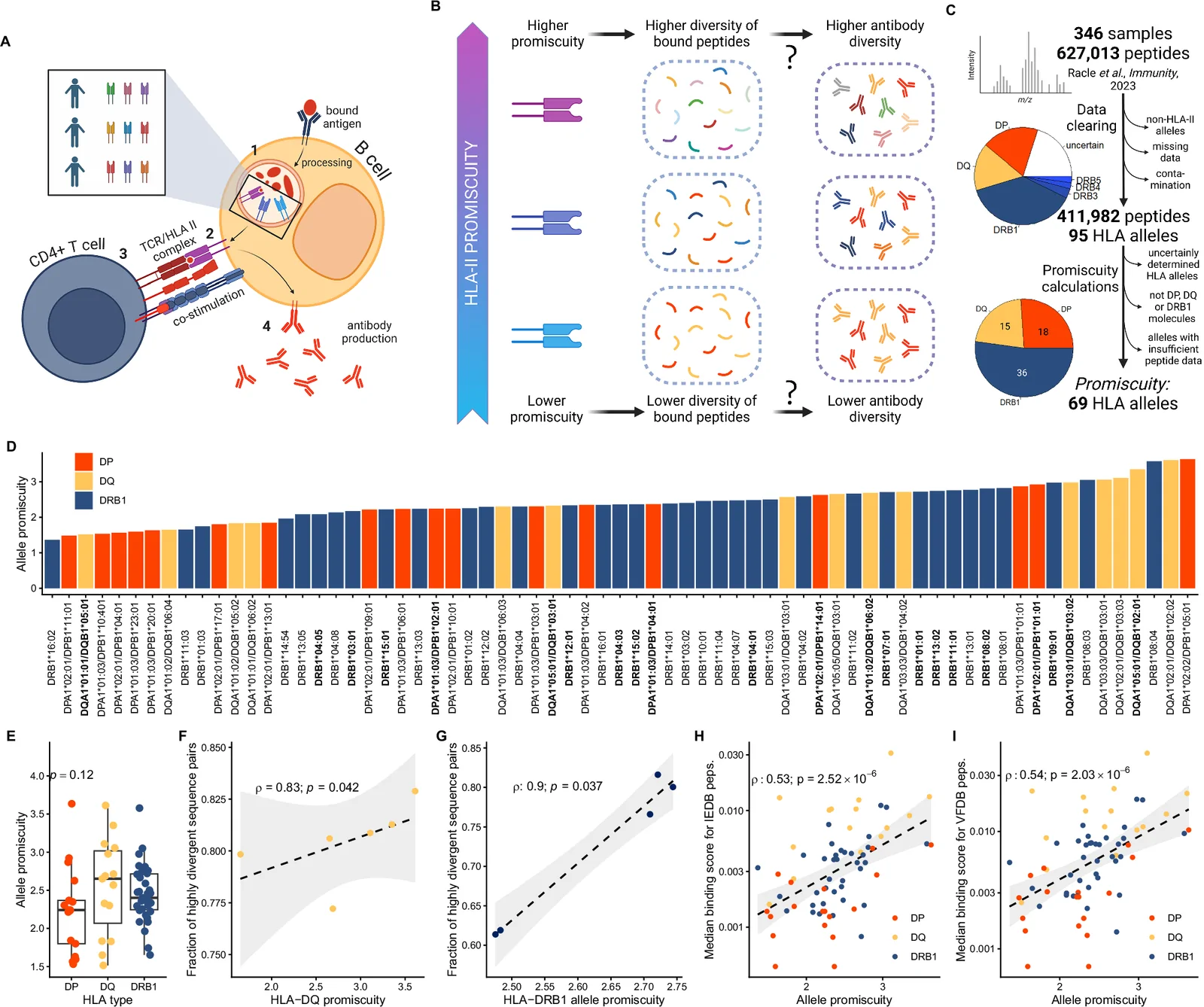
HLA-II promiscuity calculated from comprehensive immunopeptidomics data accurately predicts peptide-binding. **A** Antigen presentation by HLA-II molecules impacts antibody production. Once internalized, the BCR-bound antigen is broken down into peptides (1). These peptides are then presented on the surface of B cells by HLA-II molecules (2), a crucial step for the activation of CD4+ T cells (3). The activated T cells, in turn, facilitate the production of highly potent antibodies (4). Created in BioRender. Manczinger, M. (2026) https://BioRender.com/t8rgtwe**B** Our hypothesis suggests that HLA-II *Pr* may be linked to the humoral immune response to pathogens. HLA-II alleles with elevated *Pr* are capable of presenting a broader array of peptides, which could enhance the generation of antibodies against a wider variety of pathogens. Created in BioRender. Manczinger, M. (2026) https://BioRender.com/t8rgtwe**C** HLA-II *Pr* values were calculated from a detailed immunopeptidomics dataset. After thorough data cleaning and selection of HLA-II molecules with adequate data, we determined *Pr* for 69 HLA-II alleles. Created in BioRender. Manczinger, M. (2026) https://BioRender.com/t8rgtwe**D** HLA-DP, DQ and DRB1 molecules exhibit a wide range of promiscuity levels. Common alleles according to refs. ^20,21^ are highlighted with bold characters (see text for details). **E**) *Pr* of HLA-DP (*n* = 18 alleles), DQ (*n* = 15 alleles), and DRB1 (*n* = 36 alleles) do not display systematic differences. Two-sided *p*-value of a Kruskal-Wallis test is indicated. The horizontal lines indicate median, boxes indicate interquartile range (IQR) and vertical lines indicate first quartile −1.5 × IQR and third quartile +1.5 × IQR. **F-I**) Validation of *Pr* on independent datasets. **F**,**G** *Pr* is significantly associated with the diversity of presented AAV capsid peptides, identified in an independent study^22^. The vertical axis indicates the fraction of highly dissimilar (BLOSUM62 similarity <0) peptide core sequence pairs in the presented peptide repertoire of HLA-DQ (**F**) and HLA-DRB1 (**G**) molecules. The results remained when we defined diversity as the median of core sequence similarity values (Supplementary Fig. 3). **H**,**I** *Pr* is positively correlated with the binding of pathogen-associated epitopes in the IEDB (H) and VFDB (I). *n* = 18, 15 and 36 alleles for HLA-DP, DQ and DRB1, respectively. On panels F to I, Spearman’s correlation coefficients and *p*-values of two-sided correlation tests are shown, dashed blue lines indicate linear regression lines and gray area represents 95% confidence interval.

Infectious diseases remain one of the major causes of mortality worldwide^1^. A critical component of the adaptive immune defense against infections is the presentation of antigens to T cells, a process mediated by HLA molecules. HLA class II (HLA-II) molecules play a pivotal role in orchestrating the humoral immune response^2^. Following the recognition of antigens by B cells, the antigen-B cell receptor complex undergoes internalization, and within the lysosome, the protein sequence of the antigen is enzymatically cleaved into peptides (Fig. 1A). Subsequently, a subset of these peptides is selectively bound by HLA-II molecules and transported to the cell surface for presentation. Recognition of the peptide-HLA-II complex by CD4+ T helper cells is essential for initiating the activation and proliferation of B cells^2^. The human population contains a diverse set of approximately 10,000 HLA-II alleles, each with varying peptide-binding specificities, encoded by three main loci: HLA-DP, HLA-DQ, and HLA-DR^3^. Previously, we showed that variation in the HLA-II region is the strongest genetic determinant of the antibody repertoire^4^.

Traditionally, associations between HLA-II and diseases are determined by comparing allele prevalence in healthy and affected individuals. These studies have identified correlations between individual HLA-II alleles and the risk of various infectious diseases^5–7^. However, the high allelic diversity and strong linkage disequilibrium among HLA loci present significant challenges, often limiting the fine-mapping and robustness of these findings^8–10^. Other methods have relied on the homozygosity/heterozygosity or the evolutionary divergence of HLA alleles (HED^11,12^), which only indirectly represent their peptide-binding specificities. While novel approaches that incorporate the functional binding of HLA-I alleles have enhanced the prediction of viral load in HIV patients^13^, the exact role of HLA-II peptide-binding specificity in influencing the general susceptibility to infectious diseases remains largely unclear.

HLA-II variants display significant variability in the range of peptides they present, a characteristic known as peptide-binding promiscuity^14,15^. Some variants, termed ‘generalists’, can bind to a wide range of peptide sequences, while others, called ‘specialists’, have more limited binding profiles (Fig. 1B). So far, the implications of peptide-binding promiscuity for the risk of infectious diseases have remained unclear.

Here, we hypothesized that generalist HLA-II molecules provide protection against a variety of infectious diseases by affecting antibody responses. We tested this hypothesis with an approach that incorporates the peptide-binding specificity of different HLA-II alleles and data on antibody responses against thousands of antigens in ~1500 individuals from the LifeLines DEEP and 1000IBD population cohorts^16^. We found that individuals with HLA-II molecules exhibiting broad peptide-binding repertoires are more likely to mount a humoral immune response specifically targeting pathogen-associated peptides, rather than non-pathogenic ones. By utilizing the data from the UK Biobank, we also demonstrated that individuals carrying HLA-II variants with broad peptide-binding repertoires are less susceptible to various common infections.

## Results

### HLA-II peptide-binding promiscuity demonstrates wide variation between alleles

First, we aimed to determine the promiscuity level of different HLA-II variants by evaluating the diversity of peptides each allele can bind. Unless otherwise specified, we use HLA alleles as shorthand for their corresponding HLA proteins in line with previous studies^17,18^. HLA-II molecules predominantly bind peptides of 12–20 amino acids in length^19^. A nine–amino acid core segment sits in the binding groove, while flanking residues extend beyond it. We utilized the data from a recent immunopeptidomics study, which includes details on 627,013 peptides identified across 346 samples, including the corresponding core segment for each peptide (Fig. 1C, ref. ^19^). Building on our prior research^15^, we used the promiscuity index (*Pr*) to quantify the diversity of peptides presented by each HLA-II allele (see Methods for details). Briefly, *Pr* measures how closely the amino acid composition of bound peptides matches the natural amino acid distribution. Lower divergence suggests that an HLA allele is less selective, indicating higher peptide-binding promiscuity. Alleles with high *Pr* values can bind a broader range of peptides. Note that for HLA-DP and -DQ molecules, both the α- and β-chains are polymorphic, whereas for HLA-DRB1 the α-chain is essentially invariant and peptide binding is determined solely by the β-chain. Accordingly, as in other studies^19^, when we refer to “alleles,” we mean α–β chain combinations for DP and DQ, but β-chain variants only for DRB1.

The size of the immunopeptidomics dataset allowed for the calculation of *Pr* for 18 DP, 15 DQ, and 36 DRB1 alleles (Fig. 1D). To evaluate the prevalence of these alleles across human populations, we used a comprehensive reference list of HLA-II alleles that cover a large fraction ( > 99%) of the individuals in human populations^20^. Encouragingly, we could determine the promiscuity index for 89% of these alleles. Moreover, our allele set with *Pr* values included all nine HLA-DRB1 alleles identified as universally frequent or very frequent in human populations^21^. *Pr* showed substantial variation across common alleles, yet did not display any systematic differences between the DP, DQ, and DRB1 groups (Fig. 1E). To assess the robustness of the *Pr* values and minimize potential bias arising from differences in experimental setups across immunopeptidomics studies, we recalculated *Pr* using two independent subsets of the original dataset (*n* = 102 and *n* = 21 samples). Although the samples originated from different sources, all raw data were processed with the same bioinformatics pipeline^19^. The Pr values derived from the two subsets showed a strong correlation (Supplementary Fig. 1), indicating that our estimates are consistent and not driven by study-specific experimental conditions. The reliability of the calculated *Pr* values was further validated using data from two independent immunopeptidomics studies, which focused on HLA-II-presented peptides from adeno-associated virus (AAV) and SARS-CoV-2^22,23^. We found that higher HLA-II *Pr* is positively correlated with the diversity of the presented peptides in both datasets (Fig. 1F, G and Supplementary Figs. 2, 3).

Next, we aimed to test whether HLA-II alleles with high *Pr* are likely to bind more pathogen-associated epitopes. For this purpose, we used the NetMHCIIpan-4.3 algorithm^24^ to assess the binding of overlapping 15-mer peptides from 272 protein antigens listed in the Immune Epitope Database (IEDB,^25^) and from 444 pathogen-associated proteins in the Virulence Factor Database (VFDB,^26^) for each allele. HLA-II *Pr* showed a significant positive correlation with the median of the predicted binding scores (Fig. 1H, I). In summary, the findings suggest that *Pr* effectively captures the breadth of the peptide-binding repertoire of HLA-II alleles.

### Promiscuity is linked to the specificity of HLA-II alleles

**Fig. 2:**
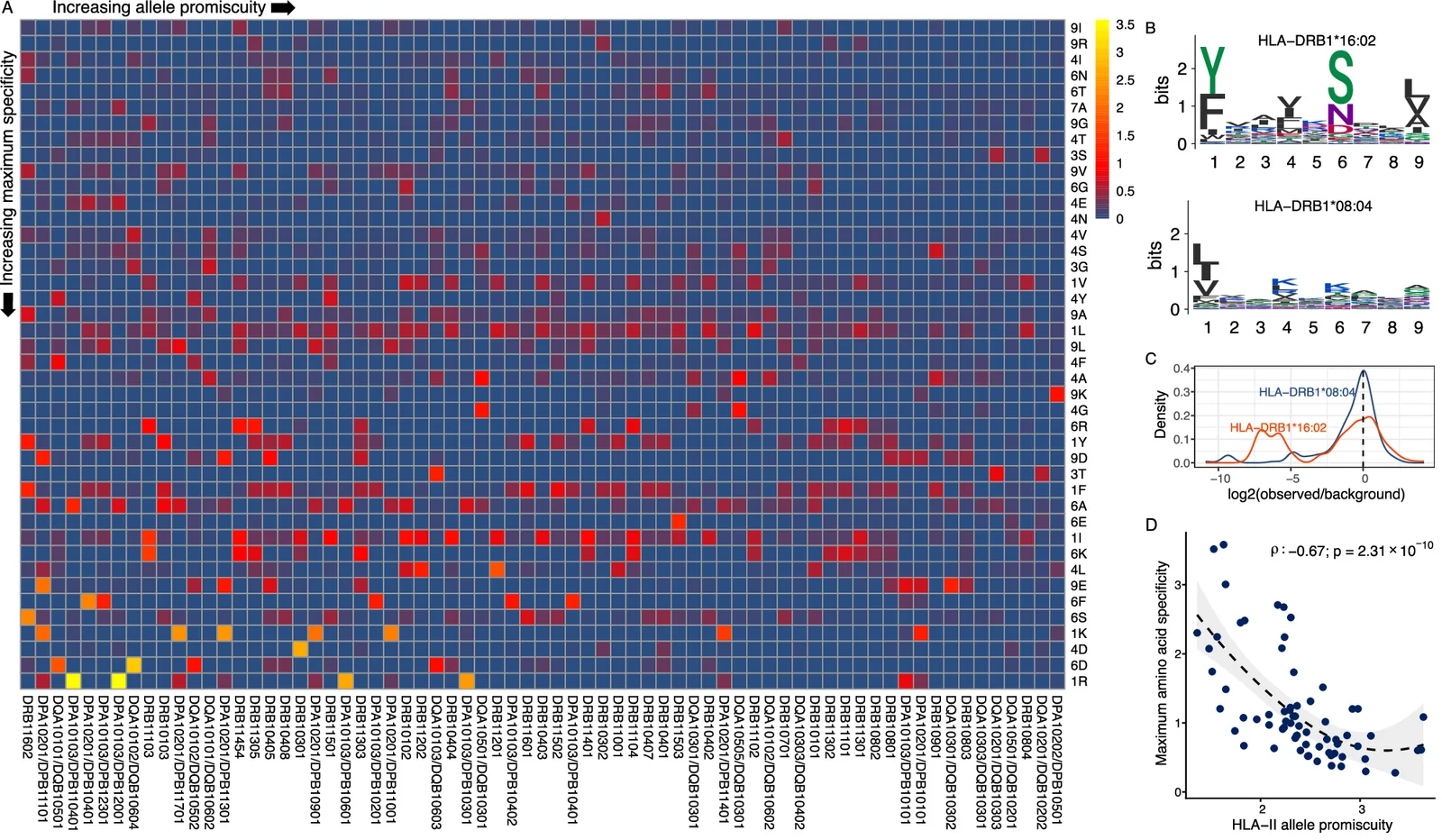
Pr captures the amino acid specificity of HLA-II alleles. **A** The heatmap indicates the specificity of HLA-II molecules for different amino acids at different positions of the binding core color-coded. HLA molecules are ordered by increasing *Pr*. The values represent information-content values (in bits) in binding logos (see Methods for details). The first and second characters in row names indicate positions and amino acids, respectively. Rows are ordered by the maximum specificity value for the given amino at the given position. Only those position-amino acid combinations are shown that reach at least 0.4 for at least one HLA-II allele. **B** Representative binding logos of HLA-II alleles with high (bottom) and low (top) *Pr*. The vertical axes indicate information content in bits. **C** The plot shows the density of log-odds ratio values for the same alleles as in (**B**). **D** Alleles with high *Pr* exhibit generally low amino acid specificity. The maximum amino acid specificity, defined as the maximum value in the sequence logo is shown as a function of *Pr* (*n* = 69 alleles). Spearman’s correlation coefficient and *p*-value of a two-sided correlation test are indicated, the dashed blue line indicates a linear regression line, and the gray area represents 95% confidence interval.

To explore the mechanistic basis of variation in promiscuity level across HLA variants, we analyzed their binding motifs. The binding motifs capture amino acid preferences within the binding core region, with the anchor positions (typically 1, 4, 6, and 9) showing the highest specificities^19^. To generate the binding motifs, we used the same dataset as for calculating the *Pr* values. HLA-II variants with high promiscuity levels displayed binding motifs with generally low specificity for most amino acids (Fig. 2A, B). In contrast, variants with lower promiscuity levels were more selective, showing distinct preferences for specific amino acids at key anchor positions. For example, HLA-DRB1*16:02, which had the lowest promiscuity index, was highly selective for phenylalanine (F) and tyrosine (Y) at the 1^st^ position and for serine (S) at the 6^th^ position of the core region of the presented peptide (Fig. 2B). On the other hand, HLA-DRB1*08:04, which had the highest *Pr* among HLA-DRB1 variants, showed minimal selectivity for amino acids at anchor positions (Fig. 2B). We also calculated log-odds ratio values for each amino acid at each position, defined as the log₂ of the amino acid frequency at that position divided by its background probability (i.e. its expected frequency under the background distribution). For the highly promiscuous HLA-DRB1*08:04 allele, most values clustered tightly around zero (Fig. 2C), indicating that amino acid usage at most positions is close to the background distribution. In contrast, the low-promiscuity HLA-DRB1*16:02 allele showed a broader distribution with a pronounced peak at negative values, reflecting stronger deviations from background at specific positions. To further investigate the relationship between the promiscuity and specificity of HLA-II variants, we determined the highest value for any amino acid in the binding motif of each HLA-II variant and used this value as a proxy for allele specificity. As expected, there was a strong negative correlation between the specificity and promiscuity of HLA-II molecules (Fig. 2D). Overall, these findings demonstrate that *Pr* effectively captures biologically relevant properties of HLA-II alleles.

### High HLA-II promiscuity is linked to the presence of antibodies targeting pathogens

**Fig. 3:**
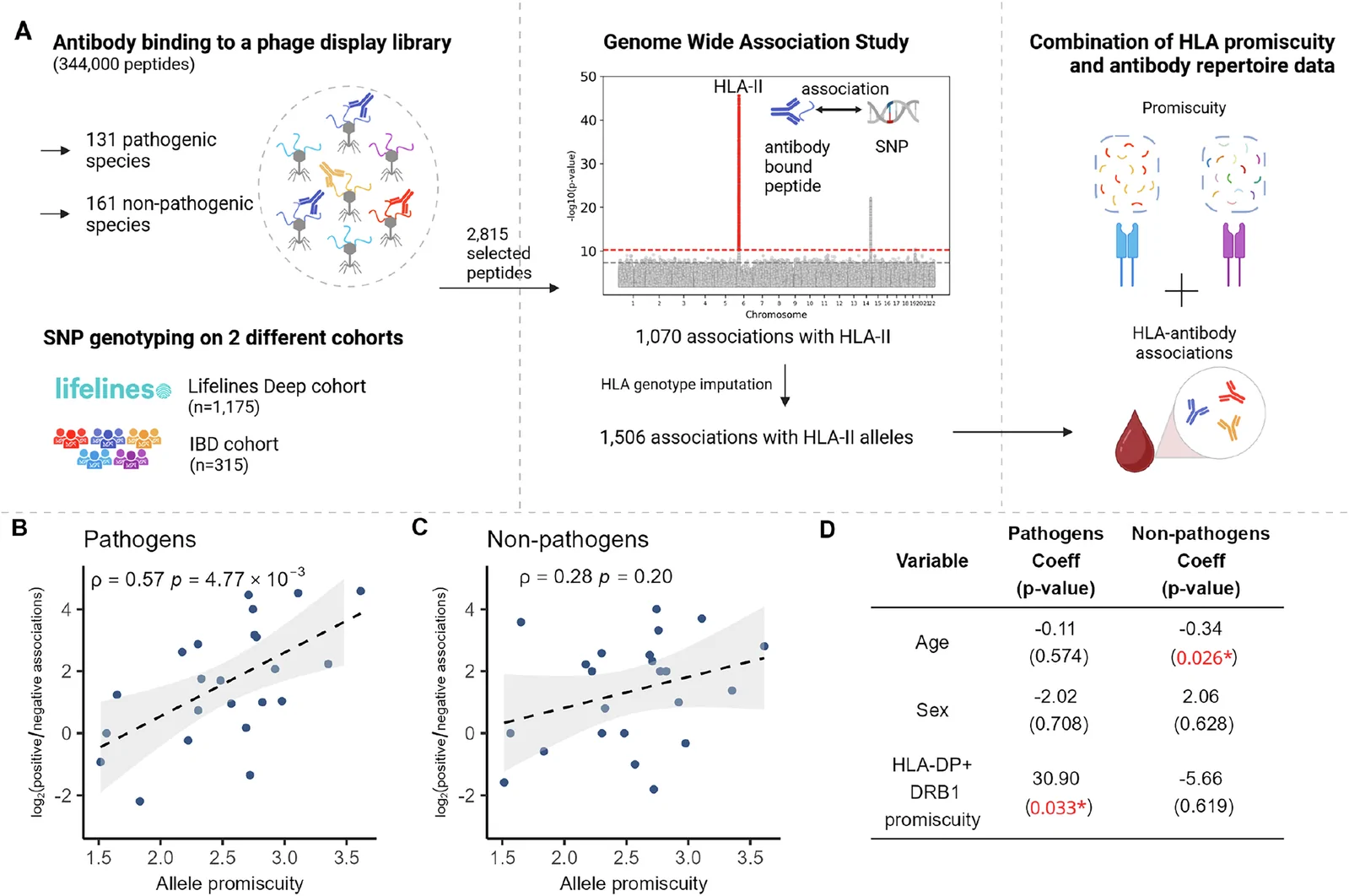
HLA-II promiscuity is linked to the presence of antibodies targeting pathogen-associated proteins. **A** Generation and composition of the HLA-II association dataset in ref. ^4^. Created in BioRender. Manczinger, M. (2026) https://BioRender.com/rv0o8n4**B**,**C** Promiscuous HLA-II alleles are positively associated with antibodies targeting pathogens but show no association with antibodies targeting non-pathogens. The binary logarithm of the ratio of positive/negative associations for pathogens (**B**) and non-pathogens (**C**) is shown as a function of HLA-II *Pr* (n = 2 HLA-DP, 11 HLA-DQ and 10 HLA-DRB1 alleles). To prevent division by zero, 1 was added to both the numerator and the denominator. Spearman’s rho values and p-values of two-sided correlation tests are indicated, dashed black lines indicate linear regression lines and gray area represents 95% confidence interval. **D** HLA-II *Pr* is linked to the presence of pathogen-specific antibodies in the blood of individuals. The table summarizes the mixed-effects linear model for pathogens and non-pathogens (*n* = 916 individuals in both models). Model coefficients are shown with *p*-values of two-sided Wald *t*-tests in parentheses. The cohort to which an individual belonged (LifeLines DEEP or IBD) was included in the model as a random effect. Significant *p*-values are highlighted with red color. Note that the model did not involve HLA-DQ promiscuity as it covered individuals insufficiently.

The significant role of microbial infections in the evolution of HLA-II molecules is well-established^27^, and they may have also driven the evolution of HLA-II promiscuity^14,27^. We hypothesized that highly promiscuous HLA-II molecules are associated with humoral immune responses to a broader range of pathogens, but not to non-pathogenic microbes. To investigate this hypothesis, we employed a large-scale dataset, which maps associations between HLA-II variants and the presence of antibodies against specific peptides^4^ (Fig. 3A). This dataset was derived from two studies^4,16^, where we measured the antibody epitope repertoires against 344,000 peptide antigens in 1175 HLA-genotyped healthy individuals and 315 IBD patients using phage display immunoprecipitation sequencing (PhIP-seq). PhIP-seq currently provides the most extensive and systematic dataset of linear epitopes, making it a practical and reliable proxy for high-throughput analysis. The epitopes analyzed here were curated from established resources, including the Immune Epitope Database (IEDB) and the Virulence Factor Database (see refs. ^25,26^ for details). 2815 antigens, with antibodies present in 5–95% of individuals, were included in further analysis, where we identified 1506 associations between antibody-bound antigens and HLA-II alleles^4^. 75% of associations were positive—where carrying the allele is linked to the presence of the antibody—and 25% were negative, where the allele is connected to the absence of the antibody. Numerous antigens in these associations were established targets of neutralizing antibodies. For example, antibodies against Streptolysin O, one of the best-characterized S. pyogenes antigens^28,29^, were negatively associated with HLA-DQA1*03:01. Notably, in the original study^4^, we found that for many positive associations, HLA-II molecules exhibit a distinct ability to present specific segments of antigens, underscoring the complex relationship between HLA-II alleles and immune recognition.

We focused on the identified positive and negative associations between antibody responses and HLA-II alleles. As expected, statistically significant associations were dominated by proteins from common pathogenic microbes endemic to the Netherlands according to the GIDEON database^14,30^ (2.16% and 0.98% of associations were significant for endemic pathogens and other taxa in the dataset, respectively. OR: 2.23, two-sided *p*-value of Fisher’s exact test <2.2 × 10^-16^). This finding is particularly relevant, as a high HLA-II promiscuity level could be favorable when individuals face commonly occurring pathogens^31^. On the other hand, HLA-II molecules with a low promiscuity level may specifically target evolutionarily novel and relatively rare pathogens^31^. As a result, it is reasonable to expect that high-*Pr* HLA-II alleles would show mostly positive associations with antibodies targeting common pathogens, while low-*Pr* HLA-II alleles would be more frequently linked to negative associations.

Given that the total number of associations positively correlates with allele frequency in the cohort (Supplementary Fig. 4A), we calculated the ratio of positive to negative associations (Supplementary Table 1). This ratio is independent of allele frequency (Supplementary Fig. 4B), providing an unbiased estimate. We classified each taxon in the dataset to pathogens (n = 131) and non-pathogens (*n* = 161) using the GIDEON database and literature information^14,30^ (Supplementary Table 2, see Methods for details). We found a significant correlation between HLA-II promiscuity level and the ratio of positive associations with antibodies targeting pathogen-associated proteins (Fig. 3B, Spearman’s correlation coefficient: 0.57; two-sided correlation test p-value: 0.005). The relationship remained significant in a linear regression model including three HLA-II groups (i.e., DP, DQ and DRB1) as a covariate (Supplementary Table 3). In contrast, no significant correlation was observed with non-pathogenic microbes (Fig. 3C, Spearman’s correlation coefficient: 0.28; p-value: 0.2). The above results also remained significant when considering HLA-II promiscuity at the individual rather than allelic level and controlling for age, sex, and cohorts (Fig. 3D). These findings highlight a strong association between HLA-II promiscuity and the humoral immune response against pathogens.

### High HLA-II promiscuity level protects from various infections

**Fig. 4:**
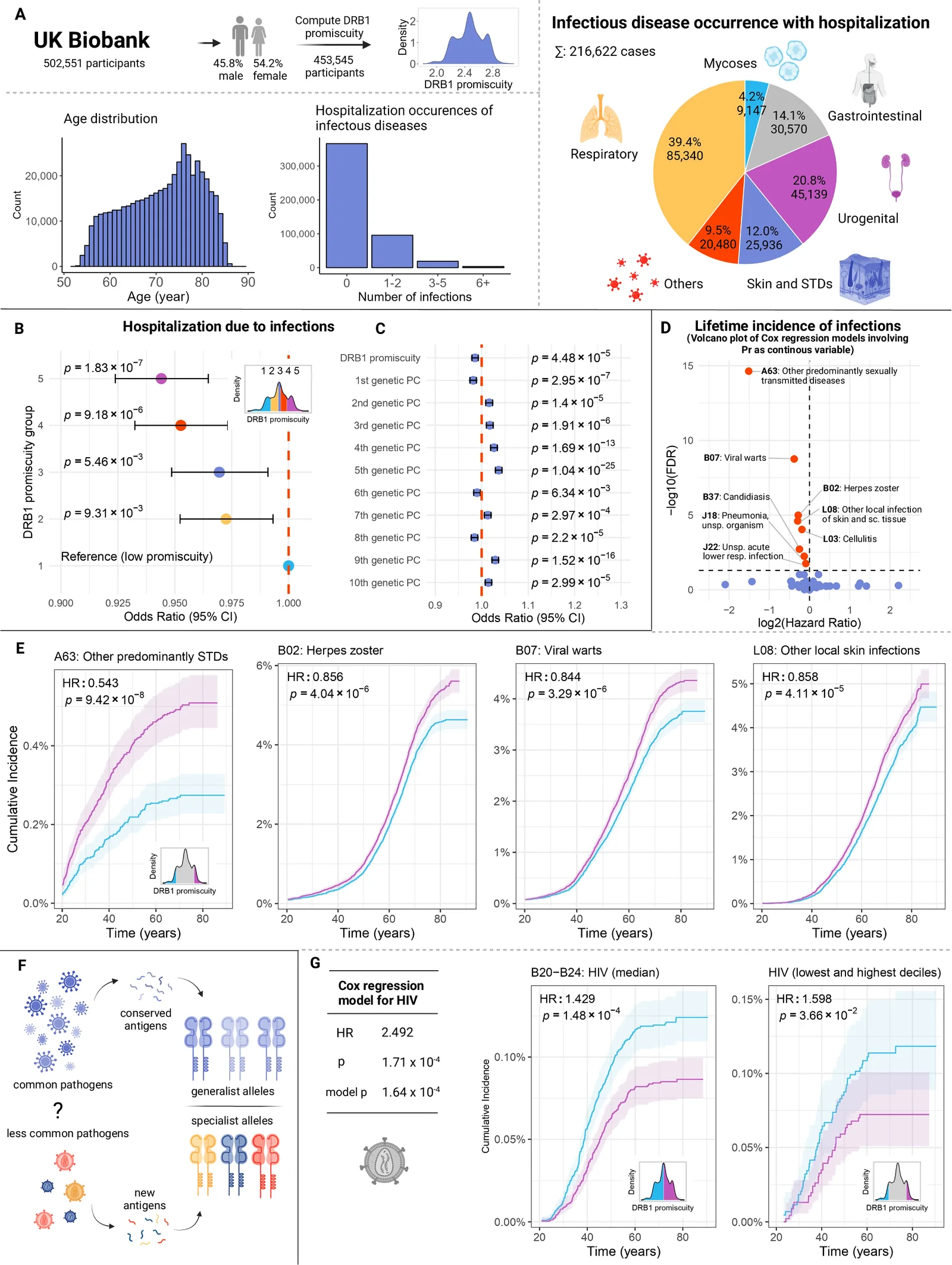
HLA-DRB1 promiscuity influences infectious disease susceptibility. **A** An overview of the UK Biobank cohort and the distribution of inpatient infectious disease diagnoses. We utilized HLA genotype data and electronic health records from the UK Biobank to calculate HLA-DRB1 promiscuity and examine its association with infectious diseases. Created in BioRender. Manczinger, M. (2026) https://BioRender.com/ayyarbi**B** Higher HLA-DRB1 promiscuity is linked to a lower prevalence for severe infections. Using quintiles, participants were classified into five similar-sized groups with increasing promiscuity levels (n = 91,459, 96,869, 84,464, 91,184, and 88,462 individuals from the lowest to the highest promiscuity group, respectively). The plot depicts the results of a logistic regression model. Points represent the odds ratio for groups with increasing promiscuity compared to the lowest promiscuity group. Horizontal lines indicate the corresponding 95% confidence interval (CI). **C** The impact of HLA-DRB1 promiscuity remains significant after adjusting for genetic principal components. The summary of a logistic regression model that includes the first ten genetic principal components as covariates (*n* = 452,438 individuals). Points represent the odds ratio, while horizontal lines depict the 95% CI. **D** Higher HLA-DRB1 promiscuity is associated with a lower hazard of multiple infections. The volcano plot shows the log2 of hazard ratios and the −log10 of adjusted p-values from Cox proportional hazards models for the incidence of various disorders, with HLA-DRB1 promiscuity treated as a continuous variable. p-values were corrected using the Benjamini-Hochberg method^55^. The horizontal dashed line represents 0.05 FDR value, significant values are highlighted with red color. *n* = 1676, 16,293, 14,712, 17,615, 12,217, 31,087, 36,694, and 59,797 individuals diagnosed with A63, B07, L08, B02, B37, L03, J18, and J22, respectively. For the full list of diseases and corresponding values, see Supplementary Table 4. On panels C and D, p-values of two-sided Wald z-tests are indicated. **E** HLA-DRB1 promiscuity groups are associated with up to approximately 46% lower hazard of infections. Representative plots show cumulative disease incidence for the four most significant associations in (**D**), comparing participants in the highest and lowest deciles of HLA-DRB1 promiscuity. The p-values of two-sided log-rank tests are indicated. 95% confidence intervals are indicated with background shading. HR: hazard ratio (high vs. low promiscuity groups). In the lowest decile group, *n* = 225, 1963, 1687, 1612 infected and 45,510, 43,772, 44,048, 44,123 non-infected individuals with A63, B02, B07, and L08, respectively. In the highest decile group, *n* = 116, 1586, 1345, 1304 infected and 43,291, 41,821, 42,062, 42,102 non-infected individuals with A63, B02, B07, and L08, respectively. **F** We hypothesize that generalist HLA-II alleles can present a broad range of antigens from common pathogens, while specialists bind substantially different antigens of less common, evolutionary novel pathogens^31^. Created in BioRender. Manczinger, M. (2026) https://BioRender.com/ayyarbi**G** Low *Pr* HLA-II alleles are associated with lower HIV incidence. The plot shows the summary of a Cox model on the cumulative incidence of HIV, treating HLA-DRB1 *Pr* as a continuous variable (left). HR: hazard ratio. The *p*-values of a two-sided Wald test (explaining the significance of the independent variable) and a log-rank test (explaining model significance) are indicated. Cumulative incidence plots are shown for groups created by median *Pr* (middle), and top and bottom deciles (right). *n* = 197 infected and 233,747 non-infected individuals in the lower-than-median group; *n* = 264 infected and 219,155 non-infected individuals in the higher-than-median group. *n* = 33 infected and 45702 non-infected individuals in the lowest decile group; *n* = 50 infected and 43,357 non-infected individuals in the highest decile group.

The above results suggest that promiscuous HLA-II variants might offer protection against common infectious diseases. To investigate this possibility, we analyzed data from the UK Biobank, a large prospective cohort comprising over 500,000 participants^32^ (Fig. 4A). Our analysis primarily focused on HLA-DRB1, which plays a key role in the immune defense against various infectious diseases^14,33^. We successfully determined HLA-DRB1 promiscuity levels for over 96% of UK Biobank participants with available HLA-genotype data. We then comprehensively reviewed participants’ medical records for evidence of infectious diseases, focusing on those affecting both sexes and most likely caused by common pathogens (see Supplementary Tables 4 and 5 for a list of included ICD10 codes).

We quantified the number of inpatient infectious disease diagnoses for each participant: 76.0% had no recorded infectious diseases, 19.5% had one or two, and 4.5% had three or more (Fig. 4A). We divided participants into five groups based on the quintiles of HLA-DRB1 promiscuity levels. Using logistic regression, we assessed the prevalence of infectious diseases across these varying levels of promiscuity. Our analysis revealed a negative relationship between HLA-DRB1 promiscuity level and susceptibility to severe infectious diseases resulting in hospitalization (Fig. 4B). Specifically, individuals in the highest promiscuity group exhibited a 5.6% lower odds of contracting at least one such infectious disease compared to those in the lowest promiscuity group. This association remained statistically significant after adjusting for genetic principal components (Fig. 4C). Moreover, in another model, *Pr* had the strongest effect on disease risk among HLA-II-associated features, followed by homozygosity^12,34^ with a weaker significant effect and the evolutionary divergence of HLA-DRB1 molecules (HED)^11^ with no significant effect (Supplementary Table 6**)**. The effect of HLA-II *Pr* also remained when controlling for age, sex, and the presence of immunodeficiency (see Supplementary Table 7). To further minimize potential confounding effects from ancestry groups with distinct *Pr* levels, we restricted the analysis to individuals of British ancestry (*n* = 402,632). Importantly, the results remained consistent (Supplementary Fig. 5). Additionally, to account for the influence of HLA alleles associated with common infections, we included protective and risk alleles as covariates, as identified in a previous large-scale GWAS^35^. The effect of promiscuity remained robust (Supplementary Table 8). Finally, we conducted an analysis focusing on diagnoses where the role of the adaptive immune system is unlikely (e.g., accidents, toxicities, etc.). Reassuringly, promiscuity had no effect on the occurrence of these diagnoses (Supplementary Fig. 6).

Next, we examined how HLA-II promiscuity influences the cumulative incidence of the infectious disease categories included in the previous analysis. We incorporated data from both inpatient and outpatient medical records, including general practitioner data. Using Cox proportional hazards models, we analyzed the incidence of each disease group, treating HLA-DRB1 promiscuity level as a continuous variable. After adjusting for multiple hypothesis testing, we identified eight disease groups whose occurrence decreased with increased HLA promiscuity (Fig. 4D). These associations included viral and bacterial skin infections, sexually transmitted diseases (dominated by genital warts), lower respiratory tract infections, and mycoses. To further illustrate the strongest associations, we focused on the four most significant disease groups and compared cumulative incidence between individuals in the top and bottom 10% of HLA-DRB1 promiscuity (Fig. 4E). The strongest association was observed in the “other sexually transmitted diseases” group, predominantly linked to genital warts caused by human papillomavirus (HPV). Individuals in the top 10% of HLA-DRB1 promiscuity level demonstrated over a 45% lower hazard of infection over their lifetime compared to those in the bottom 10% (Fig. 4E). Additionally, this protective effect was significant for viral warts of the skin, also caused by HPV, with a 15.7% reduced hazard. Similar reductions were observed for herpes zoster (14.3% reduced hazard), caused by the varicella-zoster virus, and for local skin and soft tissue infections, primarily bacterial in origin (14.1% reduced hazard) **(**Fig. 4E). Notably all results remained significant when we controlled for the effect of HLA alleles potentially associated with these diseases in multiple Cox models (Supplementary Table 9, see Methods for details).

Our findings suggest that promiscuous HLA-II alleles protect from a variety of infections. In contrast, low HLA-II promiscuity level did not significantly reduce the overall incidence of any common infectious disease. However, this does not imply that low-promiscuity HLA alleles are without benefit. These alleles may play a role in defense against less common pathogens that are markedly distinct from those usually associated with common infections^31^ (Fig. 4F). To explore this, we analyzed the cumulative incidence of HIV infection^36^. HIV-1, a retrovirus in the kingdom *Pararnavirae*, lies on a deeply divergent branch of the RNA virus phylogeny relative to *Orthornavirae* viruses that cause most common human infections^37^, and is therefore expected to have a highly distinct antigenic repertoire^38,39^. Remarkably, the incidence of HIV was significantly lower among individuals with low-promiscuity HLA-DRB1 alleles (Fig. 4G), indicating a protective effect of these alleles against such pathogens. The result remained significant after controlling for HLA alleles associated with HIV (Supplementary Table 9). In summary, our findings suggest distinct roles for HLA-II alleles with low and high *Pr*.

### HLA-II promiscuity level influences response to influenza vaccination in an age-dependent manner

Although we detected an effect of HLA-II promiscuity in eight disease groups, we did not observe an association for several common infections, which could arise from multiple factors. First, susceptibility may be primarily determined by immune mechanisms other than HLA-II presentation. Second, the UK Biobank cohort is highly heterogeneous, leading to substantial variation in exposure to infectious agents. Third, vaccination coverage, timing, and vaccine type differ between individuals. Fourth, both susceptibility to and clinical manifestation of many infections are strongly age-dependent, which may mask the effects of HLA-II promiscuity.

**Fig. 5:**
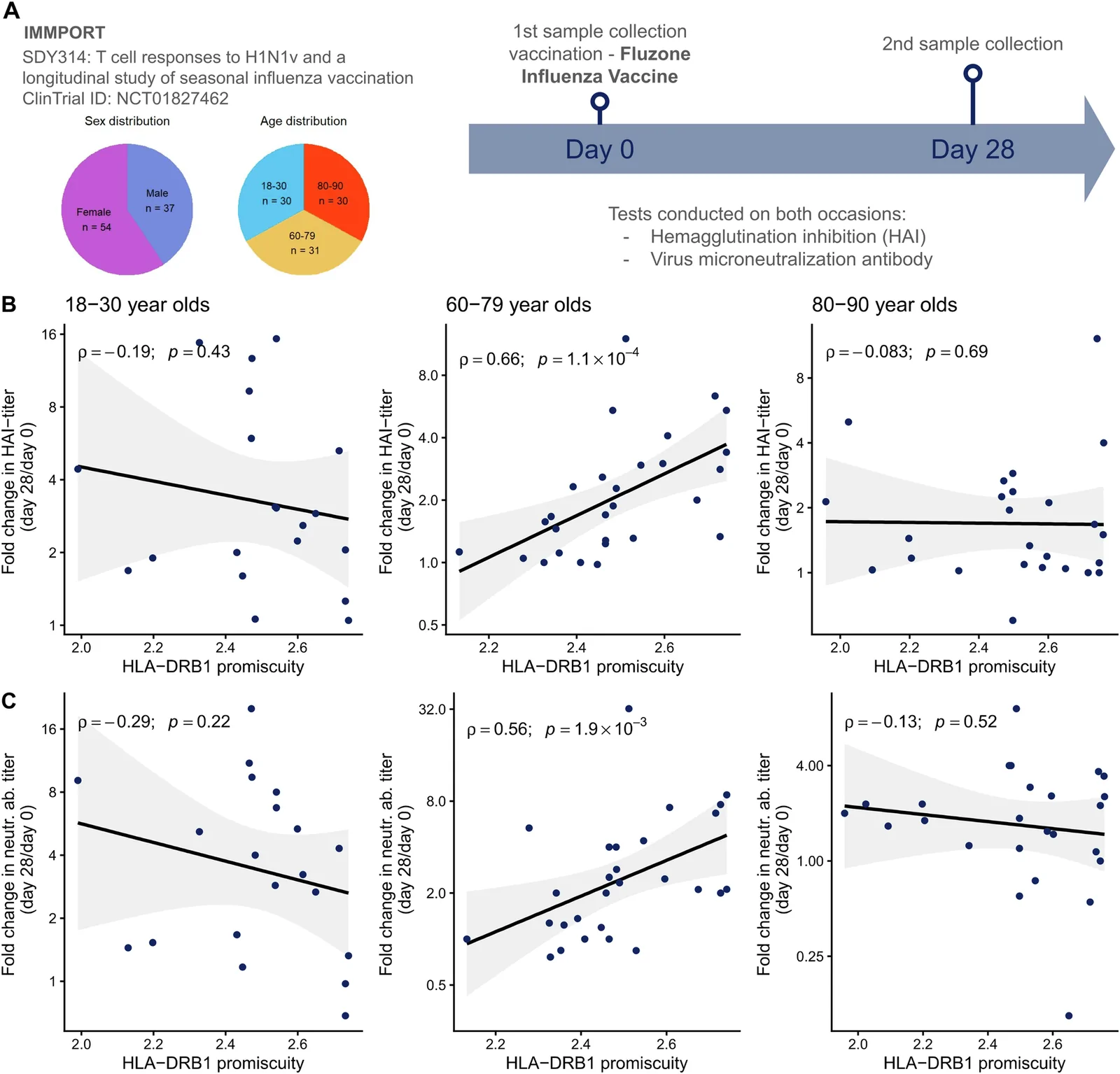
High HLA-DRB1 promiscuity is associated with a stronger response to the Fluzone vaccine in the 60–79-year age group. **A** Overview of the study design and descriptive statistics of the study cohort. **B**,**C** Fold change in hemagglutination inhibition (HAI) titer (**B**) and neutralizing antibody titer (**C**) from day 0 to day 28 as a function of HLA-DRB1 promiscuity in the three age groups. Reported titer values represent the average of the individual strain-specific titers. For each individual, HLA-DRB1 promiscuity was calculated by averaging the values of the two DRB1 alleles. Spearman’s rho values and *p*-values of two-sided correlation tests are indicated, and vertical axes are displayed on a log_2_ scale. The black lines indicate linear regression lines and gray area represents 95% confidence interval.

To reduce these confounding factors, we next focused on an influenza vaccination cohort^40^. In this study, healthy individuals from different age groups received the same inactivated vaccine, Fluzone, according to a standardized protocol (Fig. 5A). This design equalizes antigen exposure (dose, formulation, and timing), thereby removing variability in contact with the virus and in vaccination history. Moreover, because vaccine-induced antibody responses strongly depend on CD4+ T cell help, and thus on HLA-II peptide presentation, a vaccination study provides a particularly suitable setting to assess the impact of HLA-II promiscuity, including its age-dependent effects.

We calculated HLA-DRB1 promiscuity in individuals from three age groups (18–30, 60–79, and 80–90 years) and related it to functional antibody responses to influenza, measured by hemagglutination inhibition (HAI) assays. Vaccine responses were defined as the fold-change in HAI titer from baseline (day 0) to day 28. HLA-II promiscuity showed a strong positive correlation with antibody induction in the 60–79-year group, but not in the 18–30 or 80–90-year groups (Fig. 5B). This pattern was corroborated by the virus neutralization assay, which revealed the same association in the 60–79-year group but not in the other age groups (Fig. 5C).

Together, these data indicate that HLA-II promiscuity is a robust determinant of influenza vaccine responsiveness, specifically in older adults. They further suggest that there is an age window in which broader peptide presentation by HLA-II molecules can be translated into superior antibody responses, before advanced immunosenescence overrides this effect. This age-dependent impact of HLA-II promiscuity has important implications for understanding inter-individual variation in vaccine efficacy and for optimizing vaccination strategies in the elderly.

## Discussion

### HLA-II promiscuity is associated with infectious disease risk

HLA genes are among those under the strongest selective pressure from infectious agents and display the greatest diversity in the human genome^27,41^. In this study, we present evidence of a positive relationship between the breadth of the peptide-binding repertoire of HLA-II variants and antibody-mediated immune responses. Individuals carrying HLA-II variants with a broad peptide-binding repertoire are more likely to produce antibodies that target pathogen-associated proteins and are less likely to experience severe infections from common pathogens, as reflected in their medical records. Additionally, high HLA-II promiscuity was associated with a lower cumulative incidence of a wide range of infections. The consistent protective effects observed for both mucosal and cutaneous HPV indicate that this advantage is not tissue-restricted but extends across distinct epithelial niches. Local bacterial infections of the skin and subcutaneous tissues (e.g., cellulitis) are typically not attributed to host genetics, yet we found that promiscuity also confers protection in these extracellular bacterial settings, suggesting a broad immune benefit. For herpes zoster, previous GWAS have implicated specific alleles in shingles risk^42^, whereas our results show that overall binding breadth, rather than any single allele, predicts protection, underscoring the importance of antigenic diversity in controlling latent virus reactivation. Similarly, candidiasis—a hallmark of impaired T-cell immunity—highlights how presentation breadth shapes susceptibility to opportunistic pathogens. Pneumonia and other lower respiratory tract infections remain among the leading causes of infectious mortality worldwide^1^, and the observation that HLA promiscuity is protective in this context further broadens its clinical relevance. These findings support the hypothesis that promiscuous HLA molecules provide protection against frequently encountered pathogens. Moreover, high HLA-II promiscuity was associated with stronger influenza vaccine responses among individuals aged 60–79 years. Notably, a recent study reported age-related differences in the antibody repertoire targeting influenza A virus—including reduced reactivity to rapidly evolving epitopes and increased reactivity to conserved epitopes^43^.

In an earlier work, we have shown that the most promiscuous HLA-II alleles are rare in certain human populations, suggesting that these alleles are not strongly favored by natural selection in these regions^14^. Why might this be the case? One possibility is that high promiscuity may be less effective against evolutionarily recent, highly virulent pathogens^31^. In such scenarios, presenting a specific epitope could offer a more efficient immune response, while high promiscuity, with its broader but less targeted approach, might be less advantageous due to reduced specificity. Notably, we found that the low-promiscuity HLA-II variants provide protection against HIV, which is in line with previous results on HLA-I^44^. While this result suggests potentially distinct roles for HLA-II alleles with high and low promiscuity, the finding is limited by the focus on a single pathogen and therefore warrants further systematic investigation across less frequent and evolutionarily novel pathogens.

### Study implications, potential impact, and limitations

The level of HLA-II promiscuity serves as a potential biomarker for susceptibility to various infectious diseases, as it captures the overall peptide-binding characteristics rather than focusing on individual alleles or allele groups in isolation. While our data demonstrate the potential of HLA-II promiscuity to predict influenza vaccine responsiveness, the presence of generalist or specialist HLA-II molecules could likewise have a major impact on responses to other vaccines, a possibility that warrants further investigation. Our previous work showed that HLA-I promiscuity strongly influences the efficacy of immune checkpoint blockade therapy^15^. Building on these findings, examining HLA-II promiscuity may likewise reveal an important role in shaping antitumor immune responses. Promiscuous HLA-II molecules may present a larger fraction of self-antigens, which in turn could increase susceptibility to autoimmune diseases. On the other hand, broad HLA presentation of self-peptides could also enhance clonal deletion of self-reactive T cells in the thymus and thereby reduce autoimmune risk^45^. Disentangling these opposing effects will require future studies.

In our study, we aimed to minimize selection bias and maximize generalizability across pathogens and HLA-II alleles. We based our analyses on one of the most comprehensive immunopeptidomics datasets currently available, applying a non-selective inclusion of HLA alleles and a large, high-quality antibody dataset encompassing pathogens, non-pathogens, and common infections. In parallel, we leveraged UK Biobank at population scale without focusing on a specific disease or certain alleles, thereby ensuring that our estimates of HLA-II promiscuity are not driven by a few highly studied conditions or population-specific allelic distributions. Nevertheless, our study has several limitations.

As outlined elsewhere^46^, PhIP-seq is intrinsically limited by peptide length, which captures linear but may miss conformational epitopes that require folding of larger protein regions. The proportion of linear versus conformational epitopes recognized by human antibodies is unknown and difficult to measure; however, even if our approach detected only 10% of antibody–antigen interactions against bacteria, the tens of thousands of proteins in our library would still provide a throughput exceeding current ELISA or peptide array methods by about an order of magnitude. We have shown that phage-displayed peptides can reconstitute expected protein functions requiring at least secondary structure^46^. Furthermore, our work^47^ and that of others^35,48,49^ using full-length proteins suggest similar HLA-II–dependent mechanisms in the selection and maturation of B cells targeting conformational epitopes.

A limitation of our *Pr* estimates is that they are derived from peptide lists assembled from previously published multiallelic HLA-II immunopeptidomics datasets in which peptides were assigned to individual alleles using NetMHCIIpan (either in the original studies or retrospectively). As a result, the inferred diversity profiles for each HLA-II allele—and consequently the *Pr* values—depend on the performance and potential biases of this prediction-based allele assignment. Future work should validate *Pr* estimates using monoallelic data. Furthermore, some HLA-II haplotypes contain not only DRB1 but also DRB3, DRB4, and DRB5 genes. The peptide-binding repertoires of these additional genes could potentially be incorporated into peptide-binding repertoire calculations in future studies.

Although our study primarily focused on the humoral immune response to pathogens, it is important to recognize that cellular, CD8 + T-cell-mediated immune responses also rely on the activation of CD4+ T helper cells by peptides presented through HLA-II molecules. Thus, the patterns observed in the clinical data may also be influenced by non-humoral immune mechanisms, warranting further investigation.

## Methods

UK Biobank and LifeLines DEEP were both conducted with ethics committee approval and written informed consent from participants. UK Biobank is governed under a dedicated Ethics and Governance Framework with independent ethical oversight, while LifeLines DEEP was approved by the ethics committee of the University Medical Center Groningen as a subcohort of the broader LifeLines study, which follows the principles of the Declaration of Helsinki (reference numbers M12.113965 and 2008/338). This research was done under UKB application No. 272827.

### Statistics and reproducibility

This study used previously generated immunopeptidomics, antibody-profiling, vaccination-response, and population-scale electronic health record datasets. No statistical method was used to predetermine sample size. Sample sizes were determined by the availability of eligible participants or samples with the required HLA genotype, antibody, vaccination-response, or clinical phenotype data in each source dataset. Data were excluded only according to predefined analytical criteria, including missing HLA or promiscuity values, insufficient allele or peptide coverage, or filtering thresholds specified for individual analyses.

Statistical analyses included Spearman correlation tests, Fisher’s exact tests, logistic regression, Cox proportional hazards models, and mixed-effects linear models, as appropriate for each analysis. Regression models included relevant covariates, such as HLA group, age, sex, cohort, sequencing plate, genetic principal components, immunodeficiency status, and infection-associated HLA alleles where applicable. Multiple testing was controlled using the Benjamini–Hochberg false discovery rate procedure.

The experiments were not randomized. The investigators were not blinded. Reproducibility was supported using publicly described datasets and standardized computational workflows. Data are available from the UK Biobank, LifeLines DEEP, and ImmPort, subject to their respective access restrictions, and the code used for calculating HLA promiscuity and generating the main analyses is available through the GitHub repositories described in the Code availability section.

### Determining the promiscuity of HLA-II molecules

We calculated the promiscuity of HLA-II molecules using the same methodology previously employed for assessing HLA-I promiscuity^15^. Specifically, we utilized a large immunopeptidomic dataset comprising 627,013 pairs of peptides and HLA-II molecules^19^. Our analysis focused on the binding core region, which is essential for interaction with the peptide-binding groove and determines the peptide’s binding affinity^19^. Notably, the dataset included detailed information on the core region for each peptide-HLA pair. We specifically focused on data related to HLA-DP, HLA-DQ, and HLA-DR molecules.

Given that the alpha chain of HLA-DR molecules is invariant^19^, we included only those molecules with at least two-field resolution data on the beta chain, encoded by the HLA-DRB1 gene. For HLA-DP and HLA-DQ molecules, where both the alpha and beta chains are variable, we included molecules with at least two-field resolution data for both chains, encoded by the HLA-DPA1 and HLA-DQA1 genes for the alpha chains, and the HLA-DPB1 and DQB1 genes for the beta chains, respectively. We retained only one instance of each core region sequence that appeared multiple times in the dataset in pair with the same HLA molecule.

Then, for each HLA molecule with at least 100 remaining sequences, we took the amino acid prevalence at every position of the core region of the bound peptides. For each position, we calculated the Kullback-Leibler divergence between the observed distribution of amino acids and the natural amino acid frequencies present in human proteins:

$${D}_{{KL}}(y)={\sum}_{x\in \chi }P\left(x,y\right)\log \left(\frac{P\left(x,y\right)}{Q\left(x\right)}\right)$$

where *P(x,y)* is the frequency of amino acid *x* at position *y*, *Q(x)* is the frequency of amino acid *x* found in human proteins and *χ* is the complete set of the twenty amino acids. A constant value of 10^-7^ was added to every amino acid value when the prevalence of any amino acid was zero at the position. We then averaged the position-specific values, yielding *D*_*KL*_ for the entire core region. After normalizing the calculated *D*_*KL*_ values (see ref. ^15^ for details), we took their reciprocal value reflecting the promiscuity of each HLA molecule.

Notably, our promiscuity metric, which is computed from amino acid distributions across all positions of 9-residue binding cores, inherently assigns greater weight to anchor residues: their structurally constrained, restricted amino acid usage produces higher Kullback–Leibler divergence values and thus disproportionately influences the overall promiscuity score. Consistent with this, promiscuity values based on all positions are highly correlated with those calculated from anchor positions only (Spearman’s rho = 0.92, *p*-value of two-sided correlation test <2.2 × 10^-16^).

### HLA-binding prediction, validation of the promiscuity measure, amino acid specificity of HLA alleles

Protein sequences from the Immune Epitope Database^25^ and the Virulence Factor Database^26^ were decomposed to overlapping 15-mer sequences. The binding of each sequence was predicted to each of the 69 examined HLA-II molecules using NetMHCIIpan-4.3^24^. As discussed previously^15^, the rank percentile value is not suitable for assessing the size of the peptide-binding repertoire, as it standardizes the repertoire size across all HLA-II molecules. Therefore, we utilized the EL score to represent the peptide-binding characteristics of HLA-II molecules more accurately.

Immunopeptidomics data for the capsid protein of AAV strains were obtained from the original publication^22^. For each identified peptide, the authors provided details on the HLA allele predicted to present the peptide and the associated binding core region. We compiled the unique core sequences assigned to each allele and calculated pairwise BLOSUM62 similarity scores using the parSeqSim function from the protr 1.7-5 R library^50^, applying global alignment with default parameters. We focused on alleles with more than 25 identified core sequences and defined sequence diversity in two ways: (i) the fraction of sequence pairs with very low similarity (calculated similarity values less than zero), and (ii) the median similarity value.

The immunopeptidomics data for the spike protein of SARS-CoV-2 were retrieved from the original study^23^. Since the data did not include information on the HLA alleles presenting the peptides, we predicted them as follows. For each individual, we used the NetMHCIIpan-4.0 algorithm^51^ to predict the binding of each identified peptide to every HLA-II molecule expressed by that individual. Each peptide was assigned to the HLA-II molecule with the lowest predicted binding rankEL value. Peptides with a minimum rankEL value above 5 were excluded from the analysis. For each HLA-II allele, we collected all core sequences as predicted by the NetMHCIIpan-4.0 algorithm and assessed sequence diversity for alleles with at least three identified core sequences, using the same approach as for the AAV dataset.

We created sequence motifs for each allele using the same unique peptide core region sequences that were used for calculating promiscuity. Motifs were created based on information content matrices using the universalmotif 1.28.0 R library^52^.

### Determining the relationship between HLA promiscuity and PhIP-seq data

We used data on the relationship between imputed HLA genotypes and the presence of 2815 antibodies from ref. ^4^. Briefly, genetic data for 1175 volunteers from the LifeLines DEEP population cohort^4,53^ and 315 volunteers from the 1000IBD IBD cohort^16^ with available PhIP-seq profiles were used in the original study. HLA genotypes were imputed from genome-wide SNP data as described in the original paper^4^. A logistic regression model was fitted for each HLA allele, amino acid and SNP located in the HLA locus:

$${AntibodyBoundPeptide} \sim {HLAimp}+{sex}+{age}+{plateID},$$

where the dependent variable indicates the presence or absence of an antibody specific to a given peptide. *HLAimp* represents the SNP2HLA category (HLA allele, amino acid or SNP), and *plateID* refers to the sequencing plate used. In the IBD cohort, models were further adjusted for disease subtype (Crohn’s disease or ulcerative colitis). Overall meta-analysis summary statistics were generated using PLINK^54^, and meta-p-values were corrected using the Benjamini-Hochberg method^55^.

Only those HLA-II alleles (*n* = 23) were included in the analysis for which both promiscuity values were available and that were tested for associations with antibodies. For each HLA-II allele, we filtered associations with a false discovery rate (FDR) below 0.05. Associations were classified as positive or negative based on the direction of the beta values. A value of one was added to the count of positive and negative associations to avoid division by zero. For HLA-DP and HLA-DQ molecules, we assigned a positive or negative association to an αβ heterodimer only when both the α- and β-chains showed a significant association with the same epitope in the same direction (i.e., both chains were either positively or negatively associated with the presence of the same antibody). Organisms were categorized as pathogens or non-pathogens according to the GIDEON database^14,30^ (Supplementary Table 2). Notably, we also considered *Clostridium botulinum*, *Haemophilus influenzae* and *Mycoplasma pneumoniae* as pathogens, even though they were not explicitly mentioned in the database.

In the individual-level analysis, HLA promiscuity for DP and DQ was calculated for each individual by determining all possible alpha-beta chain combinations and averaging their promiscuity values. Since the alpha chain of HLA-DR is invariable, promiscuity for DRB1 was calculated based solely on the beta chain molecules. HLA-DQ was excluded from the analysis due to insufficient coverage (30.8%) of individuals within the cohort. Individuals missing HLA-DP or DRB1 values were also excluded, resulting in a final dataset of 916 individuals. Overall, HLA-II promiscuity was computed by summing DP and DRB1 promiscuity values for each individual. Then, we counted the number of antibody-bound peptides belonging to pathogens and non-pathogens, which were then used as the dependent variables in mixed-effects linear models. HLA-II promiscuity served as the independent variable, with individual age and sex included as fixed effects. The dataset (LifeLines DEEP or IBD) was incorporated as a random effect, along with a nested random effect for sequencing plate IDs, which varied by cohort.

### Analysis of individuals in the UK Biobank cohort

We analyzed data from 502,551 participants in the UK Biobank cohort^32^. HLA-DRB1 promiscuity for each individual was calculated by averaging the allele-specific values of the beta chains encoded by the HLA-DRB1 gene. Individuals with missing values were excluded from the analysis, resulting in a final dataset of 453,545 participants. Notably, in some analyses, the number of cases and non-cases may not sum to the total sample size because of missing outcome or covariate data.

The analysis was conducted using two datasets: the hospital admission dataset (Field 41270) and the first disease occurrence dataset (Field 1712). Medical records were filtered for ICD-10 codes corresponding to common infectious diseases (ICD A04, A08, A09, A40, A41, A51, A52, A53, A54, A56, A58, A59, A60, A63, A64, B00, B01, B02, B05, B06, B07, B08, B09, B25, B27, B35, B36, B37, J00, J01, J02, J03, J04, J05, J06, J09, J10, J11, J12, J13, J14, J15, J16, J17, J18, J20, J21, J22, J36, J86, L03, L08, N39, U07). Participants were stratified into five HLA-DRB1 promiscuity groups based on quintile values. Data on genetic principal components, sex, and age were obtained from Fields 22009, 31, 34, and 52. Participants with potential immunodeficiency were identified using specific ICD-10 codes (B20, C81-C97, D81-D84, E10, E11, G35, G61, G70, H20, K50, K51, L10, L12, L40, M05, M06, M08, M30-M36, M45). To account for the effect of HLA alleles potentially associated with infectious diseases, we utilized the PheWAS database^56^. This database provides information on the associations of HLA alleles with the occurrence of diagnoses in the UK Biobank. For each infection, we included all associations involving two-field-resolution alleles with a p-value < 0.01. Since the PheWAS database reports associations for Phecode diagnoses, we mapped each disease to its corresponding ICD-10 diagnoses as follows: 078-A63, 078-B07, 686-L08, 053-B02, 112-B37, 681-L03, 519.8-J22. Additionally, for HIV infection (Phecode: 071), we incorporated further well-known alleles identified from the literature^13^. The evolutionary divergence of HLA-DRB1 was determined using the calculated HLA Evolutionary Divergence (cHED) site of the Transplants’ Open Registry (TxOR) app^57^.

### Reporting summary

Further information on research design is available in the Nature Portfolio Reporting Summary linked to this article.

## Supplementary information

## Source data

## Data Availability

The datasets analyzed in this study are available from the UK Biobank and the LifeLines DEEP cohort but are subject to access restrictions. Data from the UK Biobank are available upon application and approval via their Access Management System (https://www.ukbiobank.ac.uk/enable-your-research/apply-for-access), which requires a detailed research proposal and agreement to data use terms. Similarly, access to the LifeLines DEEP data can be requested through a formal application process (https://www.lifelines.nl/researcher), including submission of a research plan and compliance with ethical and data privacy regulations. Data are available only for approved academic and health-related research purposes. Data from the Fluzone vaccine trial are available in the ImmPort database (study ID: SDY314) upon registration. Source data are provided with this paper.

## Code availability

The code used for calculating HLA promiscuity is publicly available on GitHub (https://github.com/matemanc/hla_promiscuity)^58^. Other code for generating results is also available on GitHub (https://github.com/matemanc/promisc_ab_publ)^59^. Scripts used for the analysis of the UK Biobank cohort are hosted within the UK Biobank Research Analysis Platform and are accessible to approved researchers following a successful application to the UK Biobank (https://www.ukbiobank.ac.uk/enable-your-research/apply-for-access).

## References

[1] Dattani, S., Spooner, F., Ritchie, H. & Roser, M. Causes of death. *Our World in Data* https://ourworldindata.org/causes-of-death (OurWorldinData, 2023).

[2] Adler, L. N. et al. The other function: class II-restricted antigen presentation by B cells. *Front. Immunol*. **8**, 319 (2017). 10.3389/fimmu.2017.00319 PMC5362600 28386257

[3] Barker, D. J. et al. The IPD-IMGT/HLA database. *Nucleic Acids Res.* **51**, D1053–D1060 (2023). 36350643 10.1093/nar/gkac1011 PMC9825470

[4] Andreu-Sánchez, S. et al. Phage display sequencing reveals that genetic, environmental, and intrinsic factors influence variation of human antibody epitope repertoire. *Immunity* **56**, 1376–1392.e8 (2023). 37164013 10.1016/j.immuni.2023.04.003 PMC12166656

[5] Augusto, D. G. et al. A common allele of HLA is associated with asymptomatic SARS-CoV-2 infection. *Nature* **620**, 128–136 (2023). 37468623 10.1038/s41586-023-06331-x PMC10396966

[6] Medhasi, S. & Chantratita, N. Human leukocyte antigen (HLA) system: genetics and association with bacterial and viral infections. *J. Immunol. Res.* **2022**, 9710376 (2022). 35664353 10.1155/2022/9710376 PMC9162874

[7] Blackwell, J. M., Jamieson, S. E. & Burgner, D. HLA and infectious diseases. *Clin. Microbiol. Rev.* **22**, 370–385 (2009). 19366919 10.1128/CMR.00048-08 PMC2668228

[8] Marchal, A. et al. Lack of association between classical HLA genes and asymptomatic SARS-CoV-2 infection. *HGGADVANCE* **5**, 100300 (2024). 10.1016/j.xhgg.2024.100300 PMC11215417 38678364

[9] Kennedy, A. E., Ozbek, U. & Dorak, M. T. What has GWAS done for HLA and disease associations? *Int. J. Immunogenet.* **44**, 195–211 (2017). 28877428 10.1111/iji.12332

[10] Sanchez-Mazas, A. A review of HLA allele and SNP associations with highly prevalent infectious diseases in human populations. *Swiss Med. Wkly.* **150**, w20214–w20214 (2020). 32297957 10.4414/smw.2020.20214

[11] Chowell, D. et al. Evolutionary divergence of HLA class I genotype impacts efficacy of cancer immunotherapy. *Nat. Med.* **25**, 1715–1720 (2019). 31700181 10.1038/s41591-019-0639-4 PMC7938381

[12] Tang, J. et al. HLA class I homozygosity accelerates disease progression in human immunodeficiency virus type 1 infection. *AIDS Res Hum. Retroviruses* **15**, 317–324 (1999). 10082114 10.1089/088922299311277

[13] Viard, M. et al. Impact of HLA class I functional divergence on HIV control. *Science* **383**, 319–325 (2024). 38236978 10.1126/science.adk0777 PMC11395297

[14] Manczinger, M. et al. Pathogen diversity drives the evolution of generalist MHC-II alleles in human populations. *PLOS Biol.* **17**, e3000131 (2019). 30703088 10.1371/journal.pbio.3000131 PMC6372212

[15] Manczinger, M. et al. Negative trade-off between neoantigen repertoire breadth and the specificity of HLA-I molecules shapes antitumor immunity. *Nat. Cancer* **2**, 950–961 (2021). 35121862 10.1038/s43018-021-00226-4

[16] Bourgonje, A. R. et al. Phage-display immunoprecipitation sequencing of the antibody epitope repertoire in inflammatory bowel disease reveals distinct antibody signatures. *Immunity* **56**, 1393–1409.e6 (2023). 37164015 10.1016/j.immuni.2023.04.017

[17] Marty, R. et al. MHC-I genotype restricts the oncogenic mutational landscape. *Cell* **171**, 1272–1283.e15 (2017). 29107334 10.1016/j.cell.2017.09.050 PMC5711564

[18] Chowell, D. et al. Patient HLA class I genotype influences cancer response to checkpoint blockade immunotherapy. *Science* **359**, 582–587 (2018). 29217585 10.1126/science.aao4572 PMC6057471

[19] Racle, J. et al. Machine learning predictions of MHC-II specificities reveal alternative binding mode of class II epitopes. *Immunity* **56**, 1359–1375.e13 (2023). 37023751 10.1016/j.immuni.2023.03.009

[20] Greenbaum, J. et al. Functional classification of class II human leukocyte antigen (HLA) molecules reveals seven different supertypes and a surprising degree of repertoire sharing across supertypes. *Immunogenetics* **63**, 325–335 (2011). 21305276 10.1007/s00251-011-0513-0 PMC3626422

[21] Sanchez-Mazas, A. et al. The most frequent HLA alleles around the world: a fundamental synopsis. *Best. Pract. Res. Clin. Haematol.* **37**, 101559 (2024). 39098805 10.1016/j.beha.2024.101559

[22] Brito-Sierra, C. A., Lannan, M. B., Siegel, R. W. & Malherbe, L. P. The HLA class II immunopeptidomes of AAV capsids proteins. *Front. Immunol*. **13**, 1067399 (2022). 10.3389/fimmu.2022.1067399 PMC9807805 36605211

[23] Knierman, M. D. et al. The human leukocyte antigen class II immunopeptidome of the SARS-CoV-2 Spike glycoprotein. *Cell Rep.* **33**, 108454 (2020). 33220791 10.1016/j.celrep.2020.108454 PMC7664343

[24] Nilsson, J. B. et al. Accurate prediction of HLA class II antigen presentation across all loci using tailored data acquisition and refined machine learning. *Sci. Adv.* **9**, eadj6367 (2023). 38000035 10.1126/sciadv.adj6367 PMC10672173

[25] Vita, R. et al. The immune epitope database (IEDB): 2024 update. *Nucleic Acids Res* **53**, D436–D443 (2025). 39558162 10.1093/nar/gkae1092 PMC11701597

[26] Zhou, S., Liu, B., Zheng, D., Chen, L. & Yang, J. VFDB 2025: an integrated resource for exploring anti-virulence compounds. *Nucleic Acids Res.* **53**, D871–D877 (2025). 39470738 10.1093/nar/gkae968 PMC11701737

[27] Radwan, J., Babik, W., Kaufman, J., Lenz, T. L. & Winternitz, J. Advances in the evolutionary understanding of MHC polymorphism. *Trends Genet.* **36**, 298–311 (2020). 32044115 10.1016/j.tig.2020.01.008

[28] Rantz, L. A., Randall, E. & Rantz, H. H. Antistreptolysin O: a study of this antibody in health and in hemolytic streptococcus respiratory disease in man. *Am. J. Med* **5**, 3–23 (1948). 18867849 10.1016/0002-9343(48)90003-5

[29] Mortensen, R. et al. Identifying protective Streptococcus pyogenes vaccine antigens recognized by both B and T cells in human adults and children. *Sci. Rep.* **6**, 22030 (2016). 26911649 10.1038/srep22030 PMC4766568

[30] GIDEON® Global Infectious Diseases and Epidemiology Online Network. (Gideon, 2018)

[31] Kaufman, J. Generalists and specialists: a new view of how MHC class I molecules fight infectious pathogens. *Trends Immunol.* **39**, 367–379 (2018). 29396014 10.1016/j.it.2018.01.001 PMC5929564

[32] Sudlow, C. et al. UK Biobank: an open access resource for identifying the causes of a wide range of complex diseases of middle and old age. *PLoS Med.* **12**, e1001779 (2015). 25826379 10.1371/journal.pmed.1001779 PMC4380465

[33] Satta, Y., O’hUigin, C., Takahata, N. & Klein, J. Intensity of natural selection at the major histocompatibility complex loci. *Proc. Natl. Acad. Sci. USA* **91**, 7184–7188 (1994). 8041766 10.1073/pnas.91.15.7184 PMC44363

[34] Khan, T. et al. Human leukocyte antigen class II gene diversity tunes antibody repertoires to common pathogens. *Front. Immunol.* **13**, 856497 (2022). 36003377 10.3389/fimmu.2022.856497 PMC9393332

[35] Tian, C. et al. Genome-wide association and HLA region fine-mapping studies identify susceptibility loci for multiple common infections. *Nat. Commun.* **8**, 599 (2017). 28928442 10.1038/s41467-017-00257-5 PMC5605711

[36] Morens, D. M. & Fauci, A. S. Emerging infectious diseases: threats to human health and global stability. *PLOS Pathog.* **9**, e1003467 (2013). 23853589 10.1371/journal.ppat.1003467 PMC3701702

[37] Černý, J. et al. Evolution of tertiary structure of viral RNA dependent polymerases. *PLoS ONE* **9**, e96070 (2014). 24816789 10.1371/journal.pone.0096070 PMC4015915

[38] Kwong, P. D. et al. Structure of an HIV gp120 envelope glycoprotein in complex with the CD4 receptor and a neutralizing human antibody. *Nature* **393**, 648–659 (1998). 9641677 10.1038/31405 PMC5629912

[39] Lipsitch, M. & O’Hagan, J. J. Patterns of antigenic diversity and the mechanisms that maintain them. *J. R. Soc. Interface* **4**, 787–802 (2007). 17426010 10.1098/rsif.2007.0229 PMC2394542

[40] NIAID Data Discovery Portal | T cell responses to H1N1v and a longitudinal study of seasonal influenza vaccination (TIV) SLVP015 2008 (See companion studies SDY315 2012 / SDY312 2009 / SDY311 2010 / SDY112 2011). *NIAID Data Ecosystem Discovery Portal* https://data.niaid.nih.gov/resources?id=sdy314.

[41] Quintana-Murci, L. Human immunology through the lens of evolutionary genetics. *Cell* **177**, 184–199 (2019). 30901539 10.1016/j.cell.2019.02.033

[42] Crosslin, D. R. et al. Genetic variation in the HLA region is associated with susceptibility to herpes zoster. *Genes Immun.* **16**, 1–7 (2015). 25297839 10.1038/gene.2014.51 PMC4308645

[43] Olin, A. et al. Demographic and genetic factors shape the epitope specificity of the human antibody repertoire against viruses. *Nat. Immunol.* **27**, 600–612 (2026). 41699059 10.1038/s41590-026-02432-7 PMC12956577

[44] Košmrlj, A. et al. Effects of thymic selection of the T-cell repertoire on HLA class I-associated control of HIV infection. *Nature* **465**, 350–354 (2010). 20445539 10.1038/nature08997 PMC3098720

[45] Marx, A. et al. Thymus and autoimmunity. *Semin Immunopathol.* **43**, 45–64 (2021). 33537838 10.1007/s00281-021-00842-3 PMC7925479

[46] Vogl, T. et al. Population-wide diversity and stability of serum antibody epitope repertoires against human microbiota. *Nat. Med.* **27**, 1442–1450 (2021). 34282338 10.1038/s41591-021-01409-3

[47] Innocenti, G. et al. Associations between HLA-II variation and antibody specificity are predicted by antigen properties. *Genome Med.* **17**, 65 (2025). 40457459 10.1186/s13073-025-01486-w PMC12131526

[48] Hammer, C. et al. Amino acid variation in HLA class II proteins is a major determinant of humoral response to common viruses. *Am. J. Hum. Genet.* **97**, 738–743 (2015). 26456283 10.1016/j.ajhg.2015.09.008 PMC4667104

[49] Scepanovic, P. et al. Human genetic variants and age are the strongest predictors of humoral immune responses to common pathogens and vaccines. *Genome Med.* **10**, 59 (2018). 30053915 10.1186/s13073-018-0568-8 PMC6063007

[50] Xiao, N., Cao, D.-S., Zhu, M.-F. & Xu, Q.-S. protr/ProtrWeb: R package and web server for generating various numerical representation schemes of protein sequences. *Bioinformatics* **31**, 1857–1859 (2015). 25619996 10.1093/bioinformatics/btv042

[51] Reynisson, B., Alvarez, B., Paul, S., Peters, B. & Nielsen, M. NetMHCpan-4.1 and NetMHCIIpan-4.0: improved predictions of MHC antigen presentation by concurrent motif deconvolution and integration of MS MHC eluted ligand data. *Nucleic Acids Res.* **48**, W449–W454 (2020). 32406916 10.1093/nar/gkaa379 PMC7319546

[52] Tremblay, B. J.-M. Universalmotif: an R package for biological motif analysis. *J. Open Source Softw.* **9**, 7012 (2024).

[53] Tigchelaar, E. F. et al. Cohort profile: lifeLines DEEP, a prospective, general population cohort study in the northern Netherlands: study design and baseline characteristics. *BMJ Open* **5**, e006772 (2015). 26319774 10.1136/bmjopen-2014-006772 PMC4554905

[54] Purcell, S. et al. PLINK: a tool set for whole-genome association and population-based linkage analyses. *Am. J. Hum. Genet* **81**, 559–575 (2007). 17701901 10.1086/519795 PMC1950838

[55] Benjamini, Y. & Hochberg, Y. Controlling the false discovery rate: a practical and powerful approach to multiple testing. *J. R. Stat. Soc. Ser. B* **57**, 289–300 (1995).

[56] Karnes, J. H. et al. Phenome-wide scanning identifies multiple diseases and disease severity phenotypes associated with HLA variants. *Sci. Transl. Med.* **9**, eaai8708 (2017). 28490672 10.1126/scitranslmed.aai8708 PMC5563969

[57] Transplants’ Open Registry (TxOR), https://txor.shinyapps.io/ched/.

[58] Manczinger, M. Negative trade-off between neoantigen repertoire breadth and the specificity of HLA-I molecules shapes antitumor immunity, https://github.com/matemanc/hla_promiscuity, (2021) 10.1038/s43018-021-00226-4 35121862

[59] Magyari, B., Fülöp, A. T. & Manczinger, M. HLA-II peptide-binding diversity shapes humoral immune responses and susceptibility to infections, https://github.com/matemanc/promisc_ab_publ, (2026)10.1038/s41467-026-76184-142686785

