# Supplementary material for: HLA-II peptide-binding diversity shapes humoral immune responses and susceptibility to infections
Source: Nat Commun. 2026 Aug 5;17:9422. doi: 10.1038/s41467-026-76184-1 (PMC13538663; doi:10.1038/s41467-026-76184-1)
Supplement: Supplementary file 1 [file 41467_2026_76184_MOESM1_ESM.pdf]

Supplementary Information

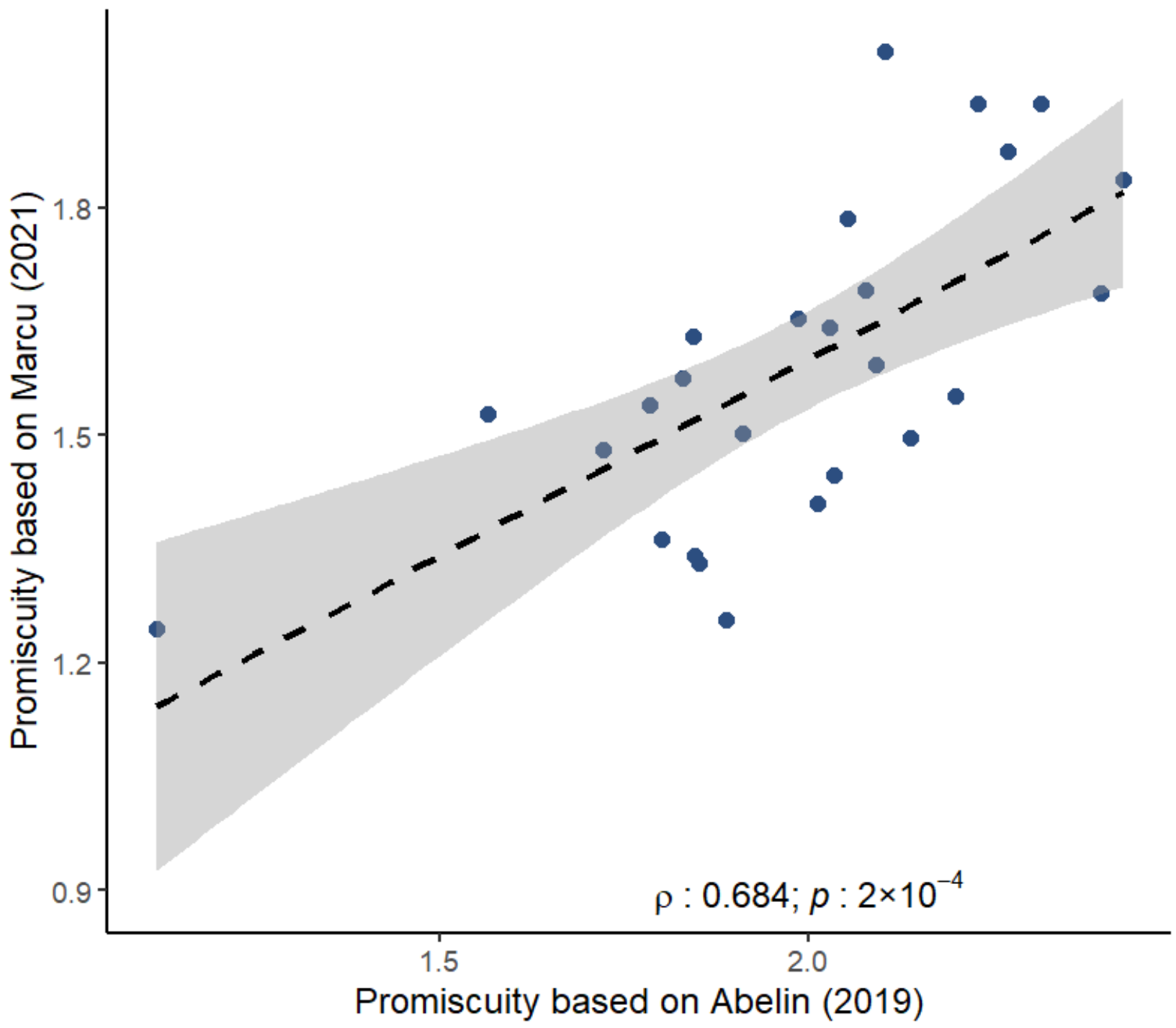

Supplementary Fig. 1. HLA-II promiscuity values remain consistent when calculated using different immunopeptidomics datasets. *Pr* values were calculated using two subsets of ref (16) Spearman's rho and two-sided p value of a correlation test is indicated. The dashed line and the grey-shaded area indicate linear regression line with 95% confidence interval.

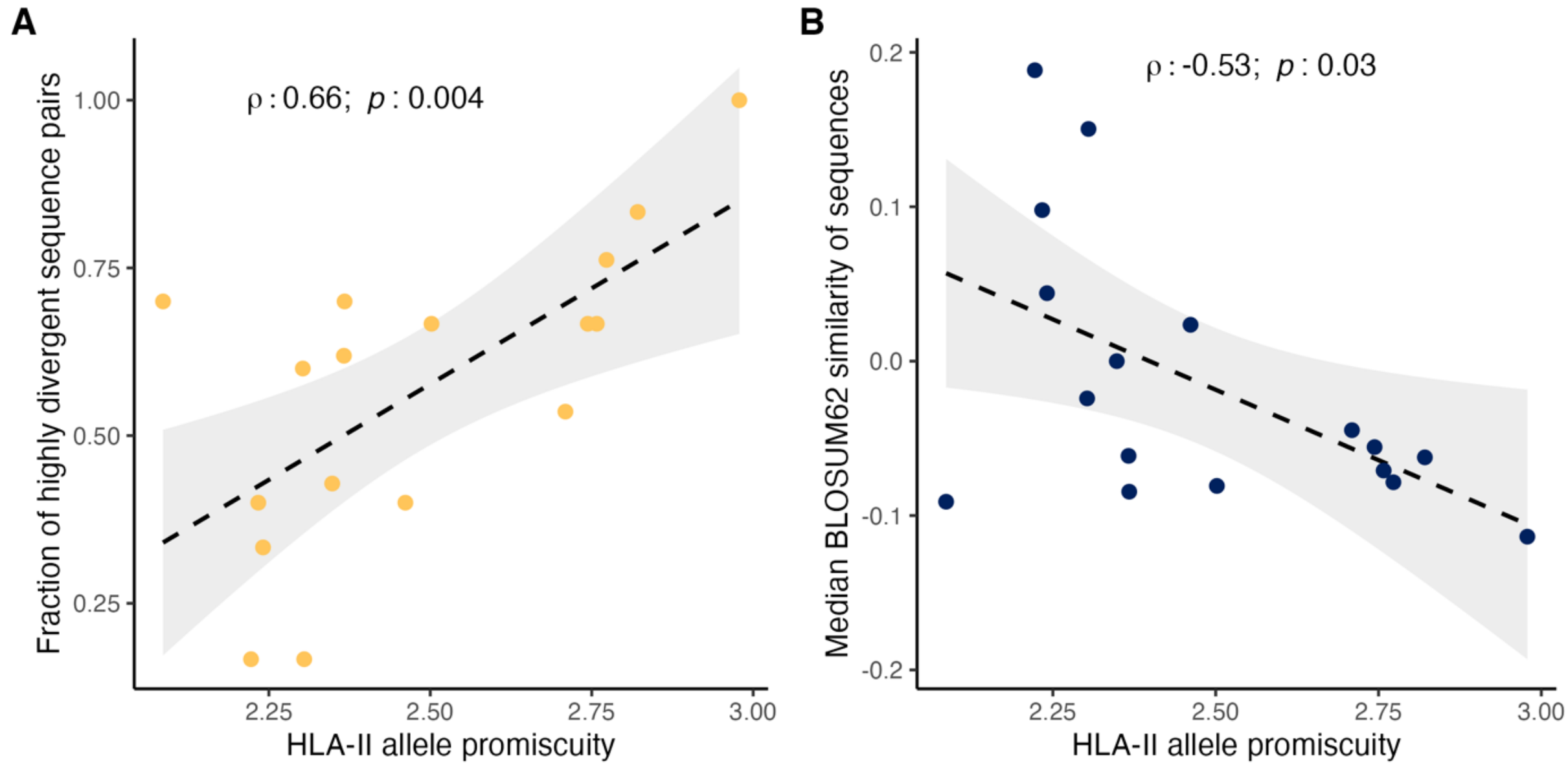

Supplementary Fig. 2. HLA-II promiscuity determines the diversity of presented SARS-CoV-2 spike peptides. For each HLA-II variant, the fraction of highly divergent sequence pairs (BLOSUM62 sequence similarity value < 0) (A) and the median of BLOSUM62 similarity values (B) between presented peptides were calculated. Spearman's rho and two-sided p values of correlation tests are indicated.

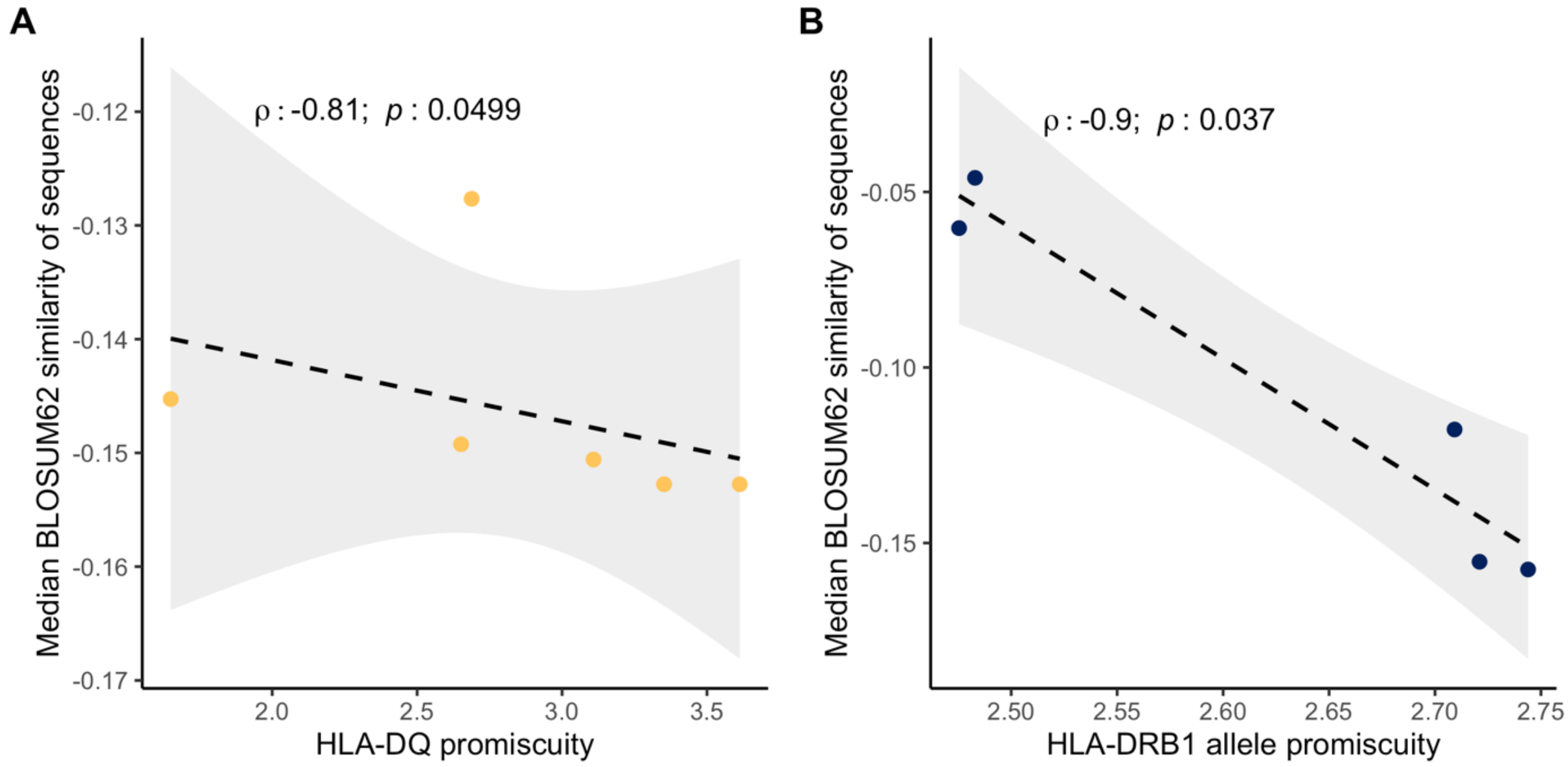

Supplementary Fig. 3. HLA-II promiscuity determines the median BLOSUM62 similarity of AAV capsid sequences. The median BLOSUM62 similarity of presented sequences by HLA-DQ (A) and HLA-DRB1 (B) is shown as a function of allele promiscuity. Spearman rho and two-sided p values of correlation tests are indicated.

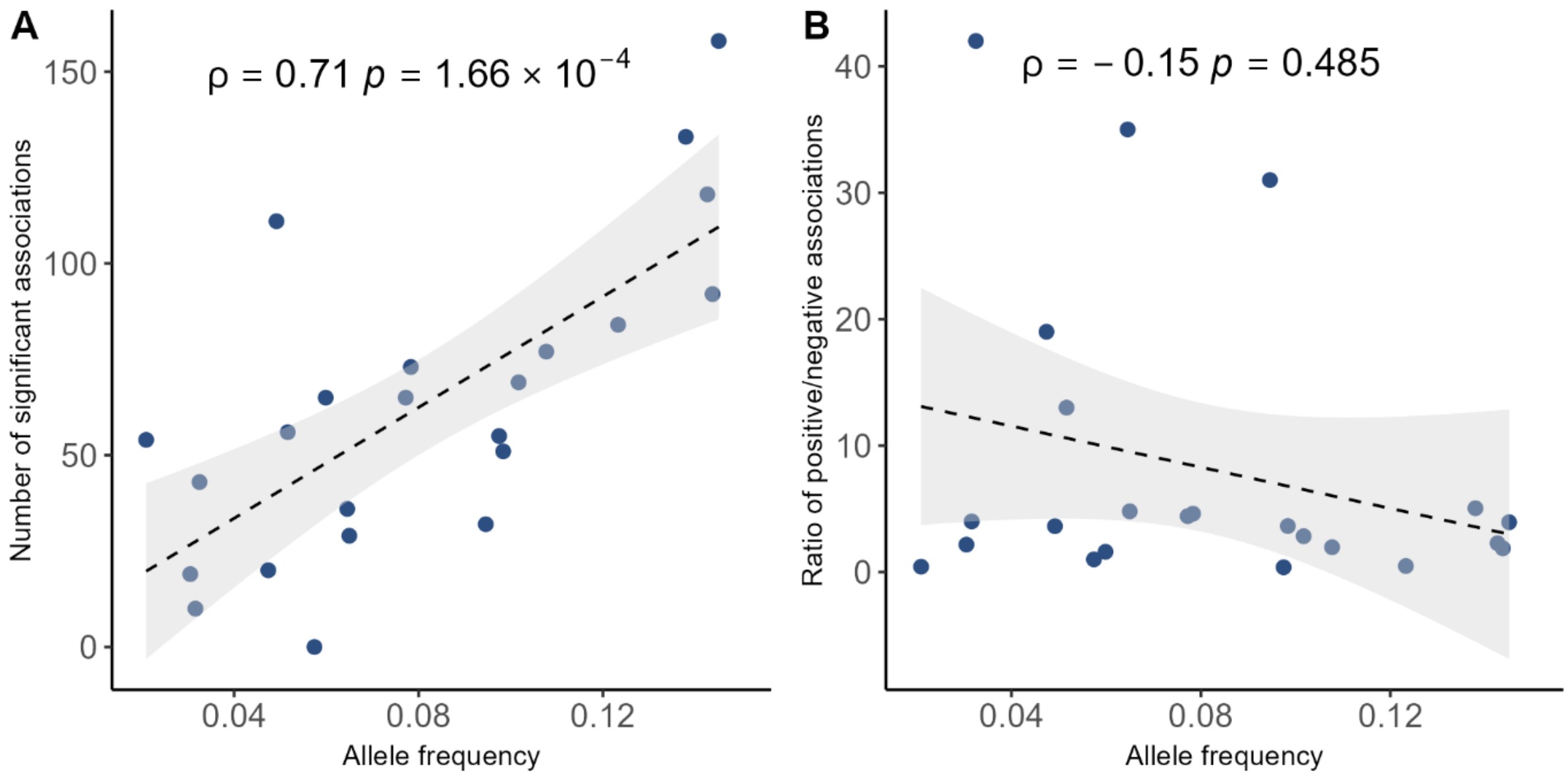

Supplementary Fig. 4. The ratio of positive and negative associations is independent of HLA-II allele frequency. The number of all significant associations (A) and the ratio of positive and negative associations (B) are shown as a function of allele frequency in the LLD and 1000IBD cohorts. Spearman's rho and two-sided p values of correlation tests are indicated.

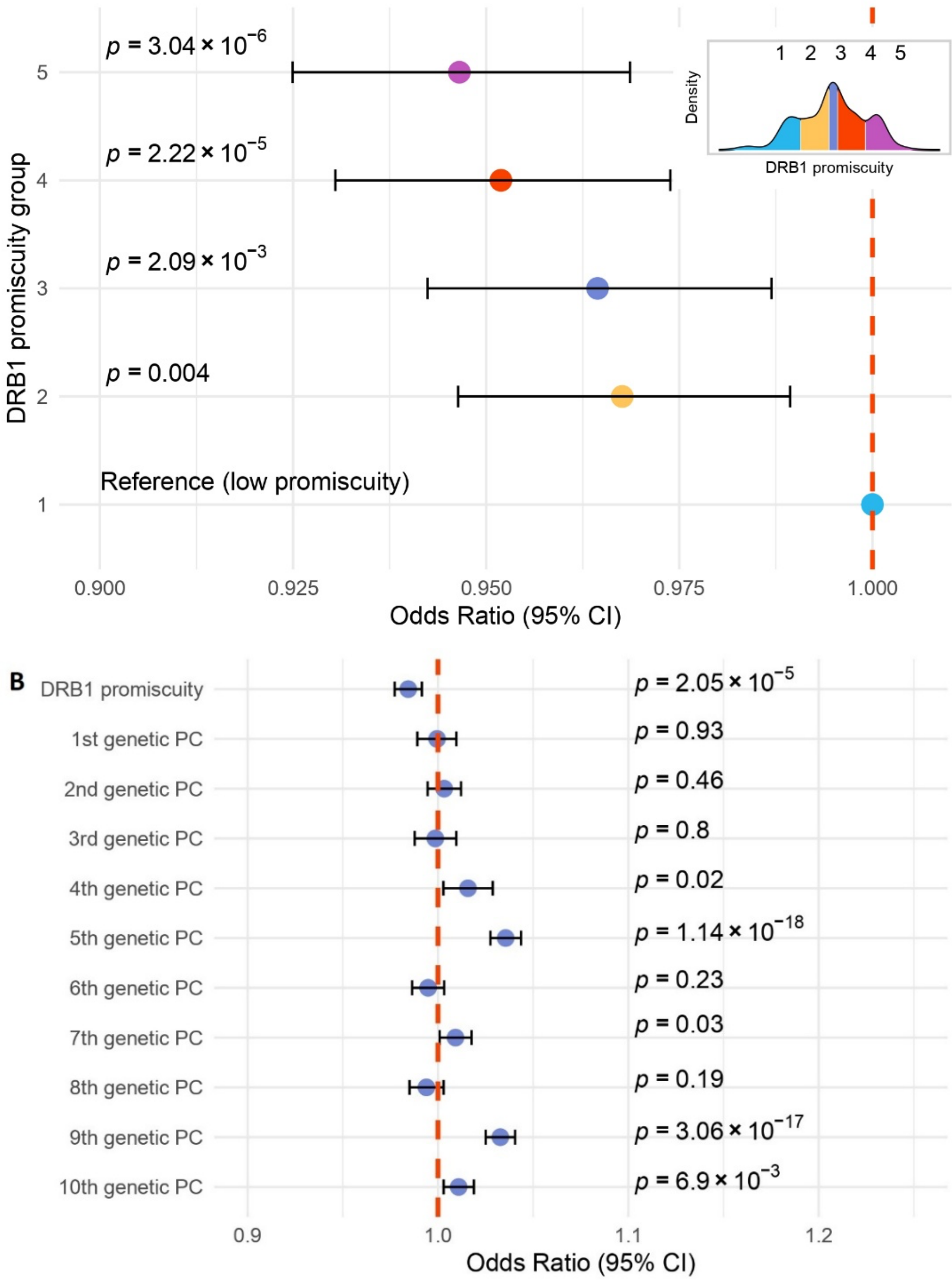

Supplementary Fig. 5. Consistent association between HLA-DRB1 promiscuity and infection risk when focusing on the British ancestry. The figure indicates the summary of logistic regression models restricted to individuals with British ancestry (n = 402,632). A) Higher HLA-DRB1 promiscuity is linked to a lower risk for severe infections. Patients were classified into five equal-sized groups with increasing promiscuity levels. The plot depicts the results of a logistic regression model involving hospitalization due to infection as a dependent and promiscuity groups as independent variables. Points represent the odds ratio for groups with increasing promiscuity compared to the lowest promiscuity group. Horizontal lines indicate the corresponding 95% confidence interval (CI). B) The impact of HLA-DRB1 promiscuity remains significant after adjusting for genetic principal components. The summary of a logistic regression model including the first ten genetic principal components and HLA-DRB1 promiscuity as independent variables. Points represent the odds ratio, while horizontal lines depict the 95% CI.

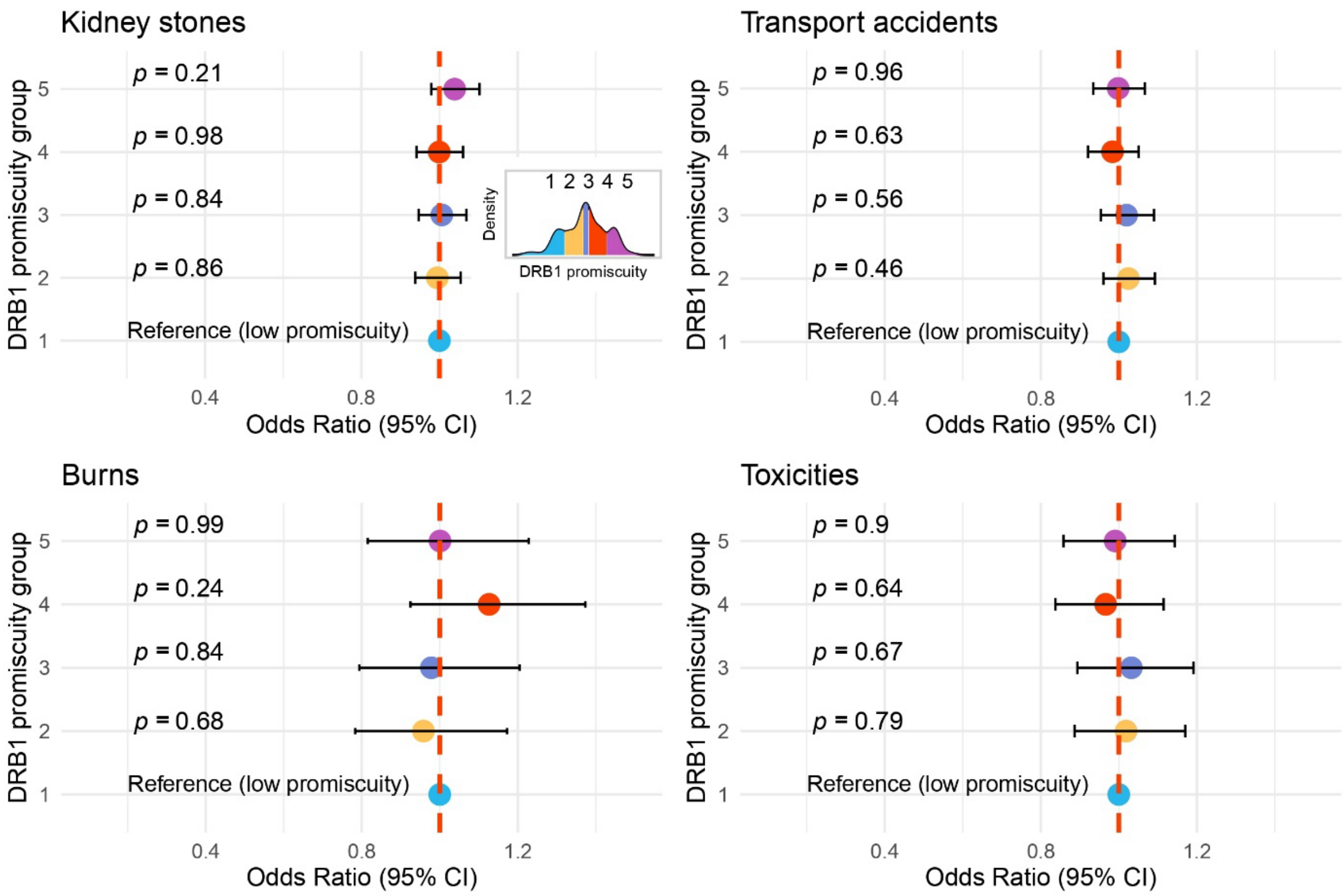

Supplementary Fig 6. Lack of association between HLA-DRB1 promiscuity and non-immune-related conditions. The figure indicates the potential association of HLA-DRB1 promiscuity with conditions unrelated to the immune system. Four logistic regression models were constructed including the occurrence of four different groups of diagnoses as dependent variables: A) kidney stones (ICD N20-N23), B) transport accidents (ICD V00-V99), C) burns (ICD T20-T32), and D) toxicities (ICD T51-T65). Participants were grouped into five equal-sized categories based on increasing levels of HLA-DRB1 promiscuity. Odds ratios are presented for each group relative to the lowest promiscuity group, with horizontal lines indicating 95% confidence intervals (CIs).

		Pathogens			Non-pathogens		
**HLA allele**	***Pr***	**$N_{positive}$**	**$N_{negative}$**	$\frac{N_{positive}+1}{N_{negative}+1}$	**$N_{positive}$**	**$N_{negative}$**	$\frac{N_{positive}+1}{N_{negative}+1}$
DQA1*01:01-DQB1*06:02	1.834	6	31	0.219	1	2	0.667
DQA1*03:01-DQB1*03:01	2.570	32	16	1.941	2	5	0.500
DPA1*02:01-DPB1*04:01	1.564	0	0	1.000	0	0	1.000
DQA1*01:02-DQB1*06:04	1.650	51	21	2.364	23	1	12.000
DQA1*01:01-DQB1*05:01	1.515	20	39	0.525	2	8	0.333
DQA1*01:02-DQB1*06:02	2.688	34	30	1.129	22	3	5.750
DQA1*03:01-DQB1*03:02	2.979	36	17	2.056	3	4	0.800
DQA1*05:01-DQB1*03:01	2.328	43	12	3.385	6	3	1.750
DQA1*02:01-DQB1*03:03	3.109	22	0	23.000	12	0	13.000
DQA1*05:01-DQB1*02:01	3.352	98	20	4.714	12	4	2.600
DQA1*01:03-DQB1*06:03	2.301	43	5	7.333	5	0	6.000
DPA1*02:01-DPB1*01:01	2.923	20	4	4.200	1	0	2.000
DQA1*02:01-DQB1*02:02	3.613	23	0	24.000	6	0	7.000
DRB1*01:01	2.721	10	27	0.393	1	6	0.286
DRB1*03:01	2.173	79	12	6.154	13	2	4.667
DRB1*04:01	2.483	25	7	3.250	2	2	1.000
DRB1*04:04	2.303	9	5	1.667	0	0	1.000
DRB1*07:01	2.709	21	0	22.000	4	0	5.000
DRB1*08:01	2.821	3	1	2.000	3	0	4.000
DRB1*11:01	2.758	8	0	9.000	9	0	10.000
DRB1*13:01	2.773	42	4	8.600	3	0	4.000
DRB1*13:02	2.744	31	1	16.000	15	0	16.000
DRB1*15:01	2.222	22	26	0.852	15	3	4.000

Supplementary Table 1. The promiscuity and the number of positive and negative associations for HLA-II alleles.

Taxon	Pth	Freq	Taxon	Pth	Freq
Adenoviridae	T	T	Human poliovirus	T	F
Adenoviridae?	T	T	Human poliovirus 3	T	F
Adenovirus C	T	T	Human respiratory syncytial virus	T	T
Akkermansia	F	F	Human rhinovirus	T	T
Akkermansia muciniphila	F	F	Human Rhinovirus Serotype 2	T	T
Akkermansia muciniphila ATCC BAA-835	F	F	Influenza A virus	T	T
Alistipes	F	F	Influenza B virus	T	T
Alternaria alternata (or arborescens?) fungus	F	F	Japanese encephalitis virus	T	F
Alternaria alternata (plant fungus)	F	F	Klebsiella phage	F	
Apibacter	F	F	Klebsiella phage 0507-KN2-1	F	
Aspergillus fischeri or Aspergillus fumigatus	T	T	Klebsiella phage JD18	F	
Aspergillus fumigatus	T	T	Klebsiella phage phiKO2	F	
Aspergillus rhizopodus (fungus)	T	T	Lachnospiraceae	F	
Bacillaceae	F		Lactobacillales	F	
Bacilli	F		Lactobacillus acidophilus NCFM	F	
Bacillus	T	T	Lactobacillus fermentum IFO 3956	F	
Bacillus cereus ATCC 14579	T	T	Lactobacillus gasseri ATCC 33323 = JCM 1131	F	
Bacillus phage B4	F		Lactobacillus johnsonii NCC 533	F	
Bacillus phage PBC1	F		Lactobacillus phage ATCC 8014-B2	F	
Bacillus subtilis	F		Lactobacillus phage A2	F	
Bacteria	F		Lactobacillus phage ATCC 8014-B2	F	
Bacteroidaceae	F		Lactobacillus phage JCL1032	F	
Bacteroidales	F		Lactobacillus phage KC5a	F	
Bacteroides	F		Lactobacillus phage LBR48	F	
Bacteroides dorei CL03T12C01	F		Lactobacillus phage Lc-Nu	F	
Bacteroides fragilis YCH46	F		Lactobacillus phage Ld17	F	
Bacteroides ovatus	F		Lactobacillus phage LF1	F	
Bacteroides phage B124-14	F		Lactobacillus phage LfeInf	F	
Bacteroides uniformis	F		Lactobacillus phage Lv-1	F	
Bacteroides uniformis ATCC 8492	F		Lactobacillus phage phi jlb1	F	
Bacteroides vulgatus ATCC 8482	F		Lactobacillus phage phiAQ113	F	
Bacteroides/Pho+A33:A101caeicola	F		Lactobacillus phage phig1e	F	
Bartonella henselae	T	T	Lactobacillus phage PL-1	F	
Bifidobacterium	F		Lactobacillus plantarum WCFS1	F	
Bifidobacterium adolescentis ATCC 15703	F		Lactobacillus prophage Lj771	F	
Bifidobacterium animalis subsp. lactis DSM 10140	F		Lactobacillus reuteri DSM 20016	F	
Bifidobacterium bifidum PRL2010	F		Lactobacillus rhamnosus GG	F	
Bifidobacterium breve UCC2003	F		Lactobacillus salivarius UCC118	F	
Bifidobacterium longum 35624	F		Lactoccocus phage WP-2	F	
Blautia obeum ATCC 29174	F		Lactococcus phage 936	F	
Blautia producta	F		Lactococcus phage bIL285	F	
Bordetella	T	T	Lactococcus phage phi145	F	
Bordetella pertussis	T	T	Legionella	T	T
Bordetella pertussis Tohama I	T	T	Legionella pneumophila	T	T
Borreliaceae	F		Legionellaceae	F	
Burkholderia pseudomallei	T	F	Listeria monocytogenes	T	T
Burkholderiaceae	F		Listeria phage	F	
Butyrivibrio	F		Listeria phage 2389	F	

Campylobacter coli	T	T	Listeria phage List-36	F	
Campylobacter jejuni	T	T	Listeria phage vB_LmoS_293	F	
Campylobacter phage CP30A	F		Mycobacteriaceae	F	
Campylobacter phage PC14	F		Mycobacterium tuberculosis	T	T
Campylobacter virus CP21	F		Mycobacterium tuberculosis complex	T	T
Campylobacter virus CPt10	F		Mycoplasma hyopneumoniae	F	
Candida albicans	T	T	Mycoplasma penetrans	T	F
Candidatus Gastranaerophilales bacterium	F		Mycoplasma pneumoniae	T	T
cellular organisms	F		Mycoplasma pneumoniae M129	T	T
Chikungunya virus	T	F	Neisseria	T	T
Chlamydia trachomatis	T	T	Neisseria meningitidis	T	T
Clostridia bacterium UC5.1-1D1	F		Neisseria meningitidis MC58	T	T
Clostridiales	F		Neisseriaceae	F	
Clostridiales bacterium 42_27	F		Norwalk virus	T	T
Clostridium	T	T	Odoribacter	F	
Clostridium botulinum	T	F	Oscillibacter sp.	F	
Clostridium perfringens	T	T	Oscillibacter sp. 57_20	F	
Clostridium perfringens ATCC 13124	T	T	Papillomaviridae	T	T
Clostridium phage phi24R	F		Parabacteroides	F	
Clostridium phage phiCP34O	F		Parabacteroides merdae	F	
Clostridium phage phiCT19406A	F		Paraprevotella	F	
Clostridium sp. AM49-4BH	F		Pasteurellaceae	F	
Clostridium sp. CAG:609	F		Pelagibacter phage HTVC010P	F	
Clostridium tetani	T	T	Plasmodium falciparum	T	T
Collinsella	F		Pneumoviridae	T	T
Coprococcus	F		Porphyromonas gingivalis	T	T
Coriobacteriaceae	F		Prevotella	F	
Coriobacteriia bacterium	F		Prevotella copri	F	
Coxsackievirus	T	T	Prevotella sp. 885	F	
Coxsackievirus A9	T	T	Prevotella sp. AM42-24	F	
Deep-sea thermophilic phage D6E	F		Prevotella sp. CAG:1031	F	
Dengue virus	T	F	Prevotella sp. CAG:279	F	
Dialister	F		Prevotella sp. CAG:592	F	
Dorea	F		Proteobacteria	F	
Dorea formicigenerans ATCC 27755	F		Proteus phage PM 75	F	
Dorea longicatena DSM 13814	F		Pseudomonadaceae	F	
Ebolavirus	T	F	Pseudomonas aeruginosa	T	T
Entamoeba histolytica	T	T	Pseudomonas phage 73	F	
Enterobacter phage Arya	F		Pseudomonas virus D3112	F	
Enterobacter phage E-2	F		Ralstonia solanacearum	F	
Enterobacter phage phiEap-1	F		Respiratory syncytial virus (RSV)	T	T
Enterobacterales	F		Rhinovirus B	T	T
Enterobacteria phage	F		Rhinovirus serotype 2	T	T
Enterobacteria phage 13a	F		Rhizobiales	F	
Enterobacteria phage 933W	F		Rikenellaceae	F	
Enterobacteria phage Phi1	F		Romboutsia timonensis	F	
Enterobacteria phage Sf101	F		root	F	
Enterobacteria phage Sf6	F		root (flagellin. many bacteria)	F	
Enterobacteria phage T7M	F		Roseburia	F	
Enterobacteriaceae	F		Roseburia faecis	F	
Enterococcus faecalis V583	T	T	Roseburia inulinivorans DSM 16841	F	
Enterococcus phage phiFL1C	F		Rubella virus	T	T
Enterovirus	T	T	Ruminococcaceae	F	
Enterovirus A	T	T	Ruminococcus	F	
Enterovirus A71	T	T	Ruminococcus bromii	F	

Enterovirus B	T	T	Ruminococcus gnavus AGR2154	F	
Enterovirus C	T	T	Ruminococcus torques ATCC 27756	F	
Enterovirus_Coxsackievirus	T	T	Salmonella	T	T
epicoccum?	F		Salmonella enterica	T	T
Erythroparvovirus	T	T	Salmonella phage 100268_sal2	F	
Escherichia	F		Salmonella phage BPS11Q3	F	
Escherichia coli	T	T	Salmonella phage f18SE	F	
Escherichia coli CFT073	T	T	Salmonella phage FelixO1	F	
Escherichia coli Nissle 1917	F		Salmonella phage SSE121	F	
Escherichia coli O127:H6 str. E2348/69	T	T	Salmonella phage STML-198	F	
Escherichia coli O157:H7 str. EDL933	T	T	Salmonella phage vB_SenMS16	F	
Escherichia coli O157:H7 str. Sakai	T	T	SARS coronavirus	T	F
Escherichia phage ECML-134	F		Shigella flexneri 2a str. 301	T	T
Escherichia phage vB_EcoM_JS09	F		Shigella phage POCJ13	F	
Escherichia virus P1	F		Shigella phage pSf-1	F	
Escherichia virus TLS	F		Shigella phage SHFML-11	F	
Eubacterium	F		Shigella phage SP18	F	
Eubacterium hallii DSM 3353	F		Shigella sonnei 53G	T	T
Eubacterium rectale ATCC 33656	F		Staphylococcaceae	F	
Eubacterium rectale ATCC 33656 & Roseburia inulinivorans DSM 16841	F		Staphylococcus	T	T
Eubacterium rectale ATCC 33656 & Roseburia inulinivorans DSM 16841 flagellin	F		Staphylococcus aureus	T	T
Eubacterium rectale ATCC 33656_Flagellin	F		Staphylococcus aureus Clumping factor B	T	T
Faecalibacterium	F		Staphylococcus aureus subsp. aureus MRSA252	T	T
Faecalibacterium prausnitzii	F		Staphylococcus aureus subsp. aureus MW2	T	T
Faecalibacterium prausnitzii A2-165	F		Staphylococcus aureus subsp. aureus str. Newman	T	T
Firmicutes	F		Staphylococcus aureus/Enterococcus_lipase	T	T
Flavonifractor sp. An306	F		Staphylococcus phage 11	F	
Foot-and-mouth disease virus - type O	T	T	Staphylococcus phage MSA6	F	
Gammaproteobacteria	F		Staphylococcus phage P108	F	
Geobacillus phage GBK2	F		Streptococcus	T	T
Haemophilus influenzae	T	T	Streptococcus agalactiae	T	T
Haemophilus influenzae C54	T	T	Streptococcus dysgalactiae	T	T
Haemophilus influenzae nontypable strain 3179B	T	T	Streptococcus lutetiensis 033	T	T
Haemophilus influenzae Rd KW20	T	T	Streptococcus phage	F	
Haemophilus influenzae str. 12	T	T	Streptococcus phage TP-778L	F	
Haemophilus parainfluenzae T3T1	F		Streptococcus pneumoniae	T	T
Helicobacter pylori	T	T	Streptococcus pneumoniae R6	T	T
Helicobacter pylori 26695	T	T	Streptococcus pneumoniae TIGR4	T	T
Hepatitis C virus	T	T	Streptococcus pyogenes	T	T
Hepatitis C virus subtype 1a	T	T	Streptococcus pyogenes M1 GAS	T	T
Hepatitis E virus	T	T	Streptococcus pyogenes MGAS315	T	T
Human adenovirus 12	T	T	Streptococcus pyogenes MGAS8232	T	T
Human adenovirus 5; Human adenovirus 2	T	T	Streptococcus sp. G148	T	T
Human adenovirus C	T	T	Streptococcus virus ALQ132	F	
Human alphaherpesvirus	T	T	Subdoligranulum sp. 60_17	F	

Human alphaherpesvirus 1	T	T	Terrabacteria group	F	
Human alphaherpesvirus 2	T	T	Toxoplasma gondii	T	T
Human alphaherpesvirus 3	T	T	Trichomonas vaginalis	T	T
Human betaherpesvirus 5 (CMV)	T	T	unclassified Akkermansia	F	
Human betaherpesvirus 6?	T	T	unclassified Alistipes	F	
Human betaherpesvirus 7	T	T	unclassified Blautia	F	
Human gammaherpesvirus 4 (EBV)	T	T	uncultured Eubacterium sp.	F	
Human hepatitis A virus	T	T	Vibrio cholerae AM-19226	T	T
Human herpesvirus 1	T	T	Vibrio vulnificus	T	T
Human herpesvirus 1 (strain 17)	T	T	Vibrionaceae	F	
Human herpesvirus 2	T	T	West Nile virus	T	F
Human herpesvirus 3	T	T	Xanthomonas	F	
Human immunodeficiency virus 1	T	T	Yersinia	T	T
Human mastadenovirus	T	T	Yersinia pestis CO92	T	F
Human papillomavirus type 16	T	T			

Supplementary Table 2. The list and classification of microbes included in the analysis. Taxa are listed as in the meta-data of the original article (25). The column “Pth.” indicates the classification of taxa: T (true): pathogen, F (false): non-pathogen or classification is not applicable. The column “Freq” indicates whether the pathogenic taxa are endemic in the Netherlands. T (true): common, F (false) not common. Classification was carried out based on data in the GIDEON database (13, 26).

**Variable**	**Pathogens**<br>Coefficient<br>(p-value)	**Non-pathogens**<br>Coefficient<br>(p-value)
Allele promiscuity	7.48<br>(**0.012**)	1.465<br>(0.434)
DQ (ref. DP)	1.61<br>(0.763)	2.648<br>(0.468)
DRB1 (ref. DP)	1.94<br>(0.719)	3.016<br>(0.414)

Supplementary Table 3. The relationship between HLA-antibody associations and promiscuity is independent of the HLA-II loci. We constructed two multivariate linear regression models separately for pathogens and non-pathogens as follows: ratio ~ Pr + group, where the dependent variable is the ratio of positive and negative associations for each HLA-II variant, Pr is allele promiscuity, group is the type of HLA-II molecules (i.e., DP, DQ and DRB1; DP was the reference class in the model). Significant p-values of two-sided t-tests is highlighted with bold character. The presence of heteroscedasticity is unlikely in the models according to Breusch-Pagan tests (p = 0.088 and 0.837 for models involving pathogens and non-pathogens, respectively).

ICD	Description	Count	p value	FDR	(-logFDR)	HR	log2HR
**A04**	Other bacterial intestinal infections	8013	9.98E-01	9.98E-01	0.001	1.000	0.000
**A08**	Viral and other specified intestinal infections	5198	1.33E-01	3.56E-01	0.448	0.898	-0.155
**A09**	Infectious gastroenteritis and colitis, unspecified	24417	3.74E-01	5.57E-01	0.254	0.971	-0.042
**A40**	Streptococcal sepsis	850	4.02E-01	5.57E-01	0.254	0.861	-0.216
**A41**	Other sepsis	16680	2.04E-02	9.45E-02	1.024	0.911	-0.134
**A51**	Early syphilis, symptomatic	20	2.01E-01	4.46E-01	0.350	0.234	-2.095
**A52**	Late syphilis	15	2.59E-01	5.08E-01	0.294	4.634	2.212
**A53**	Other and unspecified syphilis	24	3.58E-01	5.57E-01	0.254	2.665	1.414
**A54**	Gonococcal infection	257	3.83E-01	5.57E-01	0.254	1.326	0.407
**A56**	Other sexually transmitted chlamydia diseases	72	9.89E-02	2.80E-01	0.552	0.371	-1.431
**A59**	Trichomoniasis	78	1.41E-01	3.59E-01	0.445	2.388	1.256
**A60**	Anogenital herpesviral infection	413	4.55E-01	5.76E-01	0.239	1.210	0.275
**A63**	Other predominantly sexually transmitted diseases, not elsewhere classified	1676	4.53E-17	2.31E-15	14.637	0.351	-1.510
**B00**	Herpesviral infections	4706	1.56E-01	3.78E-01	0.423	0.898	-0.155
**B01**	Varicella [chickenpox]	23698	6.46E-01	7.11E-01	0.148	0.985	-0.022
**B02**	Zoster [shingles]	17615	5.93E-07	1.01E-05	4.996	0.823	-0.281
**B05**	Measles	16869	1.84E-01	4.27E-01	0.370	0.949	-0.076
**B06**	Rubella [German measles]	6051	1.99E-02	9.45E-02	1.024	1.168	0.224
**B07**	Viral warts	16293	6.83E-11	1.74E-09	8.759	0.768	-0.381
**B08**	Other viral infections characterized by skin and mucous membrane lesions	955	2.28E-01	4.64E-01	0.333	0.817	-0.292
**B09**	Unspecified viral infection characterized by skin and mucous membrane lesions	27	4.01E-01	5.57E-01	0.254	2.328	1.219
**B25**	Cytomegaloviral disease	272	3.32E-01	5.57E-01	0.254	0.738	-0.438
**B27**	Infectious mononucleosis	3616	3.56E-01	5.57E-01	0.254	1.083	0.115
**B35**	Dermatophytosis	30060	7.89E-02	2.67E-01	0.574	0.949	-0.076
**B36**	Other superficial mycoses	5320	5.80E-01	6.72E-01	0.173	0.961	-0.057
**B37**	Candidiasis	12217	2.23E-04	1.90E-03	2.722	0.841	-0.250
**J00**	Acute nasopharyngitis [common cold]	5090	3.99E-01	5.57E-01	0.254	0.941	-0.088
**J01**	Acute sinusitis	22472	9.37E-02	2.80E-01	0.552	0.944	-0.083
**J02**	Acute pharyngitis	22213	4.26E-01	5.57E-01	0.254	1.028	0.040
**J03**	Acute tonsillitis	18929	8.37E-02	2.67E-01	0.574	1.067	0.094
**J04**	Acute laryngitis and tracheitis	4237	5.68E-01	6.72E-01	0.173	0.956	-0.065
**J05**	Acute obstructive laryngitis [croup] and epiglottitis	172	4.63E-01	5.76E-01	0.239	1.338	0.420
**J06**	Acute upper respiratory infections of multiple and unspecified sites	47355	4.25E-01	5.57E-01	0.254	1.019	0.027
**J09**	Influenza due to identified zoonotic or pandemic influenza virus	25	9.34E-01	9.72E-01	0.012	0.917	-0.125
**J10**	Influenza due to other identified influenza virus	1602	2.16E-01	4.59E-01	0.338	1.175	0.233
**J11**	Influenza, virus not identified	10026	9.90E-01	9.98E-01	0.001	1.001	0.001
**J12**	Viral pneumonia, not elsewhere classified	3443	6.29E-01	7.11E-01	0.148	1.044	0.062

**J13**	Pneumonia due to Streptococcus pneumoniae	1012	7.69E-01	8.17E-01	0.088	0.953	-0.069
**J14**	Pneumonia due to Haemophilus influenzae	258	5.80E-01	6.72E-01	0.173	0.836	-0.258
**J15**	Bacterial pneumonia, not elsewhere classified	1537	4.03E-01	5.57E-01	0.254	0.895	-0.160
**J16**	Pneumonia due to other infectious organisms, not elsewhere classified	86	4.10E-01	5.57E-01	0.254	1.590	0.669
**J17**	Pneumonia in diseases classified elsewhere	378	6.55E-01	7.11E-01	0.148	1.127	0.172
**J18**	Pneumonia, unspecified organism	36694	7.80E-04	5.68E-03	2.246	0.913	-0.131
**J20**	Acute bronchitis	5039	1.84E-02	9.45E-02	1.024	0.842	-0.248
**J21**	Acute bronchiolitis	171	3.85E-01	5.57E-01	0.254	1.423	0.509
**J22**	Unspecified acute lower respiratory infection	59797	2.72E-03	1.74E-02	1.761	0.938	-0.092
**J36**	Peritonsillar abscess	895	7.18E-02	2.67E-01	0.574	0.733	-0.448
**J86**	Pyothorax	794	8.02E-02	2.67E-01	0.574	0.725	-0.464
**L03**	Cellulitis	31087	8.97E-06	9.15E-05	4.039	0.878	-0.188
**L08**	Other local infections of skin and subcutaneous tissue	14712	1.93E-06	2.46E-05	4.609	0.816	-0.293
**N39**	Other disorders of urinary system	54494	6.89E-02	2.67E-01	0.574	0.960	-0.059

Supplementary Table 4. List of infectious diseases involved in the study. ICD stands for ICD-10 code. For each diagnosis, the table includes the number of cases (Count), the hazard ratio value associated with promiscuity (HR), log2-transformed hazard ratio (log2HR), nominal p-values of two-sided log-rank tests, and multiple-testing–adjusted significance (FDR and −log10 FDR).

Infectious disease occurrence with hospitalization			
Category	Subcategory	ICD	Frequency
Respiratory infections<br>n = 85,340	Other acute lower respiratory infections<br>n = 19,777	J20	443
		J21	126
		J22	19,208
	Acute upper respiratory infections<br>n = 6,260	J00	768
		J02	1,949
		J03	952
		J06	1,741
		Other (J01, J04, J05)	850
	Influenza and pneumonia<br>n = 43,473	J09	20
		J10	1,654
		J11	404
		J12	3,745
		J18	34,726
		Other (J13 - J17)	2,924
	Others<br>n = 15,830	J36	671
		J86	818
		U07	14,341
Common mycoses<br>n = 9,147	NA	B35	758
		B36	193
		B37	8,196
Gastrointestinal infections<br>n = 30,570	NA	A04	5,508
		A08	2,211
		A09	22,851
Skin infections and sexually transmitted diseases<br>n = 25,936	Bacterial skin infections<br>n = 21,555	L03	17,837
		L08	3,718
	Sexually transmitted diseases<br>n = 356	A60	44
		A63	263
		Other (A51, A52, A53, A54, 56, A58, A59)	49
	Viral infections characterized by skin and mucous membrane lesions<br>n = 4,025	B00	750
		B01	132
		B02	1,548
		B07	1,471
		Other (B05, B06, B08, B09)	124
Infections of the urogenital tract<br>n = 45,149	NA	N39	45,149
Others<br>n = 20,480	NA	B25	294
		B27	109
	Septicaemia<br>n = 20,077	A40	938
		A41	19,139

Supplementary Table 5. Classification and prevalence of infectious diseases in the UK Biobank cohort. ICD stands for ICD-10 code.

Variable	Hazard ratio	p-value
DRB1 promiscuity	0.908	1.29×10^{-7}
DRB1 HED	1.001	0.45
DRB1 homozygosity	1.055	4.92×10^{-4}

Supplementary Table 6. The impact of HLA-DRB1 promiscuity after controlling for HLA-associated factors. This table summarizes the associations of HLA-DRB1 promiscuity, homozygosity, and HED with risk for hospitalization. HLA-DRB1 promiscuity shows a significant protective effect, while homozygosity is linked to increased risk. p values of two-sided Wald z-tests are indicated.

Variable	Hazard ratio	p-value
DRB1 promiscuity	0.933	2.01×10^{-4}
Age (year)	1.036	$< 2.22 \times 10^{-16}$
Sex (female vs. male)	1.002	0.806
Immunodeficiency (binary)	3.618	$< 2.22 \times 10^{-16}$

Supplementary Table 7. The effect of HLA-II promiscuity on the occurrence of severe infections remains significant after controlling for age, sex and immunodeficiency. The summary of the logistic regression model “infection ~ Pr + Age + Sex + Immunodeficiency”, where the dependent variable indicates infectious episode with hospitalization. p values of two-sided Wald z-tests are indicated.

Variable	Hazard ratio	p-value
DRB1 promiscuity	0.898	5.82E-06
A_0201	1.006	0.47
A_0205	0.988	0.70
B_0801	0.989	0.23
B_4402	1.014	0.16
B_5701	0.993	0.62
DQB1_0301	0.974	0.01
DQB1_0602	0.974	0.43
DRB1_0401	1.028	7.09E-03
DRB1_1101	1.020	0.25
DRB1_1501	1.035	0.31

Supplementary Table 8. The effect of HLA promiscuity remains significant after controlling for alleles associated with common infectious diseases. The table summarizes a logistic regression model containing the occurrence of severe infections as a dependent variable. The coefficients and p-values of two-sided Wald z-tests for independent variables are indicated. Carrying different risk alleles were included as binary variables. HLA alleles associated with risk for common infections were identified in a previous GWAS (31). The model included only individuals belonging to the British ancestry because the GWAS was carried out on Caucasian ethnic groups.

Variable	Hazard ratio	p-value
**A63: Other predominantly sexually transmitted diseases**		
DRB1 promiscuity	0.330	3.93E-17
C_1601	0.831	0.062
DRB1_0407	1.504	5.85E-03
DRB1_1501	0.887	0.036
**B07: Viral warts**		
DRB1 promiscuity	0.835	4.03E-05
C_1601	0.981	0.51
DRB1_0407	1.041	0.48
DRB1_1501	1.098	4.67E-07
**L08: Other local infections of skin and subcutaneous tissue**		
DRB1 promiscuity	0.817	2.14E-05
DPB1_0401	0.986	0.44
DQB1_0201	1.004	0.86
**B02: Herpes zoster**		
DRB1 promiscuity	0.806	2.30E-06
B_4402	0.890	8.01E-04
C_0501	0.914	8.92E-03
DQA1_0101	0.910	0.018
DQB1_0202	0.996	0.86
DQB1_0501	0.979	0.66
DQB1_0604	1.035	0.32
DRB1_0101	1.108	7.25E-03
**B37: Candidiasis**		
DRB1 promiscuity	0.821	3.95E-05
DRB1_1104	0.975	0.66
DRB1_0901	1.140	0.018
**L03: Cellulitis**		
DRB1 promiscuity	0.867	5.66E-06
A_3101	0.963	0.15
B_1402	1.041	0.35
C_0602	0.994	0.70
C_0802	0.991	0.80
DPB1_0101	0.994	0.72
DQB1_0402	1.021	0.47
DQB1_0302	1.023	0.12
DQB1_0303	1.034	0.11
**J18: Pneumonia, unspecified organism**		
DRB1 promiscuity	0.906	7.19E-04
A_3001	1.029	0.43
B_3501	1.031	0.22
C_0401	1.000	0.99
C_1601	0.967	0.10
DQA1_0201	0.969	0.73
DRB1_0701	1.046	0.62
**B20-B24: Human immunodeficiency virus**		
DRB1 promiscuity	2.383	1.17E-03
B_2705	0.806	0.27

B_3501	1.042	0.79
B_5701	0.838	0.34
DQA1_0102	1.335	0.045
DRB1_1501	0.754	0.10

Supplementary Table 9. The effect of DRB1 promiscuity on the incidence of infections remains significant after controlling for HLA alleles potentially affecting disease susceptibility. The table summarizes the results of multiple Cox proportional hazards models involving HLA promiscuity and HLA alleles potentially linked to diseases as independent variables. Alleles were selected using the PheWAS database (see Methods for details). Hazard ratio (HR) and p-value of a two-sided Wald test are shown for each independent variable. Models were built for HIV and all diseases associated with promiscuity in Figure 4D.
