# Supplementary material for: HLA-II peptide-binding diversity shapes humoral immune responses and susceptibility to infections
Source: Nat Commun. 2026 Aug 5;17:9422. doi: 10.1038/s41467-026-76184-1 (PMC13538663; doi:10.1038/s41467-026-76184-1)
Supplement: Supplementary file 2 [file 41467_2026_76184_MOESM2_ESM.pdf]

nature portfolio

Corresponding author(s): Mate Manczinger

Last updated by author(s): Jun 10, 2026

Reporting Summary

Nature Portfolio wishes to improve the reproducibility of the work that we publish. This form provides structure for consistency and transparency in reporting. For further information on Nature Portfolio policies, see our Editorial Policies and the Editorial Policy Checklist.

Statistics

For all statistical analyses, confirm that the following items are present in the figure legend, table legend, main text, or Methods section.

n/a	Confirmed	
☐	☒	The exact sample size (n) for each experimental group/condition, given as a discrete number and unit of measurement
☒	☐	A statement on whether measurements were taken from distinct samples or whether the same sample was measured repeatedly
☐	☒	The statistical test(s) used AND whether they are one- or two-sided<br>*Only common tests should be described solely by name; describe more complex techniques in the Methods section.*
☐	☒	A description of all covariates tested
☐	☒	A description of any assumptions or corrections, such as tests of normality and adjustment for multiple comparisons
☐	☒	A full description of the statistical parameters including central tendency (e.g. means) or other basic estimates (e.g. regression coefficient) AND variation (e.g. standard deviation) or associated estimates of uncertainty (e.g. confidence intervals)
☐	☒	For null hypothesis testing, the test statistic (e.g. F, t, r) with confidence intervals, effect sizes, degrees of freedom and P value noted<br>*Give P values as exact values whenever suitable.*
☒	☐	For Bayesian analysis, information on the choice of priors and Markov chain Monte Carlo settings
☒	☐	For hierarchical and complex designs, identification of the appropriate level for tests and full reporting of outcomes
☒	☐	Estimates of effect sizes (e.g. Cohen's d, Pearson's r), indicating how they were calculated

Our web collection on statistics for biologists contains articles on many of the points above.

Software and code

Policy information about availability of computer code

Data collection	No code was used to collect data.
Data analysis	The code used for calculating HLA promiscuity is publicly available on GitHub (https://github.com/matemanc/hla_promiscuity). Other code for generating results is also available on GitHub (https://github.com/matemanc/promisc_ab_publ). Scripts used for the analysis of the UK Biobank cohort are hosted within the UK Biobank Research Analysis Platform and are accessible to approved researchers following a successful application to the UK Biobank (https://www.ukbiobank.ac.uk/enable-your-research/apply-for-access).<br>R 4.3.2., protr 1.7-5, universalmotif 1.8.0

For manuscripts utilizing custom algorithms or software that are central to the research but not yet described in published literature, software must be made available to editors and reviewers. We strongly encourage code deposition in a community repository (e.g. GitHub). See the Nature Portfolio guidelines for submitting code & software for further information.

Data

Policy information about availability of data

All manuscripts must include a data availability statement. This statement should provide the following information, where applicable:

- Accession codes, unique identifiers, or web links for publicly available datasets
- A description of any restrictions on data availability
- For clinical datasets or third party data, please ensure that the statement adheres to our policy

The datasets analyzed in this study are available from the UK Biobank and the LifeLines DEEP cohort but are subject to access restrictions. Data from the UK Biobank

are available upon application and approval via their Access Management System (https://www.ukbiobank.ac.uk/enable-your-research/apply-for-access), which requires a detailed research proposal and agreement to data use terms. Similarly, access to the LifeLines DEEP data can be requested through a formal application process (https://www.lifelines.nl/researcher), including submission of a research plan and compliance with ethical and data privacy regulations. Data are available only for approved academic and health-related research purposes. Data from the Fluzone vaccine trial are available in the ImmPort database (study ID: SDY314) upon registration. Source Data are provided with this paper.

Research involving human participants, their data, or biological material

Policy information about studies with human participants or human data. See also policy information about sex, gender (identity/presentation), and sexual orientation and race, ethnicity and racism.

Reporting on sex and gender: Gender was not assessed in this study. Analyses were restricted to samples with concordant self-reported and genetically inferred sex, and sex was included as a covariate.

Reporting on race, ethnicity, or other socially relevant groupings: We used genetic principal components as covariates in models. To minimize potential confounding effects from ethnic groups, we repeated certain analysis including only individuals of British ancestry.

Population characteristics: We used existing cohorts. UK Biobank is a large prospective population cohort comprising about 502,000 volunteers recruited in 2006–2010 from England, Scotland, and Wales. Participants were 40–69 years old at recruitment, making the cohort mainly middle-aged to older adults at baseline. It includes extensive phenotypic, lifestyle, clinical, and genetic data.

LifeLines DEEP is a smaller, intensively characterized subcohort of the Dutch LifeLines study. It included 1,539 adults aged 18 years and older, recruited in Groningen in the northern Netherlands, with a slight female majority (58.7%) and a mean age of 44 years. Unlike the broader UK Biobank, LifeLines DEEP was designed for deep molecular phenotyping, with collection of blood, exhaled air, fecal samples, and multi-omics data such as genomic, transcriptomic, methylation, metabolomic, and microbiome profiles.

Recruitment: No participants were recruited for this study. Data was used from UK Biobank an LifeLined Deep.

Ethics oversight: UK Biobank and LifeLines DEEP were both conducted with ethics committee approval and written informed consent from participants. UK Biobank is governed under a dedicated Ethics and Governance Framework with independent ethical oversight, while LifeLines DEEP was approved by the ethics committee of the University Medical Center Groningen as a subcohort of the broader LifeLines study, which follows the principles of the Declaration of Helsinki (reference numbers M12.113965 and 2008/338).

This research was done under UKB application No. 272827

Note that full information on the approval of the study protocol must also be provided in the manuscript.

Field-specific reporting

Please select the one below that is the best fit for your research. If you are not sure, read the appropriate sections before making your selection.

[x] Life sciences [ ] Behavioural & social sciences [ ] Ecological, evolutionary & environmental sciences

For a reference copy of the document with all sections, see nature.com/documents/nr-reporting-summary-flat.pdf

Life sciences study design

All studies must disclose on these points even when the disclosure is negative.

Sample size: We used the maximum available number of participants with HLA genotyping data in both the UK Biobank and LifeLines DEEP cohort.

Data exclusions: Only individuals with inappropriate HLA genotyping data were excluded.

Replication: Reproducibility was supported using publicly described datasets and standardized computational workflows. As these cohorts represent some of the largest available sample sizes, no independent dataset of comparable scale was available for replication.

Randomization: Randomization is not relevant for the study. (The experiments were not randomized) Regression models included relevant covariates such as HLA group, age, sex, cohort, sequencing plate, genetic principal components, immunodeficiency status and infection-associated HLA alleles where applicable.

Blinding: Blinding is not relevant for the study. (The Investigators were not blinded)

Reporting for specific materials, systems and methods

We require information from authors about some types of materials, experimental systems and methods used in many studies. Here, indicate whether each material, system or method listed is relevant to your study. If you are not sure if a list item applies to your research, read the appropriate section before selecting a response.

Materials & experimental systems

n/a	Involved in the study
☐	☐ Antibodies
☐	☐ Eukaryotic cell lines
☐	☐ Palaeontology and archaeology
☐	☐ Animals and other organisms
☐	☒ Clinical data
☐	☐ Dual use research of concern
☐	☐ Plants

Methods

n/a	Involved in the study
☒	☐ ChIP-seq
☒	☐ Flow cytometry
☒	☐ MRI-based neuroimaging

Antibodies

Antibodies used

Describe all antibodies used in the study; as applicable, provide supplier name, catalog number, clone name, and lot number.

Validation

Describe the validation of each primary antibody for the species and application, noting any validation statements on the manufacturer's website, relevant citations, antibody profiles in online databases, or data provided in the manuscript.

Eukaryotic cell lines

Policy information about cell lines and Sex and Gender in Research

Cell line source(s)

State the source of each cell line used and the sex of all primary cell lines and cells derived from human participants or vertebrate models.

Authentication

Describe the authentication procedures for each cell line used OR declare that none of the cell lines used were authenticated.

Mycoplasma contamination

Confirm that all cell lines tested negative for mycoplasma contamination OR describe the results of the testing for mycoplasma contamination OR declare that the cell lines were not tested for mycoplasma contamination.

Commonly misidentified lines
(See ICLAC register)

Name any commonly misidentified cell lines used in the study and provide a rationale for their use.

Palaeontology and Archaeology

Specimen provenance

Provide provenance information for specimens and describe permits that were obtained for the work (including the name of the issuing authority, the date of issue, and any identifying information). Permits should encompass collection and, where applicable, export.

Specimen deposition

Indicate where the specimens have been deposited to permit free access by other researchers.

Dating methods

If new dates are provided, describe how they were obtained (e.g. collection, storage, sample pretreatment and measurement), where they were obtained (i.e. lab name), the calibration program and the protocol for quality assurance OR state that no new dates are provided.

☐ Tick this box to confirm that the raw and calibrated dates are available in the paper or in Supplementary Information.

Ethics oversight

Identify the organization(s) that approved or provided guidance on the study protocol, OR state that no ethical approval or guidance was required and explain why not.

Note that full information on the approval of the study protocol must also be provided in the manuscript.

Animals and other research organisms

Policy information about studies involving animals; ARRIVE guidelines recommended for reporting animal research, and Sex and Gender in Research

Laboratory animals

For laboratory animals, report species, strain and age OR state that the study did not involve laboratory animals.

Wild animals

Provide details on animals observed in or captured in the field; report species and age where possible. Describe how animals were caught and transported and what happened to captive animals after the study (if killed, explain why and describe method; if released, say where and when) OR state that the study did not involve wild animals.

Reporting on sex

Indicate if findings apply to only one sex; describe whether sex was considered in study design, methods used for assigning sex. Provide data disaggregated for sex where this information has been collected in the source data as appropriate; provide overall numbers in this Reporting Summary. Please state if this information has not been collected. Report sex-based analyses where performed, justify reasons for lack of sex-based analysis.

Field-collected samples	*For laboratory work with field-collected samples, describe all relevant parameters such as housing, maintenance, temperature, photoperiod and end-of-experiment protocol OR state that the study did not involve samples collected from the field.*
Ethics oversight	*Identify the organization(s) that approved or provided guidance on the study protocol, OR state that no ethical approval or guidance was required and explain why not.*

Note that full information on the approval of the study protocol must also be provided in the manuscript.

Clinical data

Policy information about clinical studies
All manuscripts should comply with the ICMJE guidelines for publication of clinical research and a completed CONSORT checklist must be included with all submissions.

Clinical trial registration	*Provide the trial registration number from ClinicalTrials.gov or an equivalent agency.*
Study protocol	*Note where the full trial protocol can be accessed OR if not available, explain why.*
Data collection	*Describe the settings and locales of data collection, noting the time periods of recruitment and data collection.*
Outcomes	*Describe how you pre-defined primary and secondary outcome measures and how you assessed these measures.*

Dual use research of concern

Policy information about dual use research of concern

Hazards

Could the accidental, deliberate or reckless misuse of agents or technologies generated in the work, or the application of information presented in the manuscript, pose a threat to:

No	Yes	
☐	☐	Public health
☐	☐	National security
☐	☐	Crops and/or livestock
☐	☐	Ecosystems
☐	☐	Any other significant area

Experiments of concern

Does the work involve any of these experiments of concern:

No	Yes	
☐	☐	Demonstrate how to render a vaccine ineffective
☐	☐	Confer resistance to therapeutically useful antibiotics or antiviral agents
☐	☐	Enhance the virulence of a pathogen or render a nonpathogen virulent
☐	☐	Increase transmissibility of a pathogen
☐	☐	Alter the host range of a pathogen
☐	☐	Enable evasion of diagnostic/detection modalities
☐	☐	Enable the weaponization of a biological agent or toxin
☐	☐	Any other potentially harmful combination of experiments and agents

Plants

Seed stocks	*Report on the source of all seed stocks or other plant material used. If applicable, state the seed stock centre and catalogue number. If plant specimens were collected from the field, describe the collection location, date and sampling procedures.*
Novel plant genotypes	*Describe the methods by which all novel plant genotypes were produced. This includes those generated by transgenic approaches, gene editing, chemical/radiation-based mutagenesis and hybridization. For transgenic lines, describe the transformation method, the number of independent lines analyzed and the generation upon which experiments were performed. For gene-edited lines, describe the editor used, the endogenous sequence targeted for editing, the targeting guide RNA sequence (if applicable) and how the editor was applied.*
Authentication	*Describe any authentication procedures for each seed stock used or novel genotype generated. Describe any experiments used to assess the effect of a mutation and, where applicable, how potential secondary effects (e.g. second site T-DNA insertions, mosiacism, off-target gene editing) were examined.*
