# Supplementary material for: HLA-II peptide-binding diversity shapes humoral immune responses and susceptibility to infections
Source: Nat Commun. 2026 Aug 5;17:9422. doi: 10.1038/s41467-026-76184-1 (PMC13538663; doi:10.1038/s41467-026-76184-1)
Supplement: Supplementary file 3 [file 41467_2026_76184_MOESM3_ESM.pdf]

nature portfolio

Peer Review File

HLA-II peptide-binding diversity shapes humoral immune responses and susceptibility to infections

Corresponding Author: Dr Mate Manczinger

This file contains all reviewer reports in order by version, followed by all author rebuttals in order by version.

Version 0:

Reviewer comments:

Reviewer #1

(Remarks to the Author)
Magyari et al evaluate class II HLA molecules according to their peptide presentation characteristics, assigning a promiscuity index to individual class II HLA molecules according to the amino acid composition of their peptide ligands. The study then segues to test whether HLA II with higher Pr are likely to bind more pathogen derived peptides. Lastly, the study links HLA II promiscuity to the presence of antibodies to pathogens and the control of infectious disease. The study concludes/suggests that promiscuous HLA II molecules protect from a variety of infections while low promiscuity HLA II defend against emerging pathogens. A primary concern is that the three segments of the study appear to have been completed in a manner that selectively support the contribution of promiscuity towards controlling infection. It would have been helpful to have the study focused on P1 P4 P6 and P9 anchors as these are the portion of the peptide ligand that interact with HLA II. Do the authors draw the same conclusions when only the P1 P4 P6 and P9 anchors are assessed for promiscuity. In addition, the frequency of the HLA II molecules in various populations needs to be included in the analyses. Also, some HLA II have exhibit the four anchors P1 P4 P6 and P9 while others tend to have fewer anchors; how does this impact Pr score? The antibody recognition data of antigens from pathogens is also very selective in regards to the antigens tested, the epitopes from the antigens that were tested, and the linear nature of the epitopes. The neutralizing capacity of these antibodies is unknown as is the affinity of these antibodies for their targets. In regards to the control of infections, the study would be strengthened were high Pr HLA II analyzed with diseases such as influenza, Mtb, COVID, C. difficile, malaria, and dengue - broader antibody recognition might be expected to exacerbate secondary dengue infections and these diseases draw more attention that viral warts. In summary, the study seems to have selected particular data from each of three categories to arrive at the conclusion that HLA II peptide ligand promiscuity is important for controlling infections while omitting a broad spectrum of data that might not support this conclusion.

A few specific comments:

HIV might not be categorized as a newly emerging infectious disease. Additionally, class I HLA has been shown to contribute to the control of HIV infection - are HLA I control alleles in linkage disequilibrium with high Pr HLA II?

HLA alleles are molecular in nature while HLA molecules/antigens/proteins are promiscuous in ligand binding.

(Remarks on code availability)
The data analyzed here were so incomplete that the code is irrelevant.

Reviewer #2

(Remarks to the Author)
In the manuscript entitled "HLA-II peptide-binding diversity shapes humoral immune responses and susceptibility to infections," Magyari and colleagues undertake a comprehensive analysis into the peptide-binding repertoires of allelic variants of HLA class II molecules and correlate their breadth with antibody production. The authors demonstrate that the presence of HLA-II variants with broader peptide-binding repertoires (i.e., high promiscuity) coincides with greater antibody production against a range of common pathogens, with an examination of HLA-typed patient medical records suggesting that this leads to a reduced risk of infection and disease caused by such common pathogens. While promiscuous HLA-II were able to tolerate a range of amino acids at key anchor positions, more fastidious alleles demonstrated high selectivity at

these positions. Through association studies, the authors propose that while promiscuous alleles may protect against common pathogens like HPV, fastidious binders may be more able to respond to emerging diseases such as HIV.

The authors have presented a well-crafted and compelling story, logically progressing from the characterisation of allelic promiscuity and binding motifs, to corresponding antibody production and disease associations. The approach taken by the authors builds on their previous work exploring HLA-I peptide-binding breadth and cancer, where they developed their promiscuity measurements and applied these to patient data in a similar manner. Here, the authors have drawn from previously published datasets and public databases and assessed them anew to generate the data presented here. Overall, the figures are clear and easy to follow, and their schematic workflows allow for a quick understanding of their approach. The writing likewise flows well, although some further explanation in parts (noted below) may help to clarify their work.

- The introduction is succinct and likely sufficient for specialists in the field however a brief explanation of the HLA-II locus (ie. the invariant HLA-DR alpha chain, pairing of alpha and beta chains, and the multiple pairings possible) would make the work more accessible to broader audiences and facilitate their understanding of the subsequent analyses. Likewise, a comment on the typical peptide binding motifs of HLA-II might help (ie. anchor residues at 1, 4, 6, 9), especially noting what the "binding core region" is as it is referred to throughout their analyses. Some of this information could alternatively appear in the relevant results sections.

- The authors could also lean further into the literature to heighten the importance of the study. Notably, their previously reported associations between promiscuity differences in HLA-I and immune checkpoint inhibition outcomes, as well as HLA-II being the strongest genetic factor linked with the presence of antibody-bound antigens, as both provide compelling reasons to pursue this avenue further. These works are referenced throughout the results, but the pertinent details are not mentioned.

- Extended Data 1: The reason to look at subsets ("examine the consistency") in the data is not very clear in the text (is it in case of potential biases within the datasets?)

- Fig 1F/G: The term "peptide core sequence pairs" requires a visit to the methods to interpret. Perhaps it could be rephrased to be more plain (highly divergent "peptide/HLA-II complexes"?) or a more detailed description provided in the main text to help readers understand this (as suggested for the introduction or here).

- Fig 1H/I: Despite the significance and the correlation, the visual spread of the data still suggests a great deal of variation – is one group more significant than another?

- Fig 2: Can the authors please clarify whether this data used the immunopeptidomic database from figure 1. I ask because the methods for generation of the sequence motifs (presumably referring to the logo plots) appear after the analyses of the sequences from the Immune Epitope and Virulence Factor databases.

- Fig 2A: Is there any value in ordering the residues (y axis) based on their maximum "bit" value such that the figure would show promiscuity verses selectivity to show whether there are any patterns to the preferences of fastidious HLA-II (eg. 1R, 1K, 6F, 6D)?

- Fig 2A: note that 'A' is missing on the figure. The colour is hard to see when in greyscale/black and white.

- Fig 2B: At first glance, the logo plots appear deceptive suggesting that HLA-DRB1*16:02 tolerates a wider variety of amino acids compared to DRB1*08:04, which appears more selective (where the reverse is true). Could a logo plot like the Seq2logos that also show a negative y-axis with the under-represented amino acids better emphasise this difference?

- Fig 3A: The text describing the data processing to get from the 1506 associations identified between antibody-bound antigens and HLA-II alleles to the categorisation of 'positive' and 'negative' associations is difficult to follow (in saying that you "focussed on these identified associations" the presence of negative associations doesn't seem possible). The methods help to clarify the how antibody is the dependent variable, but this is just not as clear in the results section text. Can the authors give some numerical details as to the number of HLA-II alleles in the dataset (presumably around 22 or 23 judging by the dots in Figures 3B/C and Extended data figure 4) and the number of positive and negative associations detected for each (which then gets divided into pathogens and non-pathogens, if my understanding is correct – apologies if not!).

- Is there a figure for the pathogens endemic to the Netherlands to go with the Odds Ratio? Was this derived when considering the positive or negative associations or neither?

- Extended data figure 4 is only the LLD cohort – are both cohorts represented in figure 3B/C? At which point was DQ excluded from these analyses? Fig 3D?

- Would the authors predict promiscuous or fastidious HLA-II to better protect from autoimmune diseases? DRB1*15:01 (associated with MS) looks to have relatively low promiscuity… Could there be negative consequences associated with highly promiscuous alleles and unwanted immune responses (beyond susceptibility to emerging diseases)?

The authors have considered multiple variables in their analyses of the UK biobank and LLD/IBD cohorts including age, sex, presence of immunodeficiencies, ancestry, and known HLA associations with given diseases to show that HLA-II promiscuity still significantly shapes antibody production and/or disease risk. The tests applied and resulting significances

are provided for all data. The methods appear comprehensive. Notably, while the original number of samples from the various databases has typically been quoted in the text, the final sample number used for analyses is often located only in the figure legends or methods. Greater transparency as to these final sample numbers in the text would assist in understanding their workflow and analyses.

Overall, this work has the potential to be quite significant to the field through deepening our understanding of, and appreciation for, the consequences of allelic variation among HLA-II molecules. The suggested role of generalist and specialist (promiscuous and fastidious) HLA alleles as being directed towards common or emerging pathogens, respectively, has previously been suggested for HLA-I alleles, but here the authors have applied this concept to HLA-II alleles (which are probably the lesser studied class in terms of polymorphism), and have further provided data to support this hypothesis. Importantly, the authors skilfully link this HLA-II promiscuity to antibody production and to the risk of disease, suggesting that peptide-binding repertoires can have broad impacts on multiple aspects of the immune system, thus making the work relevant and of interest across many fields.

(Remarks on code availability)
I am not in a position to review code.

Version 1:

Reviewer comments:

Reviewer #2

(Remarks to the Author)
In revising their manuscript, “HLA-II peptide-binding diversity shapes humoural immune response and susceptibility to infections,” Magyari and colleagues have endeavoured to address the comments raised by both reviewers, where appropriate, with the addition of new data and supplemental information.

The additional details regarding HLA-II structure, peptide anchor residues, “core” regions and previous genetic associations with antibody repertoire are helpful and should broaden the accessibility and enhance the importance of the topic to readers. Clarity has also been improved where sought. The authors have considered recalculations and data presentation in response to the comments. The revised heatmap (Fig. 2A) is easier to read with the impact of certain residues now more obvious (although I do wonder whether the addition of axis labels for ‘increasing promiscuity’ and ‘maximum specificity’ could help further improve the readability of the figure). The revised logo plots (Fig. 2B) including all amino acids and new Fig C are clear. The definition of positive and negative associations (line 216-218), along with the addition of Extended Data Table 1, makes the authors’ data crunching and subsequent data presentation more transparent and easier to follow.

In response to Reviewer 1’s comments the authors have also included additional analyses from an influenza vaccination cohort, which provides a more controlled setting within which to assess the relationship between HLA-II promiscuity and functional antibody responses (as opposed to the presence of antibodies against linear epitopes identified in the PhIP-Seq databases). Promiscuity was only found to correlate with neutralising antibody/HAI titres in one of three age ranges, making this a thought-provoking add-on. Was HLA-DRB1 promiscuity for the donors in the Fluzone vaccine trial calculated as for those in the UK biobank – ie. calculated by averaging the allele-specific values of the b chains encoded by the HLA-DRB1 gene? This does not appear to be specified in the methods. Presumably all participants had sequencing.

Overall, the authors have considered each of this reviewer’s comments and addressed them sufficiently.

(Remarks on code availability)
I am not in a position to review code.

Reviewer #3

(Remarks to the Author)
1) Reviewer 1’s major criticism, expressed in several places, is that the authors appear to have selectively presented data that support their conclusions:

“A primary concern is that the three segments of the study appear to have been completed in a manner that selectively support the contribution of promiscuity towards controlling infection.””

“The antibody recognition data of antigens from pathogens is also very selective in regards to the antigens tested, the epitopes from the antigens that were tested, and the linear nature of the epitopes”

“”In regards to the control of infections, the study would be strengthened were high Pr HLA II analyzed with diseases such as influenza, Mtb, COVID, C. difficile, malaria, and dengue - broader antibody recognition might be expected to exacerbate secondary dengue infections and these diseases draw more attention that viral warts”

The authors disagree with this characterization:

“We disagree with the implication of confirmation bias. Our dataset selections were driven by availability, scale, and relevance—not by outcome.”

“Our study was explicitly designed to minimize bias and maximize representativeness at every level. … Contrary to the reviewer's concerns, implementing the suggested changes (focusing only on anchoring positions when calculating promiscuity; selecting alleles based on population prevalence; focusing only on certain disorders, etc.) would in fact increase selectivity and potential bias, undermining the strength of our comprehensive approach.”

In my opinion the study is not biased in the way that Reviewer 1 suggests, and the authors’ rebuttals generally are convincing. Reviewer 1 seems concerned that only selected pathogens and disease associations are presented, while others that do not align with the conclusion are ignored. The authors appear to have conducted an unbiased analysis with careful consideration of statistical issues such as multiple comparisons, and have presented only the associations resulting from this analysis that are statistically significant. However, this is not clearly stated in the manuscript, and in many places that manuscript is written in such a way as to suggest that the “cherry-picking” concerning reviewer 1 might have occurred. These ambiguities have not been addressed in the revised manuscript and might be source of misunderstanding as the authors suggest.

For example:
“Notably, for many positive associations, we found that HLA-II molecules exhibit a distinct ability to present specific segments of antigens, underscoring the complex relationship between HLA-II alleles and immune recognition (lines 220-223, emphasis added).”

“After adjusting for multiple hypothesis testing, we identified eight disease groups whose occurrence decreased with increased HLA promiscuity (Figure 4D). These associations were particularly strong for viral and bacterial skin infections, sexually transmitted diseases (dominated by genital warts), lower respiratory tract infections, and mycoses. (lines 309-313,emphasis added)”

“Additionally, this protective effect was significant for viral warts of the skin, also caused by HPV, with a 15.7% reduced risk. Similar reductions were observed for herpes zoster (14.3% reduced risk), caused by the varicella-zoster virus, and for local skin and soft tissue infections, primarily bacterial in origin (14.1% reduced risk) (lines 317-320, emphasis added)”

:Higher HLA-DRB1 promiscuity decreases the lifetime incidence of multiple infections. The table summarizes the Cox proportional hazard models on the incidence of various disorders, treating HLA- DRB1 promiscuity as a continuous variable. (lines 389-391, legend to Figure 4D, emphasis added)”

In each case no rationale was presented for selection of the associations, disease groups, protective effects, or multiple infections highlighted. Examination of the table presented in Figure 4D suggests that rather than cherry picking the authors seem to have considered all of the conditions in the entire analysis, ranked them by their hazard ratio, and selected only those with false discovery rates < 0.05. If this is correct that should be clearly stated in manuscript. If not, the authors should in each case provide the rationale for selection of the particular pathogens, diseases, etc highlighted.

A conventional presentation of this sort of data is a volcano plot, where the reader could see for themselves all of the relevant data together with effect size and uncertainly values, and how any selected items fit into the overall distribution. The authors might consider adding such a plot, where the Cox HR value would take the place of the conventional “fold increase” axis and the FDR take the place of the conventional p-value axis.

2) In a similar vein, Reviewer 1 notes that the authors are not very systematic or precise in how they describe the main results of the study:

In summary, the study seems to have selected particular data from each of three categories to arrive at the conclusion that HLA II peptide ligand promiscuity is important for controlling infections while omitting a broad spectrum of data that might not support this conclusion.”

“HIV might not be categorized as a newly emerging infectious disease.”

Indeed, the main conclusion of the study is stated clearly in the title of the manuscript:

“HLA-II peptide-binding diversity shapes humoral immune responses and susceptibility to infections” (lines 1-2)
but in the abstract the association between HLA-II binding diversity and susceptibility to infections has been extended to overall health outcomes:

“demonstrating that a broad peptide-binding repertoire of HLA-II is linked to antibody production against pathogen-associated proteins and more favourable health outcomes (lines 27-27).

What actually was observed is that the breadth of the HLA-II peptide binding repertoire was positively associated with humoral responses to some pathogens but not others, and was positively associated with some positive health outcomes but not significantly associated with most of the positive health outcomes investigated. This is clearly stated in the first

paragraph of the discussion:

Individuals carrying HLA-II variants with a broad peptide-binding repertoire are more likely to produce antibodies that target pathogen-associated proteins and are less likely to experience severe infections from common pathogens, as reflected in their medical records. Additionally, high HLA-II promiscuity was associated with a lower cumulative incidence of a wide range of infections. (lines 423-427)

In fact, the authors call out a HIV as a prominent exception to the positive health association:

Notably, we found that the low promiscuity HLA-II variants provide protection against HIV, one of the most relevant emerging viruses (lines 444-445, also Fig 4G)

Obviously, protection against HIV would be considered a favorable health outcome, but here the association is opposite the statement in the abstract. The authors should take to care to summarize their findings carefully and avoid broad generalizations beyond what is demonstrated by their data and careful statistical analysis.

Reviewer 1 also notes that “HIV might not be categorized as a newly emerging infectious disease”

apparently in regard to the explanations provided for the HIV outlying results:

On the other hand, HLA-II molecules with a low promiscuity level may specifically target emerging, but relatively rare pathogens (31) (line 221-223).

However, this does not imply that low-promiscuity HLA alleles are without benefit. These alleles may play a critical role in defending against emerging pathogens (lines 327-328)

Notably, we found that the low promiscuity HLA-II variants provide protection against HIV, one of the most relevant emerging viruses, which is in line with previous results on HLA-I (45). (lines 450-452)

In fact, unlike HIV several other emerging diseases such as Dengue, Ebola, human alphaviruses, SARS, MRSA, and West Nile virus were not found to be negatively associated with HLA-II binding promiscuity (Extended Data Table 2). A conclusion about emerging viruses would have been warranted if the authors had performed a specific test of emerging versus established viruses and observed a significant association, but in this absence there is no data presented that supports any conclusion about recently emerging viruses. The authors could just have easily characterized the result as pertaining to small positive strand RNA viruses, or T-cell tropic viruses, or chimp-derived zoonotic viruses.

A statement that HLA-II peptide binding diversity is associated with protective humoral responses against some but not all viruses investigated, with the specific mechanisms to be determined, would be a more appropriate characterization of the results than any conclusions about recent versus past viral emergence into humans.

3) Another concern of reviewer 1 is the use of the full nine-residue peptide binding preferences instead of focusing on the major MHC-peptide contact positions:

“It would have been helpful to have the study focused on P1 P4 P6 and P9 anchors as these are the portion of the peptide ligand that interact with HLA II. Do the authors draw the same conclusions when only the P1 P4 P6 and P9 anchors are assessed for promiscuity”

The authors have addressed this concern clearly in the revised manuscript, rationalizing the use of the full 9-residue motif, and providing in the rebuttal new data that an analysis restricted to P1,4,6,9 yields similar results.

However, the authors might consider addressing a related issue not specifically mentioned by reviewer 1. In their analysis of HLA-II peptide binding preferences the authors seem to imply that they are providing new information about the ability of various HLA-II molecules to bind diverse peptide sequences:

“To assess the robustness of the Pr values and minimize potential bias arising from differences in experimental setups across immunopeptidomics studies, we re-calculated Pr using two independent subsets of the original dataset (n = 102 and n = 21 samples). (lines 112-115).”

“The reliability of the calculated Pr values was further validated using data from two independent immunopeptidomics studies, which focused on HLA-II-presented peptides from adeno-associated virus (AAV) and SARS-CoV-2 (22, 23). (lines 119-121).”

In every case the calculated Pr values were determined for lists of peptides derived from previously-reported large immunopeptidomics datasets characterizing multiallelic samples. In these lists the peptides were assigned, either in the original study (ref 22) or retrospectively by the authors (ref 23), to the individual HLA alleles present in each sample using a NetMHCIIpan, a neural-network based MHC-peptide binding prediction algorithm. Thus the diversity profiles for each allele are determined largely by the prediction algorithm. Moreover, the authors only consider HLA-DRB1 alleles, and not the

genetically linked HLA-DRB3, HLA-DRB4, and HLA-DRB5 allele which are present in most samples. These alleles have different specificities than the genetically linked HLA-DRB1 alleles (Kaabinejadian et al Front Immunol PMID:35154160), and the authors' comingling of their peptidomes introduces error into the calculated Pr values. Datasets derived from monoalleleic samples are available (Abelin et al Immunity 2019 PMID: 31495665) and would avoid these problems, but these were not utilized. The authors should consider mentioning in the limitation section of the manuscript that the Pr values depend on HLA assignments derived from the allele assignment algorithm NetMHCIIpan.

4) Another concern of reviewer 1 is that the original study was based on the phage display platform PhIP-Seq, which might not reflect biologically relevant antibody binding:

"The antibody recognition data of antigens from pathogens is also very selective in regards to the antigens tested, the epitopes from the antigens that were tested, and the linear nature of the epitopes. The neutralizing capacity of these antibodies is unknown as is the affinity of these antibodies for their targets."

The authors helpfully respond by extending the analysis to an existing dataset of neutralizing antibodies induced by influenza vaccination, showing the same allele promiscuity effects as the PhIP-Seq analysis. As part of this analysis the authors uncovered an interesting age-dependent change in the association of HLA-II peptide binding promiscuity with neutralizing antibody titer. The authors might be interested in a very recent paper using PhIP-Seq to analyze anti-influenza responses by age (Olin et al Nat Immunol 2026 PMID: 41699059), which also observed age-dependent changes in epitope specificity (although not addressing HLA-II binding diversity as in the present work).

Other more minor concerns of reviewer 1 were addressed clearly in the rebuttal and/or revised manuscript.

(Remarks on code availability)

Version 2:

Reviewer comments:

Reviewer #2

(Remarks to the Author)
In the next revision of their manuscript, "HLA-II peptide-binding diversity shapes humoral immune response and susceptibility to infections," Magyari and colleges have worked to address the additional comments from reviewers with clarification of the text and some figure adjustments.

The comments from reviewer two have been sufficiently addressed through minor adjustments of a figure and the text.

Reviewer three has carefully assessed the comments raised by reviewer one and the authors have endeavoured to address them. To dispel concerns regarding the "cherry-picking" of data, the authors have softly adjusted the text to demonstrate that their examples are just that, examples (eg. Use of the word "including"). However, there is no distinct rationale for why A63, B02, B07 and L08 have been analysed in Fig4E (which I think is what reviewer three was after). The addition of the suggested volcano plot provides a welcome visualisation of the impact of promiscuity on infection, and one can see that the four groups selected for the following analyses are the most significant, but maybe that just needs to be said in the text.

The authors have also modified their characterisation of HIV as an "emerging" virus, now referring to it as evolutionarily novel, thus preventing generalisations about emerging viruses and HLA-II promiscuity from being made. The adjustments to the abstract, considering that HIV is an exception to the benefits of promiscuity found in some other infections as flagged by the reviewer (now stating favourable outcomes for "several common infections" instead of "health outcomes") are appropriate and more accurate.

The authors have added subheadings to their discussion, including a section on limitations where they have made note of the biases of the predictive allele-based assignments of peptides (as suggested by the reviewer), although they do not make mention of DRB3, 4 or 5 alleles that have not been represented in their dataset. They have also added reference to the influenza paper suggested by the reviewer.

Overall, the authors have addressed the concerns of each of the reviewers throughout the various iterations of the manuscript.

(Remarks on code availability)
I am not in a position to review code.

Reviewer #3

(Remarks to the Author)
The revised manuscript addresses each of the four issues brought up in my review (and initially noted in reviewer 1's comments on the original manuscript).

(Remarks on code availability)

Open Access This Peer Review File is licensed under a Creative Commons Attribution 4.0 International License, which permits use, sharing, adaptation, distribution and reproduction in any medium or format, as long as you give appropriate credit to the original author(s) and the source, provide a link to the Creative Commons license, and indicate if changes were made.
In cases where reviewers are anonymous, credit should be given to 'Anonymous Referee' and the source.
The images or other third party material in this Peer Review File are included in the article's Creative Commons license, unless indicated otherwise in a credit line to the material. If material is not included in the article's Creative Commons license and your intended use is not permitted by statutory regulation or exceeds the permitted use, you will need to obtain permission directly from the copyright holder.
To view a copy of this license, visit https://creativecommons.org/licenses/by/4.0/

Response to Reviewer 1

We appreciate the time and constructive feedback provided by Reviewer 1; however, we respectfully disagree with several of the points raised. We believe that some of these concerns stem from misunderstandings regarding our methodology and data sources.

"Magyari et al evaluate class II HLA molecules according to their peptide presentation characteristics, assigning a promiscuity index to individual class II HLA molecules according to the amino acid composition of their peptide ligands. The study then segues to test whether HLA II with higher Pr are likely to bind more pathogen derived peptides. Lastly, the study links HLA II promiscuity to the presence of antibodies to pathogens and the control of infectious disease. The study concludes/suggests that promiscuous HLA II molecules protect from a variety of infections while low promiscuity HLA II defend against emerging pathogens. A primary concern is that the three segments of the study appear to have been completed in a manner that selectively support the contribution of promiscuity towards controlling infection."

We disagree with the implication of confirmation bias. Our dataset selections were driven by availability, scale, and relevance—not by outcome. The complete response is provided on page 6 of this document.

"It would have been helpful to have the study focused on P1 P4 P6 and P9 anchors as these are the portion of the peptide ligand that interact with HLA II. Do the authors draw the same conclusions when only the P1 P4 P6 and P9 anchors are assessed for promiscuity. In addition, the frequency of the HLA II molecules in various populations needs to be included in the analyses. Also, some HLA II have exhibit the four anchors P1 P4 P6 and P9 while others tend to have fewer anchors; how does this impact Pr score?"

We had not explicitly highlighted this point, but anchor positions are inherently incorporated into our promiscuity metric. By definition, anchor positions exhibit a more restricted amino acid distribution due to their structural and functional constraints in peptide-MHC binding. This restricted distribution naturally leads to higher Kullback-Leibler divergence values at these positions, thereby contributing to lower promiscuity scores. This relationship is quantitatively supported by the high correlation ($r = 0.92$) between promiscuity values calculated using all 9-mer positions versus only the anchor positions.

<u>We have modified the manuscript as follows to explain this aspect more clearly:</u>

"Notably, our promiscuity metric, which is computed from amino acid distributions across all positions of 9-mer peptides, inherently assigns greater weight to anchor residues: their structurally constrained, restricted amino acid usage produces higher Kullback–Leibler divergence values and thus disproportionately influences the overall promiscuity score. Consistent with this, promiscuity values based on all positions are highly correlated with those calculated from anchor positions only (Spearman's rho = 0.92, p value of two-sided correlation test $< 2.2x10^{-16}$)."

The suggestion to incorporate HLA allele frequencies is a similar misunderstanding of the statistical framework of our analysis, that we maybe did not explain well enough: HLA-II associations are more readily detected for common alleles due to increased statistical power, meaning that these frequencies are already implicitly accounted for in the data. This phenomenon is not only general but also demonstrably present in the UK Biobank dataset, which we used. Furthermore, our selected HLA allele set encompasses the most common HLA-II alleles, ensuring robust and representative coverage. In the original version of the manuscript, we wrote the following paragraphs explaining this issue – we are happy to extend them with any additional suggestions:

“To evaluate the prevalence of these alleles across human populations, we used a comprehensive reference list of HLA-II alleles that cover a large fraction (>99%) of the individuals in human populations (17). Encouragingly, we could determine the promiscuity index for 89% of these alleles. Moreover, our allele set with Pr values involved all nine HLA-DRB1 alleles identified as universally frequent or very frequent in human populations (18).”

“We successfully determined HLA-DRB1 promiscuity levels for over 96% of participants in the UK Biobank.”

“The antibody recognition data of antigens from pathogens is also very selective in regards to the antigens tested, the epitopes from the antigens that were tested, and the linear nature of the epitopes. The neutralizing capacity of these antibodies is unknown as is the affinity of these antibodies for their targets.“

While we fully acknowledge the critical role of conformational epitopes in immune recognition, their inclusion in large-scale analyses remains limited by the lack of comprehensive and scalable experimental assays. Nonetheless, PhIP-seq offers the most extensive and systematic dataset of linear epitopes currently available, making it the most practical and reliable proxy for high-throughput analysis. The epitopes analyzed in our study are curated from authoritative and well-established resources, including the Immune Epitope Database (Vita el al., Nucleic Acids Res., 2025) and the Virulence Factor Database (Zhou et al, Nucleic Acids Res., 2025), reinforcing both the reliability and biological relevance of the dataset. Importantly, the same HLA class II–restricted CD4+ T cell help that supports responses to linear peptides is also essential for the affinity maturation of B cells targeting conformational epitopes. Thus, although our approach is restricted to peptides of 54 to 64 aa, it nonetheless captures key immunological processes that contribute to antibody maturation against conformational targets. Regarding the neutralizing activity of antibodies, and following the reviewer’s later suggestion, we have incorporated into the revised manuscript a case study on influenza vaccination, in which we show that HLA-II promiscuity correlates with the induction of neutralizing antibodies (see page 7).

<u>We have modified the manuscript as follows:</u>

“PhIP-seq currently provides the most extensive and systematic dataset of linear epitopes, making it a practical and reliable proxy for high-throughput analysis. The epitopes analyzed here were curated from established resources, including the Immune Epitope Database (IEDB) and the Virulence Factor Database (see refs. (25, 26) for details).”

*“Moreover, many antigens in these associations are established targets of neutralizing antibodies. For example, antibodies against Streptolysin O, one of the best-characterized S. pyogenes antigens (28, 29), were negatively associated with HLA-DQA1*03:01.”*

“As outlined elsewhere (47), PhIP-Seq is intrinsically limited by peptide length, which captures linear but may miss conformational epitopes that require folding of larger protein regions. The proportion of linear versus conformational epitopes recognized by human antibodies is unknown and difficult to measure; however, even if our approach detected only 10% of antibody–antigen interactions against bacteria, the tens of thousands of proteins in our library would still provide a throughput exceeding current ELISA or peptide array methods by about an order of magnitude. We have shown that phage-displayed peptides can reconstitute expected protein functions requiring at least secondary structure (47). Furthermore, our work (48) and that of others (35, 49, 50) using full-length proteins suggests similar HLA-II–dependent mechanisms in the selection and maturation of B cells targeting conformational epitopes.”

“A primary concern is that the three segments of the study appear to have been completed in a manner that selectively support the contribution of promiscuity towards controlling infection.”

”In regards to the control of infections, the study would be strengthened were high Pr HLA II analyzed with diseases such as influenza, Mtb, COVID, C. difficile, malaria, and dengue - broader antibody recognition might be expected to exacerbate secondary dengue infections and these diseases draw more attention that viral warts. In summary, the study seems to have selected particular data from each of three categories to arrive at the conclusion that HLA II peptide ligand promiscuity is important for controlling infections while omitting a broad spectrum of data that might not support this conclusion.”

Our study was explicitly designed to minimize bias and maximize representativeness at every level. We employed:

- A non-selective inclusion of HLA alleles using the most comprehensive immunopeptidomics dataset available;
- A non-biased antibody dataset selection using the largest high-quality classification of pathogens, non-pathogens, and common infections;
- And a population-scale, non-selective use of UK Biobank data, which offers unparalleled breadth and depth.

Contrary to the reviewer's concerns, implementing the suggested changes (focusing only on anchoring positions when calculating promiscuity; selecting alleles based on population prevalence; focusing only on certain disorders, etc.) would in fact increase selectivity and potential bias, undermining the strength of our comprehensive approach.

To avoid future misunderstandings, we included an explicit discussion in the revised manuscript addressing the generalizability and representativeness of our study design:

“In our study, we aimed to minimise selection bias and maximise generalisability across pathogens and HLA class II alleles. We based our analyses on one of the most comprehensive immunopeptidomics datasets currently available, applying a non-selective inclusion of HLA alleles and a large, high-quality antibody dataset encompassing pathogens, non-pathogens, and common infections. In parallel, we leveraged UK Biobank at population scale without focusing on a specific disease or certain alleles, thereby ensuring that our estimates of HLA class II promiscuity are not driven by a few highly studied conditions or population-specific allelic distributions.”

“...these diseases draw more attention that viral warts”

Already in the initial manuscript we had reported several distinct conditions beyond viral warts, such as pneumonia and HIV—which clearly have significant clinical and epidemiological importance. In the original manuscript, we wrote:

“These associations were particularly strong for viral and bacterial skin infections, sexually transmitted diseases (dominated by genital warts), lower respiratory tract infections, and mycoses.”

In the revised manuscript, we discuss the importance of these diseases further:

“Additionally, high HLA-II promiscuity was associated with a lower cumulative incidence of a wide range of infections. The consistent protective effects observed for both mucosal and cutaneous HPV indicate that this advantage is not tissue-restricted but extends across distinct epithelial niches. Local bacterial infections of the skin and subcutaneous tissues (e.g., cellulitis) are typically not attributed to host genetics, yet we found that promiscuity also confers

protection in these extracellular bacterial settings, suggesting a broad immune benefit. For herpes zoster, previous GWAS have implicated specific alleles in shingles risk (43), whereas our results show that overall binding breadth, rather than any single allele, predicts protection, underscoring the importance of antigenic diversity in controlling latent virus reactivation. Similarly, candidiasis—a hallmark of impaired T-cell immunity—highlights how presentation breadth shapes susceptibility to opportunistic pathogens. Pneumonia and other lower respiratory tract infections remain among the leading causes of infectious mortality worldwide (44), and the observation that HLA promiscuity is protective in this context further broadens its clinical relevance."

To further strengthen our conclusions and align with the reviewer's suggestions, we present a focused case study on influenza vaccination response in the revised manuscript. Our findings illustrate the mechanistic role of HLA-II promiscuity in vaccine response. In the revised manuscript, we write:

"HLA-II promiscuity level influences response to influenza vaccination in an age-dependent manner

Although we detected an effect of HLA-II promiscuity in eight disease groups, we did not observe an association for several common infections, which could arise from multiple factors. First, susceptibility may be primarily determined by immune mechanisms other than HLA-II presentation. Second, the UK Biobank cohort is highly heterogeneous, leading to substantial variation in exposure to infectious agents. Third, vaccination coverage, timing, and vaccine type differ between individuals. Fourth, both susceptibility to and clinical manifestation of many infections are strongly age-dependent, which may mask the effects of HLA-II promiscuity.

To reduce these confounding factors, we next focused on an influenza vaccination cohort (40). In this study, healthy individuals from different age groups received the same inactivated vaccine Fluzone according to a standardized protocol (Figure 5A). This design equalizes antigen exposure (dose, formulation, and timing), thereby removing variability in contact with the virus and in vaccination history. Moreover, because vaccine-induced antibody responses strongly depend on $CD4^+$ T cell help, and thus on HLA-II peptide presentation, a vaccination study provides a particularly suitable setting to assess the impact of HLA-II promiscuity, including its age-dependent effects.

We calculated HLA-DRB1 promiscuity in individuals from three age groups (18–30, 60–79, and 80–90 years) and related it to functional antibody responses to influenza, measured by hemagglutination inhibition (HAI) assays. Vaccine responses were defined as the fold-change in HAI titer from baseline (day 0) to day 28. HLA-II promiscuity showed a strong positive correlation with antibody induction in the 60–79-year group, but not in the 18–30 or 80–90-year groups (Figure 5B). This pattern was corroborated by the virus neutralization assay, which revealed the same association in the 60–79-year group but not in the other age groups (Figure 5C).

Together, these data indicate that HLA-II promiscuity is a robust determinant of influenza vaccine responsiveness specifically in older adults. They further suggest that there is an age window in which broader peptide presentation by HLA-II molecules can be translated into superior antibody responses, before advanced immunosenescence overrides this effect. This age-dependent impact of HLA-II promiscuity has important implications for understanding inter-individual variation in vaccine efficacy and for optimizing vaccination strategies in the elderly.

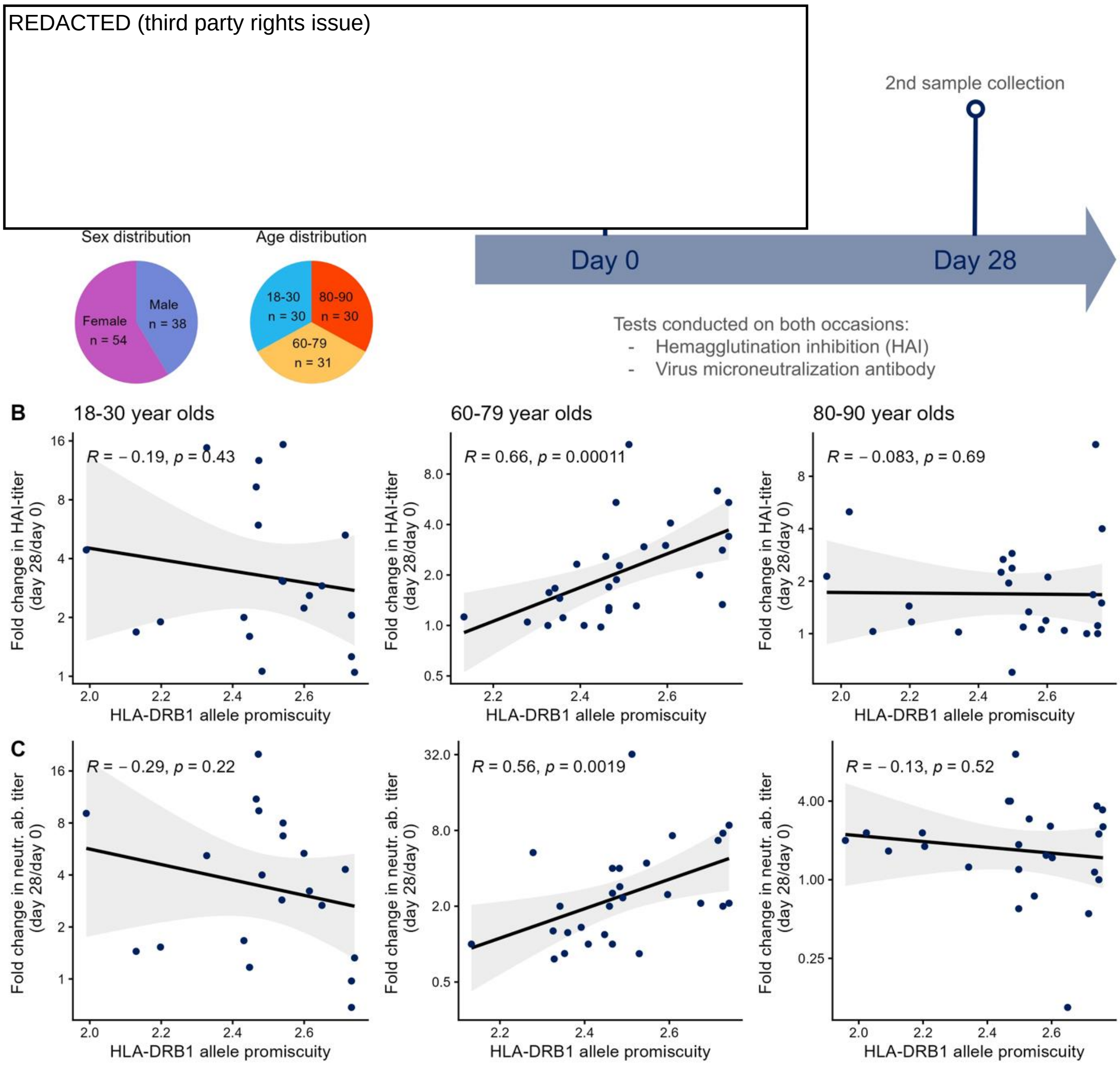

Figure 5. High HLA-II promiscuity is associated with a stronger response to the Fluzone vaccine in the 60–79-year age group. A) *Overview of the study design and descriptive statistics of the study cohort.* ***B-C)*** *Fold change in hemagglutination inhibition (HAI) titer (**B**) and neutralizing antibody titer (**C**) from day 0 to day 28 as a function of HLA-DRB1 promiscuity in the three age groups. Reported titer values represent the average of the individual strain-specific titers. Spearman's rho values and p values of two-sided correlation tests are indicated, and vertical axes are log_2-transformed."*

"*HIV might not be categorized as a newly emerging infectious disease. Additionally, class I HLA has been shown to contribute to the control of HIV infection - are HLA I control alleles in linkage disequilibrium with high Pr HLA II?****"***

HIV is widely recognized as an evolutionarily recent virus in the context of human-pathogen co-evolution (compared to e.g. herpesviruses that have co-evolved for millions of years), as supported by multiple peer-reviewed studies (Morens et al., Nature, 2004; Jones et al., Nature, 2008; Morens et al., PLoS Pathogens, 2013). This characterization provides relevant context for our findings and supports the interpretations presented in our manuscript. Regarding the second part of the comment, we accounted for the prevalence of HIV-associated HLA-I alleles in our analysis, and the effect of HLA-II promiscuity remained significant (see Extended Data Table 8 in the original manuscript). This suggests that the observed association is independent of any confounding influence from these HLA-I alleles. In the revised manuscript, we write:

"To explore this, we analyzed the cumulative incidence of HIV infection. HIV is an evolutionarily recent human pathogen with substantial case numbers in the UK Biobank. (36) HIV-1, a retrovirus in the kingdom Pararnavirae, lies on a deeply divergent branch of the RNA virus phylogeny relative to Orthornavirae viruses that cause most common human infections (37) and is therefore expected to have a highly distinct antigenic repertoire (38, 39)"

"HLA alleles are molecular in nature while HLA molecules/antigens/proteins are promiscuous in ligand binding."

In line with previous studies (Chowell et al., Science, 2017; Marty et al., Cell, 2017), we use the terms *HLA allele* and *HLA molecule* interchangeably, and we now state this explicitly in the revised manuscript:

"First, we aimed to determine the promiscuity level of different HLA-II variants by evaluating the diversity of peptides each allele can bind. Unless otherwise specified, we use HLA alleles as shorthand for their corresponding HLA proteins in line with previous studies (17, 18)."

"Reviewer #1 (Remarks on code availability):
The data analyzed here were so incomplete that the code is irrelevant."

Please see our response above on page 6.

3. Response to Reviewer 2

„In the manuscript entitled "HLA-II peptide-binding diversity shapes humoral immune responses and susceptibility to infections," Magyari and colleagues undertake a comprehensive analysis into the peptide-binding repertoires of allelic variants of HLA class II molecules and correlate their breadth with antibody production. The authors demonstrate that the presence of HLA-II variants with broader peptide-binding repertoires (i.e., high promiscuity) coincides with greater antibody production against a range of common pathogens, with an examination of HLA-typed patient medical records suggesting that this leads to a reduced risk of infection and disease caused by such common pathogens. While promiscuous HLA-II were able to tolerate a range of amino acids at key anchor positions, more fastidious alleles demonstrated high selectivity at these positions. Through association studies, the authors propose that while promiscuous alleles may protect against common pathogens like HPV, fastidious binders may be more able to respond to emerging diseases such as HIV.

The authors have presented a well-crafted and compelling story, logically progressing from the characterisation of allelic promiscuity and binding motifs, to corresponding antibody production and disease associations. The approach taken by the authors builds on their previous work exploring HLA-I peptide-binding breadth and cancer, where they developed their promiscuity measurements and applied these to patient data in a similar manner. Here, the authors have drawn from previously published datasets and public databases and assessed them anew to generate the data presented here. Overall, the figures are clear and easy to follow, and their schematic workflows allow for a quick understanding of their approach. The writing likewise flows well, although some further explanation in parts (noted below) may help to clarify their work."

We thank the reviewer for their insightful comments and truly appreciate the positive feedback; we have endeavoured to respond to each point in detail.

„The introduction is succinct and likely sufficient for specialists in the field however a brief explanation of the HLA-II locus (ie. the invariant HLA-DR alpha chain, pairing of alpha and beta chains, and the multiple pairings possible) would make the work more accessible to broader audiences and facilitate their understanding of the subsequent analyses. Likewise, a comment on the typical peptide binding motifs of HLA-II might help (ie. anchor residues at 1, 4, 6, 9), especially noting what the "binding core region" is as it is referred to throughout their analyses. Some of this information could alternatively appear in the relevant results sections."

Thank you for the suggestion. We have revised the text and incorporated the suggested details to make it more accessible to a broader audience. The updated text is as follows:

„First, we aimed to determine the promiscuity level of different HLA-II variants by evaluating the diversity of peptides each allele can bind. Unless otherwise specified, we use HLA alleles as shorthand for their corresponding HLA proteins in line with previous studies (17, 18). HLA-II molecules predominantly bind peptides of 12–20 amino acids in length (19). A nine–amino acid core segment sits in the binding groove, while flanking residues extend beyond it. We utilized the data from a recent immunopeptidomics study which includes details on 615,361 unique peptides identified across 322 samples, including the corresponding core segment for each peptide."

„Alleles with high Pr values can bind a broader range of peptides. Note that for HLA-DP and -DQ molecules, both the α- and β-chains are polymorphic, whereas for HLA-DRB1 the α-chain is essentially invariant and peptide binding is determined solely by the β-chain. Accordingly, as in other studies (19), when we refer to "alleles" we mean α–β chain combinations for DP and DQ, but β-chain variants only for DRB1."

„The binding motifs capture amino acid preferences within the binding core region, with the anchor positions (typically 1, 4, 6, and 9) showing the strongest specificities (19)."

„The authors could also lean further into the literature to heighten the importance of the study. Notably, their previously reported associations between promiscuity differences in HLA-I and immune checkpoint inhibition outcomes, as well as HLA-II being the strongest genetic factor linked with the presence of antibody-bound antigens, as both provide compelling reasons to pursue this avenue further. These works are referenced throughout the results, but the pertinent details are not mentioned."

Thank you for the comment. We modified the text as follows:

„Previously, we showed that variation in the HLA-II region is the strongest genetic determinant of the antibody repertoire (4)."

„Our previous work showed that HLA-I promiscuity strongly influences the efficacy of immune checkpoint blockade therapy (15). Building on these findings, examining HLA-II promiscuity may likewise reveal an important role in shaping antitumor immune responses."

„Extended Data 1: The reason to look at subsets ("examine the consistency") in the data is not very clear in the text (is it in case of potential biases within the datasets?)"

Thank you for raising this point. We clarified this in the revised manuscript:

*„To assess the robustness of the Pr values and minimize potential bias arising from differences in experimental setups across immunopeptidomics studies, we re-calculated Pr using two independent subsets of the original dataset (n = 102 and n = 21 samples). Although the samples originated from different sources, all raw data were processed with the same bioinformatics pipeline (19). The Pr values derived from the two subsets showed a strong correlation (**Extended Data Fig. 1**), indicating that our estimates are consistent and not driven by study-specific experimental conditions."*

„Fig 1F/G: The term "peptide core sequence pairs" requires a visit to the methods to interpret. Perhaps it could be rephrased to be more plain (highly divergent "peptide/HLA-II complexes"?) or a more detailed description provided in the main text to help readers understand this (as suggested for the introduction or here)."

As suggested, we provided a more detailed description in the main text:

„HLA-II molecules predominantly bind peptides of 12–20 amino acids in length (19). A nine–amino acid core segment sits in the binding groove, while flanking residues extend beyond it."

„Fig 1H/I: Despite the significance and the correlation, the visual spread of the data still suggests a great deal of variation – is one group more significant than another?"

Thank you for this observation. We recalculated the correlation coefficients separately for the three molecules and obtained similar values:

- **IEDB** – DP: 0.34 (p = 0.17, n = 18), DQ: 0.40 (p = 0.14, n = 15), DRB1: 0.47 (p = 0.003, n = 36)
- **VFDB** – DP: 0.36 (p = 0.14, n = 18), DQ: 0.35 (p = 0.20, n = 15), DRB1: 0.47 (p = 0.004, n = 36)

The weaker p values for DP and DQ likely reflect the smaller number of alleles included in these analyses. In addition, the slightly lower correlation coefficients for DP and DQ may be due to the lower prediction accuracy of the NetMHCIIpan algorithm for these molecules compared with DRB1.

„Fig 2: Can the authors please clarify whether this data used the immunopeptidomic database from figure 1. I ask because the methods for generation of the sequence motifs (presumably referring to the logo plots) appear after the analyses of the sequences from the Immune Epitope and Virulence Factor databases."

Thank you for raising this point, we clarified this in the revised manuscript as follows:

„To generate the binding motifs, we used the same dataset as for calculating the Pr values."

"Fig 2A: Is there any value in ordering the residues (y axis) based on their maximum "bit" value such that the figure would show promiscuity verses selectivity to show whether there are any patterns to the preferences of fastidious HLA-II (eg. 1R, 1K, 6F, 6D)?"

Thank you for the suggestion. We have revised the heatmap, which now more clearly shows which amino acids at specific positions are enriched in fastidious HLA-II molecules (see Figure below).

"Fig 2A: note that 'A' is missing on the figure. The colour is hard to see when in greyscale/black and white."

We have inserted "A" and modified the colour palette according to your suggestion (see Figure below).

"Fig 2B: At first glance, the logo plots appear deceptive suggesting that HLA-DRB116:02 tolerates a wider variety of amino acids compared to DRB108:04, which appears more selective (where the reverse is true). Could a logo plot like the Seq2logos that also show a negative y-axis with the under-represented amino acids better emphasise this difference?"

Unfortunately, Kullback–Leibler–based motifs, which highlight under-represented amino acids, were not suitable here because the negative component dominated the logos (see below) given the characteristics of our data. In the revised manuscript, we therefore improved the logo plots by displaying all amino acids at every position and added an additional plot showing the difference between observed and background amino acid frequencies (i.e., components of the Kullback-Leibler logos) at all binding-site positions for the two alleles.

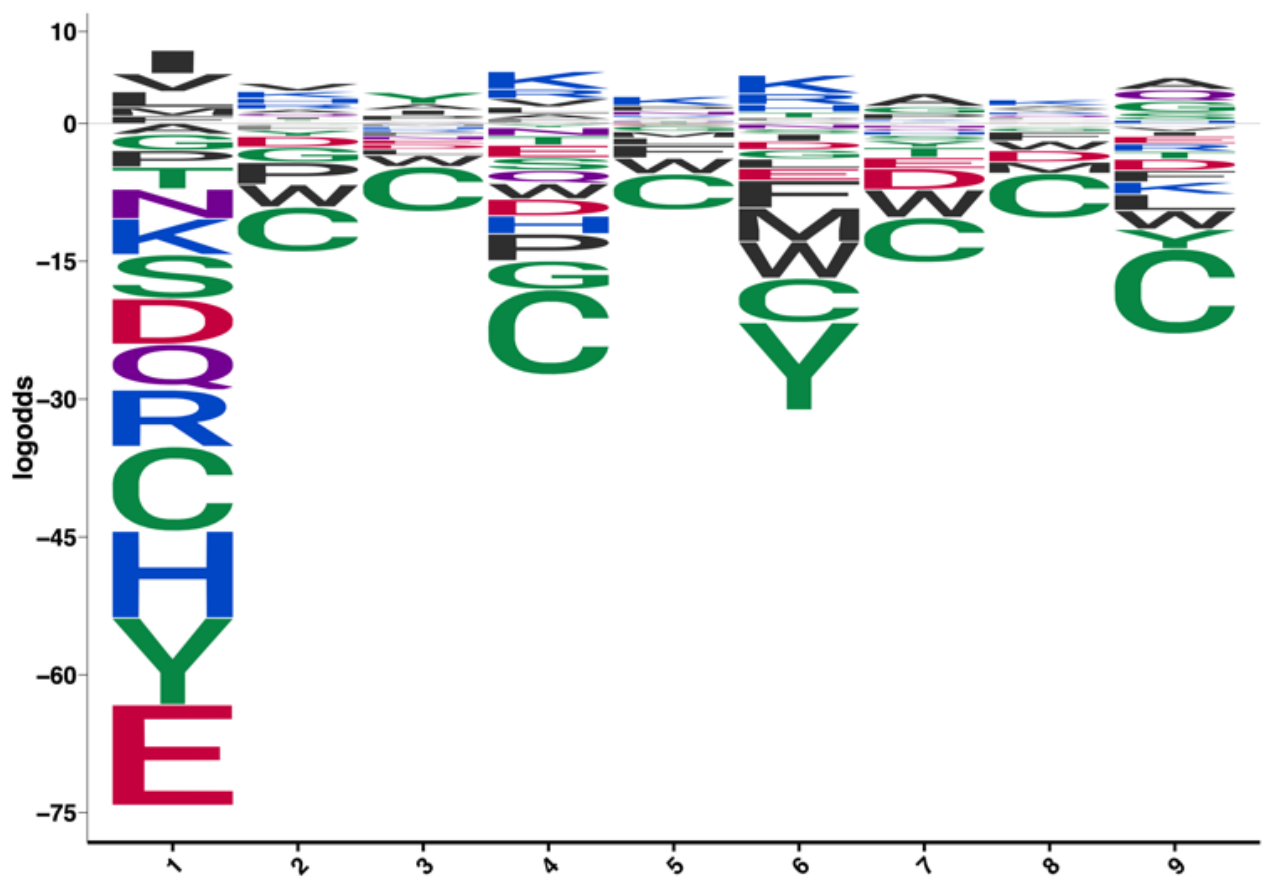

Rebuttal Fig 1. Kullback-Leibler motif for HLA-DRB1*08:04.

We modified the manuscript as follows:

*"We also calculated log-odds ratio values for each amino acid at each position, defined as the $\log_2$ of the amino acid frequency at that position divided by its background probability (i.e. its expected frequency under the background distribution). For the highly promiscuous HLA-DRB1*08:04 allele, most values clustered tightly around zero, indicating that amino acid usage at most positions is close to the background distribution. In contrast, the low-promiscuity HLA-DRB1*16:02 allele showed a broader distribution with a pronounced peak at negative values, reflecting stronger deviations from background at specific positions (Figure 2C).*

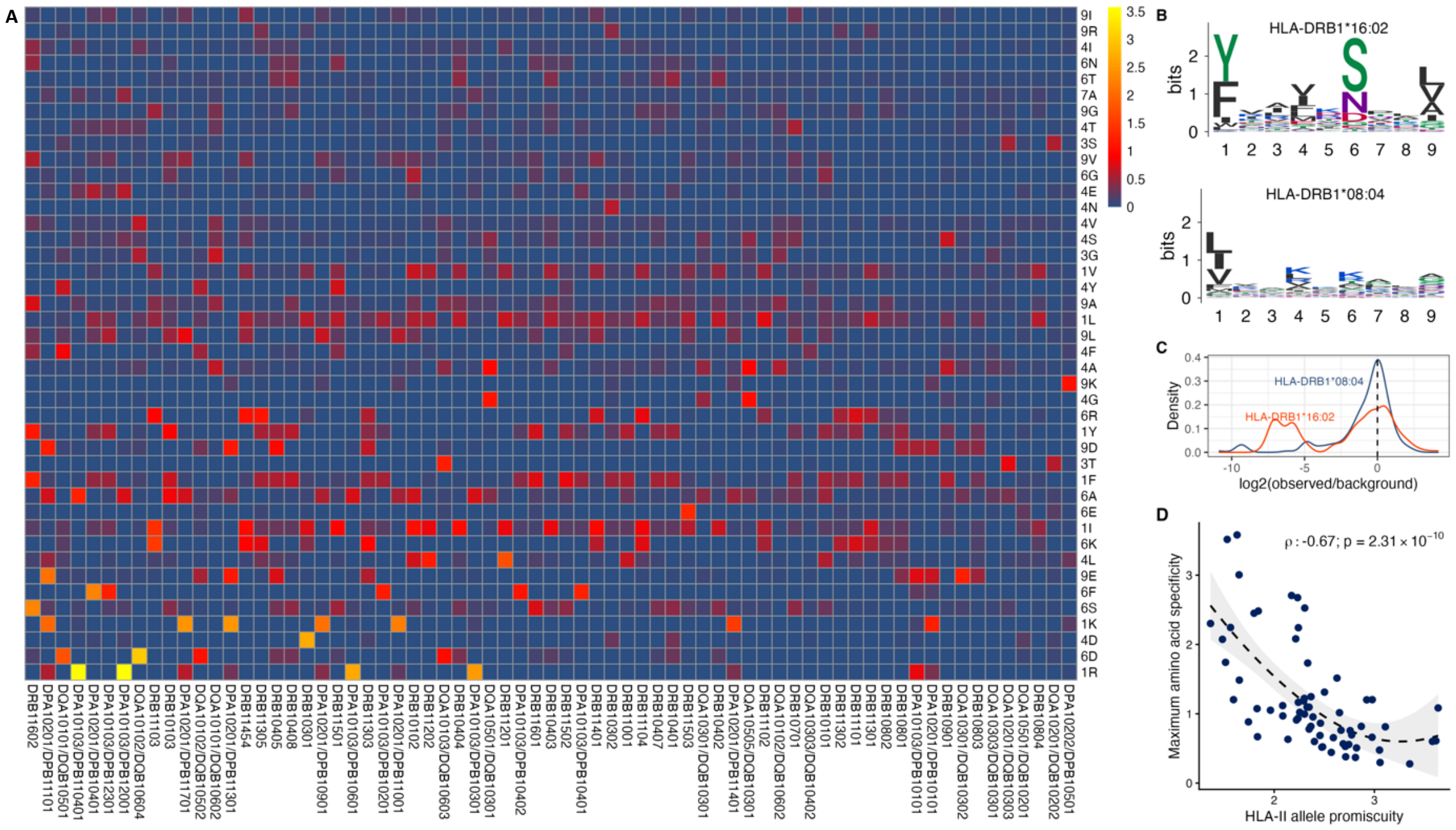

***Figure 2. Pr captures the amino acid specificity of HLA-II alleles. A)** The heatmap indicates the specificity of HLA-II molecules for different amino acids at different positions of the binding core color-coded. HLA molecules are ordered by increasing Pr. The values represent numerical values (in bit) in binding logos (see Methods for details). The first and second character in row names indicate positions and amino acids, respectively. Rows are ordered by the maximum specificity value for the given amino at the given position. Only those position-amino acid combinations are shown that reach at least 0.4 for at least one HLA-II allele. **B)** Representative binding logos of HLA-II alleles with high (bottom) and low (top) Pr. The vertical axes indicate information content in bits. **C)** The plot shows the density of log-odds ratio values for the same alleles as in panel B. **D)** Alleles with high Pr exhibit generally low amino acid specificity. The maximum amino acid specificity, defined as the maximum value in sequence logo is shown as a function of Pr (n = 69 alleles). Spearman's correlation coefficient and p value of a two-sided correlation test is indicated."*

"Fig 3A: The text describing the data processing to get from the 1506 associations identified between antibody-bound antigens and HLA-II alleles to the categorisation of 'positive' and 'negative' associations is difficult to follow (in saying that you "focussed on these identified associations" the presence of negative associations doesn't seem possible). The methods help to clarify the how antibody is the dependent variable, but this is just not as clear in the results section text. Can the authors give some numerical details as to the number of HLA-II alleles in the dataset (presumably around 22 or 23 judging by the dots in Figures 3B/C and Extended data figure 4) and the number of positive and negative associations detected for

each (which then gets divided into pathogens and non-pathogens, if my understanding is correct – apologies if not!).”

Thank you for the comment. In the revised manuscript, we have updated the Results section to define positive and negative associations earlier. We have also added an Extended Data Table reporting the number of positive and negative associations for each allele. In the revised manuscript, we write:

“2,815 antigens, with antibodies present in 5-95% of individuals, were included in further analysis, where we identified 1,506 associations between antibody-bound antigens and HLA-II alleles (25). 75% of associations were positive—where carrying the allele is linked to the presence of the antibody—and 25% were negative, where the allele is connected to the absence of the antibody.

		Pathogens			Non-pathogens		
**HLA allele**	***Pr***	$N_{positive}$	$N_{negative}$	$\frac{N_{positive}+1}{N_{negative}+1}$	$N_{positive}$	$N_{negative}$	$\frac{N_{positive}+1}{N_{negative}+1}$
DQA1*01:01-DQB1*06:02	1.834	6	31	0.219	1	2	0.667
DQA1*03:01-DQB1*03:01	2.570	32	16	1.941	2	5	0.500
DPA1*02:01-DPB1*04:01	1.564	0	0	1.000	0	0	1.000
DQA1*01:02-DQB1*06:04	1.650	51	21	2.364	23	1	12.000
DQA1*01:01-DQB1*05:01	1.515	20	39	0.525	2	8	0.333
DQA1*01:02-DQB1*06:02	2.688	34	30	1.129	22	3	5.750
DQA1*03:01-DQB1*03:02	2.979	36	17	2.056	3	4	0.800
DQA1*05:01-DQB1*03:01	2.328	43	12	3.385	6	3	1.750
DQA1*02:01-DQB1*03:03	3.109	22	0	23.000	12	0	13.000
DQA1*05:01-DQB1*02:01	3.352	98	20	4.714	12	4	2.600
DQA1*01:03-DQB1*06:03	2.301	43	5	7.333	5	0	6.000
DPA1*02:01-DPB1*01:01	2.923	20	4	4.200	1	0	2.000
DQA1*02:01-DQB1*02:02	3.613	23	0	24.000	6	0	7.000
DRB1*01:01	2.721	10	27	0.393	1	6	0.286
DRB1*03:01	2.173	79	12	6.154	13	2	4.667
DRB1*04:01	2.483	25	7	3.250	2	2	1.000
DRB1*04:04	2.303	9	5	1.667	0	0	1.000
DRB1*07:01	2.709	21	0	22.000	4	0	5.000
DRB1*08:01	2.821	3	1	2.000	3	0	4.000
DRB1*11:01	2.758	8	0	9.000	9	0	10.000
DRB1*13:01	2.773	42	4	8.600	3	0	4.000
DRB1*13:02	2.744	31	1	16.000	15	0	16.000
DRB1*15:01	2.222	22	26	0.852	15	3	4.000

Extended Data Table 1. The promiscuity and the number of positive and negative associations for HLA-II alleles.”

“Is there a figure for the pathogens endemic to the Netherlands to go with the Odds Ratio? Was this derived when considering the positive or negative associations or neither?”

Because the number of significant associations is orders of magnitude lower than the number of non-significant ones, it would be difficult to visualize them using pie charts or stacked bar charts. Instead, we provide the contingency table and report in the text the percentage of significant associations for endemic pathogens and for other associations. Both positive and negative associations were included in this analysis.

Class	Significant	Non-significant
**Endemic pathogens**	990	44860
**Other taxa**	513	51777

We modified the text as follows:

“As expected, statistically significant associations were dominated by proteins from common pathogenic microbes endemic to the Netherlands according to the GIDEON database (13, 26) (2.16% and 0.98% of associations were significant for endemic pathogens and other taxa in the dataset, respectively. OR: 2.23, two-sided p value of Fisher’s exact test < $2.2x10^{-16}$).”

“Extended data figure 4 is only the LLD cohort – are both cohorts represented in figure 3B/C? At which point was DQ excluded from these analyses? Fig 3D?”

We apologise for the lack of clarity. In the original manuscript, Extended Data Figure 4 includes only the LLD cohort, whereas Figure 3B/C shows associations obtained using both cohorts. In the revised manuscript, we also included the 1000IBD cohort in Extended Data Figure 4. HLA-DQ was excluded only from the individual-level analysis (Figure 3D) because of the limited number of individuals with promiscuity values for all their DP, DQ and DRB1 molecules. For clarity, we have now explicitly stated this in the revised manuscript.

*“Would the authors predict promiscuous or fastidious HLA-II to better protect from autoimmune diseases? DRB1*15:01 (associated with MS) looks to have relatively low promiscuity… Could there be negative consequences associated with highly promiscuous alleles and unwanted immune responses (beyond susceptibility to emerging diseases)?”*

This is an important point. On the one hand, our previous work on HLA class I molecules showed that high promiscuity can reduce the immune system’s ability to discriminate between self-peptides and their mutated counterparts. On the other hand, it has been proposed that broad HLA presentation of peptides implicated in autoimmune disease may lower the risk of autoimmunity by promoting clonal deletion of self-reactive T-cell clones in the thymus during T-cell maturation. Further studies are needed to resolve these potentially opposing effects. We now summarize this in the Discussion section of the revised manuscript as follows:

“Promiscuous HLA-II molecules may present a larger fraction of self-antigens, which in turn could increase susceptibility to autoimmune diseases. On the other hand, broad HLA presentation of self-peptides could also enhance clonal deletion of self-reactive T cells in the thymus and thereby reduce autoimmune risk (46). Disentangling these opposing effects will require future studies.”

The authors have considered multiple variables in their analyses of the UK biobank and LLD/IBD cohorts including age, sex, presence of immunodeficiencies, ancestry, and known

HLA associations with given diseases to show that HLA-II promiscuity still significantly shapes antibody production and/or disease risk. The tests applied and resulting significances are provided for all data. The methods appear comprehensive. Notably, while the original number of samples from the various databases has typically been quoted in the text, the final sample number used for analyses is often located only in the figure legends or methods. Greater transparency as to these final sample numbers in the text would assist in understanding their workflow and analyses.

Thank you for the suggestion, in the revised manuscript, we aimed to increase transparency by indicating sample numbers at appropriate sections.

Overall, this work has the potential to be quite significant to the field through deepening our understanding of, and appreciation for, the consequences of allelic variation among HLA-II molecules. The suggested role of generalist and specialist (promiscuous and fastidious) HLA alleles as being directed towards common or emerging pathogens, respectively, has previously been suggested for HLA-I alleles, but here the authors have applied this concept to HLA-II alleles (which are probably the lesser studied class in terms of polymorphism), and have further provided data to support this hypothesis. Importantly, the authors skilfully link this HLA-II promiscuity to antibody production and to the risk of disease, suggesting that peptide-binding repertoires can have broad impacts on multiple aspects of the immune system, thus making the work relevant and of interest across many fields.

Reviewer #2 (Remarks on code availability):

I am not in a position to review code.

Reviewer #2 (Remarks to the Author):

In revising their manuscript, “HLA-II peptide-binding diversity shapes humoural immune response and susceptibility to infections,” Magyari and colleagues have endeavoured to address the comments raised by both reviewers, where appropriate, with the addition of new data and supplemental information.

Again, we sincerely thank you for reviewing our manuscript and for your positive assessment.

The additional details regarding HLA-II structure, peptide anchor residues, “core” regions and previous genetic associations with antibody repertoire are helpful and should broaden the accessibility and enhance the importance of the topic to readers. Clarity has also been improved where sought. The authors have considered recalculations and data presentation in response to the comments. The revised heatmap (Fig. 2A) is easier to read with the impact of certain residues now more obvious (although I do wonder whether the addition of axis labels for ‘increasing promiscuity’ and ‘maximum specificity’ could help further improve the readability of the figure). The revised logo plots (Fig. 2B) including all amino acids and new Fig C are clear. The definition of positive and negative associations (line 216-218), along with the addition of Extended Data Table 1, makes the authors’ data crunching and subsequent data presentation more transparent and easier to follow.

Thank you for the suggestion, we have added the labels for the horizonal and vertical axes.

In response to Reviewer 1’s comments the authors have also included additional analyses from an influenza vaccination cohort, which provides a more controlled setting within which to assess the relationship between HLA-II promiscuity and functional antibody responses (as opposed to the presence of antibodies against linear epitopes identified in the PhIP-Seq databases). Promiscuity was only found to correlate with neutralising antibody/HAI titres in one of three age ranges, making this a thought-provoking add-on. Was HLA-DRB1 promiscuity for the donors in the Fluzone vaccine trial calculated as for those in the UK biobank – ie. calculated by averaging the allele-specific values of the b chains encoded by the HLA-DRB1 gene? This does not appear to be specified in the methods. Presumably all participants had sequencing.

Thank you for highlighting this point. We have calculated HLA-DRB1 promiscuity the same way as for UK Biobank individuals and now we included this in the text:

“Reported titer values represent the average of the individual strain-specific titers. For each individual, HLA-DRB1 promiscuity was calculated by averaging the values of the two DRB1 alleles.”

Overall, the authors have considered each of this reviewer’s comments and addressed them sufficiently.

Reviewer #2 (Remarks on code availability):

I am not in a position to review code.

Reviewer #3 (Remarks to the Author):

1) Reviewer 1's major criticism, expressed in several places, is that the authors appear to have selectively presented data that support their conclusions:

"A primary concern is that the three segments of the study appear to have been completed in a manner that selectively support the contribution of promiscuity towards controlling infection."

"The antibody recognition data of antigens from pathogens is also very selective in regards to the antigens tested, the epitopes from the antigens that were tested, and the linear nature of the epitopes"

"In regards to the control of infections, the study would be strengthened were high Pr HLA II analyzed with diseases such as influenza, Mtb, COVID, C. difficile, malaria, and dengue - broader antibody recognition might be expected to exacerbate secondary dengue infections and these diseases draw more attention that viral warts"

The authors disagree with this characterization:

"We disagree with the implication of confirmation bias. Our dataset selections were driven by availability, scale, and relevance—not by outcome."

"Our study was explicitly designed to minimize bias and maximize representativeness at every level. … Contrary to the reviewer's concerns, implementing the suggested changes (focusing only on anchoring positions when calculating promiscuity; selecting alleles based on population prevalence; focusing only on certain disorders, etc.) would in fact increase selectivity and potential bias, undermining the strength of our comprehensive approach."

In my opinion the study is not biased in the way that Reviewer 1 suggests, and the authors' rebuttals generally are convincing. Reviewer 1 seems concerned that only selected pathogens and disease associations are presented, while others that do not align with the conclusion are ignored. The authors appear to have conducted an unbiased analysis with careful consideration of statistical issues such as multiple comparisons, and have presented only the associations resulting from this analysis that are statistically significant. However, this is not clearly stated in the manuscript, and in many places that manuscript is written in such a way as to suggest that the "cherry-picking" concerning reviewer 1 might have occurred. These ambiguities have not been addressed in the revised manuscript and might be source of misunderstanding as the authors suggest.

For example:
"Notably, for many positive associations, we found that HLA-II molecules exhibit a distinct ability to present specific segments of antigens, underscoring the complex relationship between HLA-II alleles and immune recognition (lines 220-223, emphasis added)."

"After adjusting for multiple hypothesis testing, we identified eight disease groups whose occurrence decreased with increased HLA promiscuity (Figure 4D). These

associations were particularly strong for viral and bacterial skin infections, sexually transmitted diseases (dominated by genital warts), lower respiratory tract infections, and mycoses. (lines 309-313,emphasis added)"

"Additionally, this protective effect was significant for viral warts of the skin, also caused by HPV, with a 15.7% reduced risk. Similar reductions were observed for herpes zoster (14.3% reduced risk), caused by the varicella-zoster virus, and for local skin and soft tissue infections, primarily bacterial in origin (14.1% reduced risk) (lines 317-320, emphasis added)"

"Higher HLA-DRB1 promiscuity decreases the lifetime incidence of multiple infections. The table summarizes the Cox proportional hazard models on the incidence of various disorders, treating HLA- DRB1 promiscuity as a continuous variable. (lines 389-391, legend to Figure 4D, emphasis added)"

In each case no rationale was presented for selection of the associations, disease groups, protective effects, or multiple infections highlighted. Examination of the table presented in Figure 4D suggests that rather than cherry picking the authors seem to have considered all of the conditions in the entire analysis, ranked them by their hazard ratio, and selected only those with false discovery rates < 0.05. If this is correct that should be clearly stated in manuscript. If not, the authors should in each case provide the rationale for selection of the particular pathogens, diseases, etc highlighted.

We thank the reviewer for reviewing our manuscript. We are pleased that our rebuttals were generally convincing in addressing the concern regarding confirmation bias. As suggested, we have revised and clarified the text to avoid any impression of cherry-picking:

"We then comprehensively reviewed participants' medical records for evidence of infectious diseases, focusing on those affecting both sexes and most likely caused by common pathogens (see Extended Data Tables 4 and 5 for list of included ICD10 codes)."

"Next, we examined how HLA-II promiscuity influences the cumulative incidence of the infectious disease categories included in the previous analysis. We incorporated data from both inpatient and outpatient medical records, including general practitioner data."

"These associations included viral and bacterial skin infections, sexually transmitted diseases (dominated by genital warts), lower respiratory tract infections, and mycoses. The strongest association was observed in the "other sexually transmitted diseases" group, predominantly linked to genital warts caused by human papillomavirus (HPV)."

"Notably, in the original study, we found for many positive associations that HLA-II molecules exhibit a distinct ability to present specific segments of antigens, underscoring the complex relationship between HLA-II alleles and immune recognition."

A conventional presentation of this sort of data is a volcano plot, where the reader could see for themselves all of the relevant data together with effect size and uncertainly values, and how any selected items fit into the overall distribution. The authors might consider adding such a plot, where the Cox HR value would take the place of the conventional “fold increase” axis and the FDR take the place of the conventional p-value axis.

Thank you for the suggestion, we created a volcano plot and replaced Figure 4D with it (the numerical data is now found in Extended Data Table 4):

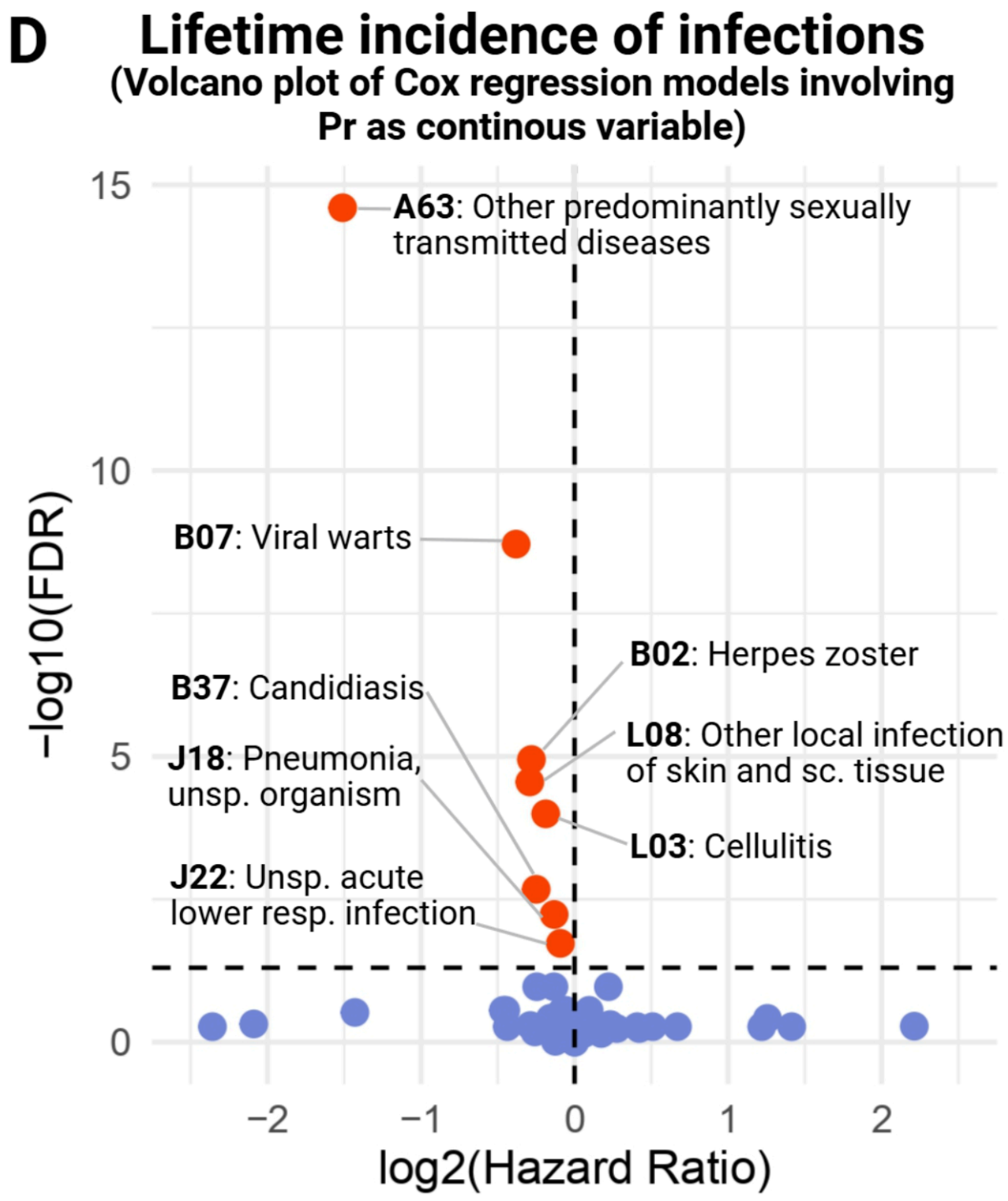

“…D) Higher HLA-DRB1 promiscuity decreases the lifetime incidence of multiple infections. The volcano plot shows the log2 of hazard ratios and the −log10 of adjusted p-values from Cox proportional hazards models for the incidence of various disorders, with HLA-DRB1 promiscuity treated as a continuous variable. p values were corrected using the Benjamini-Hochberg method (41). The horizontal dashed line represents 0.05 FDR value, significant values are highlighted with red colour. n = 1676, 16293, 14712, 17615, 12217, 31087, 36694 and 59797 individuals diagnosed with A63, B07, L08, B02, B37, L03, J18, and J22, respectively. For the full list of diseases and corresponding values, see Extended Data Table 4…”

2) In a similar vein, Reviewer 1 notes that the authors are not very systematic or precise in how they describe the main results of the study:

In summary, the study seems to have selected particular data from each of three categories to arrive at the conclusion that HLA II peptide ligand promiscuity is important for controlling infections while omitting a broad spectrum of data that might not support this conclusion.”

“HIV might not be categorized as a newly emerging infectious disease.”

Indeed, the main conclusion of the study is stated clearly in the title of the manuscript:

“HLA-II peptide-binding diversity shapes humoral immune responses and susceptibility to infections” (lines 1-2)
but in the abstract the association between HLA-II binding diversity and susceptibility to infections has been extended to overall health outcomes:

“demonstrating that a broad peptide-binding repertoire of HLA-II is linked to antibody production against pathogen-associated proteins and more favourable health outcomes (lines 27-27).

What actually was observed is that the breadth of the HLA-II peptide binding repertoire was positively associated with humoral responses to some pathogens but not others, and was positively associated with some positive health outcomes but not significantly associated with most of the positive health outcomes investigated. This is clearly stated in the first paragraph of the discussion:

Individuals carrying HLA-II variants with a broad peptide-binding repertoire are more likely to produce antibodies that target pathogen-associated proteins and are less likely to experience severe infections from common pathogens, as reflected in their medical records. Additionally, high HLA-II promiscuity was associated with a lower cumulative incidence of a wide range of infections. (lines 423-427)

Thank you for highlighting this. In the revised manuscript, we have modified the text of the abstract as follows:

“Here, we integrated data on HLA-II immunopeptidomics and antibody responses in ~1,500 individuals, demonstrating that a broad peptide-binding repertoire of HLA-II is linked to antibody production against specific pathogen-associated proteins and more favourable outcomes in several common infections. In the UK Biobank, individuals carrying such HLA-II molecules had a reduced risk of severe infections and up to a 45% lower incidence of diseases caused by common pathogens.”

In fact, the authors call out a HIV as a prominent exception to the positive health association:

Notably, we found that the low promiscuity HLA-II variants provide protection against HIV, one of the most relevant emerging viruses (lines 444-445, also Fig 4G)

Obviously, protection against HIV would be considered a favorable health outcome, but here the association is opposite the statement in the abstract. The authors should take to care to summarize their findings carefully and avoid broad generalizations beyond what is demonstrated by their data and careful statistical analysis.

Reviewer 1 also notes that “HIV might not be categorized as a newly emerging infectious disease”

apparently in regard to the explanations provided for the HIV outlying results:

On the other hand, HLA-II molecules with a low promiscuity level may specifically target emerging, but relatively rare pathogens (31) (line 221-223).

However, this does not imply that low-promiscuity HLA alleles are without benefit. These alleles may play a critical role in defending against emerging pathogens (lines 327-328)

Notably, we found that the low promiscuity HLA-II variants provide protection against HIV, one of the most relevant emerging viruses, which is in line with previous results on HLA-I (45). (lines 450-452)

In fact, unlike HIV several other emerging diseases such as Dengue, Ebola, human alphaviruses, SARS, MRSA, and West Nile virus were not found to be negatively associated with HLA-II binding promiscuity (Extended Data Table 2). A conclusion about emerging viruses would have been warranted if the authors had performed a specific test of emerging versus established viruses and observed a significant association, but in this absence there is no data presented that supports any conclusion about recently emerging viruses. The authors could just have easily characterized the result as pertaining to small positive strand RNA viruses, or T-cell tropic viruses, or chimp-derived zoonotic viruses.

A statement that HLA-II peptide binding diversity is associated with protective humoral responses against some but not all viruses investigated, with the specific mechanisms to be determined, would be a more appropriate characterization of the results than any conclusions about recent versus past viral emergence into humans.

Thank you, we have modified the text as follows:

“On the other hand, HLA-II molecules with a low promiscuity level may specifically target evolutionary novel, and relatively rare pathogens”

“These alleles may play a role in defense against less common pathogens that are markedly distinct from those usually associated with common infections. (31) (Figure 4F). To explore this, we analyzed the cumulative incidence of HIV infection (36). HIV-1, a retrovirus in the kingdom Pararnavirae, lies on a deeply divergent branch of the RNA virus phylogeny relative to Orthornavirae viruses that cause most common human infections (37), and is therefore expected to have a highly distinct antigenic repertoire (38, 39).”

We present the original idea only in the Discussion section and emphasize that it is supported by limited data.: “While this result suggests potentially distinct roles for HLA-II alleles with high and low promiscuity, the finding is limited by the focus on a single pathogen and therefore warrants further systematic investigation across less frequent and evolutionarily novel pathogens.”

3) Another concern of reviewer 1 is the use of the full nine-residue peptide binding preferences instead of focusing on the major MHC-peptide contact positions:

“It would have been helpful to have the study focused on P1 P4 P6 and P9 anchors as these are the portion of the peptide ligand that interact with HLA II. Do the authors draw the same conclusions when only the P1 P4 P6 and P9 anchors are assessed for promiscuity”

The authors have addressed this concern clearly in the revised manuscript, rationalizing the use of the full 9-residue motif, and providing in the rebuttal new data that an analysis restricted to P1,4,6,9 yields similar results.

However, the authors might consider addressing a related issue not specifically mentioned by reviewer 1. In their analysis of HLA-II peptide binding preferences the authors seem to imply that they are providing new information about the ability of various HLA-II molecules to bind diverse peptide sequences:

“To assess the robustness of the Pr values and minimize potential bias arising from differences in experimental setups across immunopeptidomics studies, we re-calculated Pr using two independent subsets of the original dataset (n = 102 and n = 21 samples). (lines 112-115).”

“The reliability of the calculated Pr values was further validated using data from two independent immunopeptidomics studies, which focused on HLA-II-presented peptides from adeno-associated virus (AAV) and SARS-CoV-2 (22, 23). (lines 119-121).”

In every case the calculated Pr values were determined for lists of peptides derived from previously-reported large immunopeptidomics datasets characterizing multiallelic samples. In these lists the peptides were assigned, either in the original study (ref 22) or retrospectively by the authors (ref 23), to the individual HLA alleles present in each sample using a NetMHCIIpan, a neural-network based MHC-peptide binding prediction algorithm. Thus the diversity profiles for each allele are determined largely by the prediction algorithm. Moreover, the authors only consider HLA-DRB1 alleles, and not the genetically linked HLA-DRB3, HLA-DRB4, and HLA-DRB5 allele which are present in most samples. These alleles have different specificities than the genetically linked HLA-DRB1 alleles (Kaabinejadian et al Front Immunol PMID:35154160), and the authors’ comingling of their peptidomes introduces error into the calculated Pr values. Datasets derived from monoalleleic samples are available (Abelin et al Immunity 2019 PMID: 31495665) and would avoid these problems, but these were not utilized. The authors should consider mentioning in the limitation section of the manuscript that the Pr values depend on HLA assignments derived from the allele assignment algorithm NetMHCIIpan.

We have included a section about this limitation in the revised manuscript:
“A limitation of our Pr estimates is that they are derived from peptide lists assembled from previously published multiallelic HLA-II immunopeptidomics datasets in which peptides were assigned to individual alleles using NetMHCIIpan (either in the original studies or retrospectively). As a result, the inferred diversity profiles for each HLA-DRB1 allele—and consequently the Pr values—depend on the performance and potential biases of this prediction-

based allele assignment. Future work should validate Pr estimates using monoallelic data."

4) Another concern of reviewer 1 is that the original study was based on the phage display platform PhIP-Seq, which might not reflect biologically relevant antibody binding:

"The antibody recognition data of antigens from pathogens is also very selective in regards to the antigens tested, the epitopes from the antigens that were tested, and the linear nature of the epitopes. The neutralizing capacity of these antibodies is unknown as is the affinity of these antibodies for their targets."

The authors helpfully respond by extending the analysis to an existing dataset of neutralizing antibodies induced by influenza vaccination, showing the same allele promiscuity effects as the PhIP-Seq analysis. As part of this analysis the authors uncovered an interesting age-dependent change in the association of HLA-II peptide binding promiscuity with neutralizing antibody titer. The authors might be interested in a very recent paper using PhIP-Seq to analyze anti-influenza responses by age (Olin et al Nat Immunol 2026 PMID: 41699059), which also observed age-dependent changes in epitope specificity (although not addressing HLA-II binding diversity as in the present work).

Thank you for suggesting this paper. We found it very interesting and have included it in the Discussion section:
"Moreover, high HLA-II promiscuity was associated with higher influenza vaccine response rates among individuals aged 60–79 years. Notably, a recent study reported age-related differences in the antibody repertoire targeting influenza A virus—including reduced reactivity to rapidly evolving epitopes and increased reactivity to conserved epitopes"

Other more minor concerns of reviewer 1 were addressed clearly in the rebuttal and/or revised manuscript.

REVIEWERS' COMMENTS

Reviewer #2 (Remarks to the Author):

In the next revision of their manuscript, "HLA-II peptide-binding diversity shapes humoral immune response and susceptibility to infections," Magyari and colleges have worked to address the additional comments from reviewers with clarification of the text and some figure adjustments.

The comments from reviewer two have been sufficiently addressed through minor adjustments of a figure and the text.

Reviewer three has carefully assessed the comments raised by reviewer one and the authors have endeavoured to address them. To dispel concerns regarding the "cherry-picking" of data, the authors have softly adjusted the text to demonstrate that their examples are just that, examples (eg. Use of the word "including"). However, there is no distinct rationale for why A63, B02, B07 and L08 have been analysed in Fig4E (which I think is what reviewer three was after). The addition of the suggested volcano plot provides a welcome visualisation of the impact of promiscuity on infection, and one can see that the four groups selected for the following analyses are the most significant, but maybe that just needs to be said in the text.

We thank the reviewer for the comment. According to the suggestion, we have complemented the main text and the text of the figure legend as follows:

"…These associations included viral and bacterial skin infections, sexually transmitted diseases (dominated by genital warts), lower respiratory tract infections, and mycoses. To further illustrate the strongest associations, we focused on the four most significant disease groups and compared cumulative incidence between individuals in the top and bottom 10% of HLA-DRB1 promiscuity (Figure 4E). The strongest association was observed in the "other sexually transmitted diseases" group…"

"E) HLA-DRB1 promiscuity groups are associated with up to 45% decreased infection incidence rates. Representative plots show cumulative disease incidence for the four most significant associations in panel D, comparing patients in the highest and lowest deciles of HLA-DRB1 promiscuity…"

The authors have also modified their characterisation of HIV as an "emerging" virus, now referring to it as evolutionarily novel, thus preventing generalisations about emerging viruses and HLA-II promiscuity from being made. The adjustments to the abstract, considering that HIV is an exception to the benefits of promiscuity found in some other infections as flagged by the reviewer (now stating favourable outcomes for "several common infections" instead of "health outcomes") are appropriate and more accurate.

The authors have added subheadings to their discussion, including a section on limitations where they have made note of the biases of the predictive allele-based assignments of peptides (as suggested by the reviewer), although they do not make mention of DRB3, 4 or 5 alleles that have not been represented in their dataset. They have also added reference to the influenza paper suggested by the reviewer.

Thank you. Now, we have included a statement about DRB3, 4, and 5 in the revised manuscript:

"Future work should validate Pr estimates using monoallelic data. Furthermore, some HLA-II haplotypes contain not only DRB1 but also DRB3, DRB4, and DRB5 genes. The

peptide-binding repertoires of these additional genes could potentially be incorporated into peptide-binding repertoire calculations in future studies."

Overall, the authors have addressed the concerns of each of the reviewers throughout the various iterations of the manuscript.

Reviewer #2 (Remarks on code availability):

I am not in a position to review code.

Reviewer #3 (Remarks to the Author):

The revised manuscript addresses each of the four issues brought up in my review (and initially noted in reviewer 1's comments on the original manuscript).
